# Unveiling Solvent-Mediated Mechanochemical Cocrystallization Pathways by *In Situ* CLASSIC NMR Spectroscopy

**DOI:** 10.1021/acs.molpharmaceut.5c01909

**Published:** 2026-04-27

**Authors:** Anna M. Gołkowska, Maciej Nowak, László Fábián, Dinu Iuga, Franziska Emmerling, Karol P. Nartowski, Bożena Karolewicz, Yaroslav Z. Khimyak

**Affiliations:** † Department of Drug Form Technology, 49550 Wroclaw Medical University, Borowska 211A, 50-556 Wroclaw, Poland; ‡ School of Chemistry, Pharmacy and Pharmacology, 6106 University of East Anglia, Norwich Research Park, Norwich NR4 7TJ, United Kingdom; § Department of Physics, 2707 University of Warwick, CV4 7AL Coventry, United Kingdom; ∥ Federal Institute for Materials Research and Testing (BAM), Richard-Willstätter-Strasse 11, 12489 Berlin, Germany

**Keywords:** NMR spectroscopy, pharmaceutical cocrystals, polymorphism, mechanochemistry, liquid-assisted grinding, solvents effect

## Abstract

Although mechanochemical synthesis offers a sustainable way to produce solid forms of pharmaceutical compounds, the molecular-level mechanisms that govern solvent-mediated transformations remain largely unexplored. In this study, we present an *in situ* solid-state nuclear magnetic resonance (NMR) approach to directly monitor the evolution of liquid-assisted grinding reactions under magic-angle spinning conditions. Using a modified CLASSIC NMR protocol, we tracked the cocrystallization of two model systems, theophylline–benzamide and metronidazole–gallic acid, in the presence of solvents with different levels of polarity. This method allows us to observe both the solid and liquid phases within the reaction environment simultaneously, revealing transient intermediates, hydrate formation, and solvent-dependent polymorphic outcomes. Comparisons with time-resolved *in situ* X-ray diffraction confirm the complementary nature of NMR in capturing mechanistic details that are otherwise inaccessible to a diffraction-based analysis. This work establishes solid-state NMR as a powerful and accessible *in situ* tool for investigating the effects of solvents in mechanochemical synthesis, thereby advancing our molecular understanding of polymorphic control and reaction pathways.

## Graphical abstract

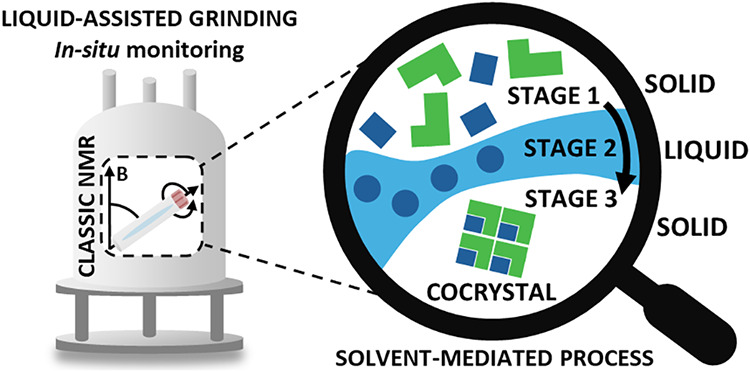

## Introduction

Pharmaceutical cocrystals are multicomponent solid-state materials^1^ that attract significant academic and industrial interest, due to their ability to alter the properties of an active pharmaceutical ingredient (API) without affecting its therapeutic activity.^2^ Cocrystals are prone to exhibit polymorphism,^3^ i.e., the API and a coformer adopt different packing arrangements within the crystal lattice. This phenomenon results in the differences between polymorphic forms, including their solubility or bioavailability,^4^ which constitute critical quality attributes of a pharmaceutical product. Therefore, techniques that allow precise control over polymorphic outcomes are of growing significance and in this regard mechanochemistry has attracted increasing attention.^5–7^ In particular, liquid-assisted grinding (LAG) has gained recognition in this field, as the addition of solvents with different properties (e.g., polarity) and their amount can influence not only the polymorphic outcome^8–11^ of a reaction but also its kinetics, yield, and product purity.^10,12^

The mechanisms underlying the formation of polymorphic cocrystals are not yet fully understood, and in this context, *in situ* reaction monitoring has proven to be a valuable tool. Time-resolved *in situ* powder X-ray diffraction (TRIS-PXRD) represents the gold standard for studying mechanochemical reactions, including cocrystallization^8,13–20^ and other organic reactions.^20–23^ However, as it detects only changes in crystalline solid-state components, its applicability is limited when solvents, such as those in LAG, participate in the reaction mechanism. Furthermore, the requirement of high-energy synchrotron X-ray radiation restricts its accessibility.

In contrast, NMR spectroscopy remains underrepresented in mechanochemistry, being primarily employed to analyze reaction products upon dissolution (solution-state NMR), which can induce undesired changes in sample identity or composition.^24^ Solid-state NMR spectroscopy enables direct analysis of the sample,^24^ which is a significant advantage, particularly for polymorphic systems. Nonetheless, reaction monitoring studies using solid-state NMR have predominantly relied on *ex situ* analysis of samples withdrawn from the reaction vessel at set time intervals,^25,26^ with only a few reports addressing *in situ* mechanistic studies. This limited application likely stems from the challenges associated with integrating an operating ball mill into a solid-state NMR spectrometer, akin to TRIS-PXRD setups.^20,27,28^ The only reported case, by Schiffmann et al., required a home-built solid-state NMR probe.^29^ This setup allowed static ^31^P solid-state NMR measurements, which were used to monitor zinc phenylphosphonate formation. Unfortunately, combining such a probe with magic-angle spinning (MAS) NMR, necessary for high-resolution spectra, does not appear feasible. Interestingly, the centrifugal pressure generated in an NMR rotor under MAS conditions has been shown to induce mechanochemical reactions,^25,29^ including cocrystal formation.^30–33^ These studies highlight the potential of solid-state NMR for revealing unexpected intermediates, tracking product formation, and following the real-time reaction kinetics. Despite these advances, gaps remain in the field. Only the study by Xu et al. focused on a polymorphic cocrystal system,^30^ however, employing ^31^P NMR, which is not directly transferable to each pharmaceutically relevant system. Moreover, most reports have investigated reactions under dry conditions, simulating neat grinding (NG), with only one liquid-assisted grinding (LAG) study reported by Xu et al.^30^ Their work explored the effect of acetonitrile addition on reaction rate but did not examine the behavior of the liquid phase itself, focusing solely on solid-state transformations.

NMR spectroscopy offers the unique capability of probing both solid- and liquid-state components within a reaction mixture. Experimental protocols and pulse sequences such as CLASSIC NMR (combined liquid- and solid-state *in situ* crystallization NMR)^34,35^ and SASSY NMR (simultaneous solid and solution spectroscopy)^36^ have been developed for this purpose. However, to the best of our knowledge, these approaches have not yet been applied to the monitoring of mechanochemical reactions or cocrystallization processes. Such studies would be of considerable importance, as solvents are postulated to play an active role in the mechanism of LAG-induced reactions and mechanochemical cocrystallization.^12,37,38^

In this study, we used magic-angle spinning (MAS) conditions to mimic the mechanochemical cocrystallization of two model pharmaceutical cocrystals—theophylline with benzamide (TP:BZ) and metronidazole with gallic acid (MNZ:GAL)—both known to exist in two polymorphic forms. Experiments were conducted at natural isotope abundance using ^1^H and ^13^C NMR. A standard solid-state NMR setup combined with a modified CLASSIC NMR protocol was used to monitor solid-state transitions occurring during cocrystallization and to evaluate the role of the solvent in the reaction. Three solvents of different polarity (water, methanol, and toluene) were investigated at two distinct solvent-to-powder ratios (η).^12^ To precisely deliver the solvent and to synchronize the start of data acquisition with the initial contact of the solvent and powder mixture, sealed glass capillaries were employed. This approach, originally introduced by Harris et al.^39^ for studying water absorption^40^ and hydration^41–43^ processes, has not previously been applied to cocrystallization studies or LAG-like reactions. The acquired NMR data were compared with results obtained from LAG experiments, PXRD, TRIS-PXRD and solubility studies to assess the accuracy and reliability of the solid-state NMR observations. This approach allowed us to monitor the evolution of complex, multiphase reactions and to elucidate their molecular-level mechanism, revealing the crucial role of the solvent in mediating the process and of intermediate phases such as *in situ* formed hydrates.

## Experimental Section

### Materials

Theophylline anhydrate (TP, ≥99.0%) and benzamide (BZ, 99.0%) were purchased from Pol-Aura (Poland) and Acros Organics (Belgium), respectively. Metronidazole (MNZ, 99.0%) and gallic acid anhydrate (GAL, ≥98.0%) were purchased from Pol-Aura (Poland). Reagents were used without further purification. Deuterium oxide (D_2_O), methanol-*d*_4_ (MeOD), and toluene-*d*_8_ (TOL_d_) used in the study for NMR purposes were purchased from Sigma-Aldrich (Germany). Borosilicate glass capillary tubes (BGCT 1.0, Ø 1.0 mm) were purchased from Capillary Tube Supplies Ltd. (United Kingdom). Solvents used for LAG experiments were deionized water (H_2_O), methanol (MeOH), ethanol (EtOH), 1-propanol (1-PrOH), 1-butanol (1-BuOH), acetonitrile (ACN), *N,N*-dimethylformamide (DMF), dichloromethane (DCM), ethyl acetate (EtOAc), diethyl ether (Et_2_O), toluene (TOL), *n*-hexane (n-HEX), and cyclohexane (c-HEX), which were of analytical grade and purchased from Sigma-Aldrich (Germany). The solvents used for HPLC analysis (H_2_O, MeOH, and TOL) were of HPLC grade and purchased from Sigma-Aldrich (Germany).

### Preparation of the Reference Materials

Theophylline monohydrate (TP-MH) and gallic acid monohydrate (GAL-MH) were obtained using the suspension crystallization method. Theophylline (500 mg) or gallic acid (500 mg) and deionized water (5 mL) were mixed in a closed glass vial, and then the suspensions were heated on a hot plate to 110 °C, cooled to room temperature, and left for 12 h. The obtained crystals were dried on a paper filter directly before the further use of TP-MH or GAL-MH. When theophylline monohydrate or gallic acid monohydrate crystals were intended to be used for NMR spectroscopy studies, deionized water was substituted with D_2_O.

The reference TP:BZ (1:1) cocrystal polymorphs were synthesized mechanochemically using a Fritsch Mini-Mill Pulverisette 23 ball mill, a 10 mL volume zirconium oxide (ZrO_2_) milling jar, and three zirconium oxide (ZrO_2_) milling balls (Ø 10 mm) at 30 Hz for 40 min at room temperature. The equimolar physical mixture of TP (179.39 mg, 1.0 mmol) and BZ (120.61 mg, 1.0 mmol) (TP:BZ PM) was ground to prepare form I (TP:BZ F1m—metastable form).^44^ The experiment was repeated in the presence of the water addition (100 μL, η = 0.33 μL mg^–1^)^12^ to produce form II (TP:BZ F2s—stable form).^8^ The reference MNZ:GAL (1:1) cocrystal polymorphs were synthesized using the above-mentioned setup at 30 Hz for 20 min at room temperature. The equimolar physical mixture of MNZ (150.46 mg, 0.88 mmol) and GAL (149.54 mg, 0.88 mmol) (MNZ:GAL PM) was ground with the addition of water (100 μL, η = 0.33 μL mg^–1^) to prepare form I (MNZ:GAL F1s—stable form).^45^ The experiment was repeated in the presence of methanol (100 μL, η = 0.33 μL mg^–1^) to produce form II (MNZ:GAL F2m—metastable form).^46^

The identity of the starting materials, reference monohydrates, and products of the ball milling procedures was confirmed using PXRD (Bruker D2 Phaser). The experimental data were compared with the patterns calculated for the corresponding structures deposited in the CSD (Cambridge Structural Database) (Figure S1).

### Methods

#### Nuclear Magnetic Resonance Spectroscopy (NMR)

All NMR spectra were acquired using Bruker AVANCE III spectrometer (UK 850 MHz Solid-State NMR Facility at the University of Warwick) with a Larmor frequency of 850.22 MHz for ^1^H and 213.81 MHz for ^13^C. The samples were measured using a 4 mm double-resonance solid-state NMR probe at an MAS spinning rate of 12.5 kHz at 293 K. The alanine-2-^13^C was used as an external reference.^47^ The ^1^H NMR spectra were referenced against the downfield resonance set to 8.5 ppm, and for the ^13^C spectra, the resonance of α-CH was set to 51.1 ppm (both relative to TMS). The ^1^H NMR spectra were recorded using a rotor-synchronized spin echo sequence to suppress the proton background of the probe and the π/2 pulse length was optimized to 3.0 μs. All the ^13^C­{^1^H} NMR (high-power ^1^H decoupling) spectra were acquired with the optimized ^13^C pulse length of 3.0 μs and SPINAL64 decoupling. For the ^1^H–^13^C CP/MAS (cross-polarization/magic-angle spinning) NMR measurements, the RAMP CP (70–100%) pulse sequence and SPINAL64 decoupling were used. The π/2 pulse length was set to 3.0 μs, and the contact time (for CP) was set to 2.0 ms. Reference spectra were collected for the neat components (TP, TP-MH, BZ, TP:BZ, PM, F1m, F2s, MNZ, GAL, GAL-MH, MNZ:GAL, F1s, and F2m) and for the CLASSIC NMR experiments, using the repetition time and number of scans summarized in Table 1. For CLASSIC NMR experiments, two sequences (CLASSIC-S1 and CLASSIC-S2) were used, varying in the number of alternating ^1^H NMR and ^13^C­{^1^H} NMR acquisitions before the first ^1^H–^13^C CP/MAS NMR spectrum was recorded, i.e. ^1^H NMR and ^13^C­{^1^H} NMR repeated 10 times (CLASSIC-S1) or ^1^H NMR and ^13^C­{^1^H} NMR repeated 45 times (CLASSIC-S2). The order and duration of the experiments in both sequences are listed in Figure 2C. The assignment of the ^1^H and ^13^C peaks was carried out based on the ^1^H MAS NMR and ^1^H–^13^C CP/MAS NMR spectra and CASTEP prediction of chemical shifts.

**Table 1:** Pulse Delays and Number of Scans Used during NMR Spectroscopy Measurements^a^

	^1^H NMR		^13^C{^1^H} NMR		^1^H-^13^C CP/MAS NMR	
	d_1_	ns	d_1_	ns	d_1_	ns
Reference spectra	5	8	3	128	60^b^	64
CLASSIC-S1	5	4	3	32	60	64
CLASSIC-S2	5	4	3	32	60	160

a d_1_—repetition time [s]; ns—number of scans; reference spectra: TP, TP-MH, BZ, TP:BZ PM, TP:BZ F1m, TP:BZ F2s, MNZ, GAL, GAL-MH, MNZ:GAL F1s, MNZ:GAL F2m.

b for TP-MH, a pulse delay of 75 s was used.

### NMR Samples

#### TP:BZ-N and MNZ:GAL-N (Neat Powders)

TP (41.86 mg, 0.23 mmol) and BZ (28.14 mg, 0.23 mmol), or MNZ (35.11 mg, 0.21 mmol) and GAL (34.89 mg, 0.21 mmol) were weighed in a 1:1 stoichiometric ratio. The powders were mixed using a benchtop vortexer to ensure the uniform distribution of the starting materials in the physical mixtures and to prevent exposure to mechanochemical grinding (as mixing with a mortar and pestle could provide) prior to placing the sample under MAS conditions. The powders were packed into the 4 mm ZrO_2_ MAS rotors and closed with the Kel-F drive caps.

#### TP:BZ-Solvent and MNZ:GAL-Solvent (Solvents Closed in a Sealed Glass Capillary)

TP (41.86 mg, 0.23 mmol) and BZ (28.14 mg, 0.23 mmol) or MNZ (35.11 mg, 0.21 mmol) and GAL (34.89 mg, 0.21 mmol) were weighed in a 1:1 stoichiometric ratio. The reactants were mixed using a benchtop vortexer to ensure the uniform distribution of the starting materials in the physical mixtures and to prevent exposure to mechanochemical grinding (as mixing with a mortar and pestle could provide) prior to placing the sample under MAS conditions. Subsequently, a glass capillary was filled with 10 μL of a solvent of choice (D_2_O, MeOD, TOL_d_) using an automatic pipet and sealed on both ends with a gas lighter. One (η = 0.14 μL mg^–1^) or two (η = 0.28 μL mg^–1^) capillaries were positioned concentrically inside the 4 mm ZrO_2_ MAS rotor, and the physical mixture (TP:BZ PM or MNZ:GAL PM) was transferred to the rotor. The powder was uniformly distributed around the capillary. The rotor was closed with a Kel-F drive cap secured with a silicone sealant to prevent the leakage of the solvent released during the measurement. Control experiments were also conducted with the individual components (TP, BZ, MNZ, GAL), which were tested according to the same procedure to evaluate their behavior upon contact with a solvent.

#### Grinding

The polymorphic screening of the MNZ:GAL system was performed using a Fritsch Mini-Mill Pulverisette 23 ball mill and custom-designed milling jars comprising a poly­(methyl methacrylate) (PMMA) cylinder and two stainless steel caps.^20^ The mill operated with two stainless steel balls (Ø 6 mm) inside the milling jar at 50 Hz for 60 min at room temperature. The equimolar physical mixture of MNZ (75.23 mg, 0.44 mmol) and GAL (74.77 mg, 0.44 mmol) (MNZ:GAL PM) was ground in the absence (neat grinding, NG) or presence (liquid-assisted grinding, LAG) of 25 μL (η = 0.17 μL mg^–1^) of a solvent (H_2_O, MeOH, EtOH, 1-PrOH, 1-BuOH, ACN, DMF, DCM, EtOAc, Et_2_O, TOL, n-HEX, c-HEX). The identity of the recovered powder was confirmed by PXRD (Bruker D8 Advance). For *in situ* PXRD measurements, the same setup was used with a difference of frequency (30 Hz) and number and size of milling balls (one, Ø 10 mm, stainless steel) used.

The effect of the ratio of solvent volume to sample weight (η) on the cocrystallization outcome was assessed using a Fritsch Mini-Mill Pulverisette 23 ball mill, a 10 mL volume zirconium oxide (ZrO_2_) milling jar and three zirconium oxide (ZrO_2_) milling balls (Ø 10 mm) at 30 Hz for 20 min (if not stated otherwise) at room temperature. The equimolar physical mixture of TP (179.39 mg, 1.0 mmol) and BZ (120.61 mg, 1.0 mmol) (TP:BZ PM) or the equimolar physical mixture of MNZ (150.46 mg, 0.88 mmol) and GAL (149.54 mg, 0.88 mmol) (MNZ:GAL PM) was ground with the addition of 50 μL (η = 0.17 μL mg^–1^) or 100 μL (η = 0.33 μL mg^–1^) solvent (H_2_O, MeOH, TOL). The resulting powders were analyzed using PXRD (Bruker D2 Phaser). The polymorphic screening of the individual components was conducted accordingly. The 300 mg of a powder (TP, BZ, MNZ, or GAL) was ground with the addition of 50 μL (η = 0.17 μL mg^–1^) of a solvent (H_2_O, MeOH, TOL) and the resulting products were subjected to PXRD measurements (Bruker D2 Phaser).

#### Powder X-ray Diffraction (PXRD)

The starting materials and the products of ball milling using a ZrO_2_ jar, as well as the powders remaining after preparing the HPLC samples, were analyzed using a Bruker D2 Phaser diffractometer equipped with a LynxEye detector (Cu Kα radiation; Kα_1_ = 1.5406 Å). Data were collected at room temperature in the range from 5° to 36° 2θ with a step size of 0.02° 2θ and irradiation time of 1 s per step. The X-ray tube operated at 30 kV and 10 mA. After ball milling with jars made of PMMA and stainless steel, the powders were analyzed using a Bruker D8 Advance diffractometer (Cu Kα radiation; Kα_1_ = 1.5406 Å) equipped with the LynxEye XE–T detector. Data were collected over a 2θ range from 5° to 36° using a step size of 0.02° 2θ and an irradiation time of 0.2 s per step.

#### Time-Resolved *In Situ* Powder X-ray Diffraction (TRIS-PXRD)

Time-resolved *in situ* PXRD data were collected at the BESSY II Light Source (Helmholtz-Zentrum Berlin für Materialien and Energie) at the μ-spot beamline.^48^ The position of the mill was adjusted to minimize artificial peak broadening.^20^ Each PXRD pattern was obtained by accumulating the scattering intensities for 5 s (monochromatic radiation, λ = 0.7314 nm). The resulting diffractograms were processed using DPDAK v150,^49^ and the background correction was done in Python using the arPLS method.^50^

#### Computational Details

All computations were performed using the CASTEP code.^51^ Cell files were generated using a Python script implemented in CSD Mercury software based on the structures deposited in the CSD (TP refcode: BAPLOT01,^52^ TP-MH refcode: THEOPH06,^53^ BZ refcode: BZAMID01,^54^ TP:BZ F1m refcode: RABXIE02,^44^ TP:BZ F2s refcode: RABXIE01,^8^ MNZ refcode: MNIMET,^55^ GAL refcode: IJUMEG05,^56^ GAL-MH refcode: KONTIQ01,^57^ MNZ:GAL F1s refcode: VOKYEC,^45^ MNZ:GAL F2m refcode: VOKYEC01 ^46^). Geometry optimization was performed using the Perdew–Burke–Ernzerhof (PBE) generalized gradient approximation (GGA) exchange correlation density functional^58^ and ultrasoft pseudopotentials^59^ with the addition of the Tkatchenko and Scheffler (TS) dispersion model.^60^ The Monkhorst–Pack grid was sampled with 0.05 Å^–1^ separation of *k*-points and a cutoff energy of 800 eV, both optimized for convergence. Geometry optimization was done with constrained cell dimensions. Chemical shifts were calculated using the gauge including projector augmented wave approach (GIPAW)^61,62^ as implemented in the CASTEP code. To convert the generated isotropic shielding constants (σ_calc_) to chemical shifts (δ_calc_), the following equation was used: δ_calc_ = σ_ref_ – σ_calc_. As the reference shielding constant value (σ_ref_) the average of calculated shieldings and experimental chemical shifts (σ_ref_ = ⟨ σ_calc_⟩ + ⟨δ_exp_ ⟩) was used.^63^ Because methyl groups undergo rapid rotational averaging, the three equivalent protons of each CH_3_ moiety were treated as a single entity, yielding one calculated shielding value per methyl group.^64^ When multiple calculated shieldings were assigned to a single observed peak, a weight of 1/*m* in the calculation of these averages was applied, where *m* is the number of calculated shielding values. The σ_*ref*_ values were established jointly for each set of related compounds, i.e., subsets of (i) ^1^H NMR TP:BZ, (ii) ^1^H NMR MNZ:GAL, (iii) ^13^C NMR TP:BZ, and (iv) ^13^C NMR MNZ:GAL.

#### Solubility Determination

The solubility of TP, BZ, MNZ, and GAL in the solvents selected for the NMR studies (H_2_O, MeOH, TOL) was determined. Suspensions of the neat compounds or cocrystals, i.e., TP:BZ F2s, MNZ:GAL F1s (200 mg, 200–700 μL) were prepared in glass vials, sealed with caps to prevent solvent evaporation, and then stirred at RT using a magnetic stirrer (300 rpm). After 24 h (if not stated otherwise), the solvents were collected with an automatic pipet, filtered through a hydrophobic 0.22 μm PTFE membrane filter, diluted, and subjected to HPLC analysis. The remaining powders were tested using PXRD to ensure the absence of solvent-mediated phase transitions. All of the solubility terms used in this work were taken from the United States Pharmacopeia.^65^

#### High-Performance Liquid Chromatography (HPLC)

HPLC analysis was performed on an Agilent 1260 Infinity system equipped with a Supelco Ascentis Express column (C18, 100 mm × 4.6 mm, 2.7 μm). The analysis used a 10 μL sample injection volume and water (with 0.1% v/v formic acid) (A) and acetonitrile (B) as a mobile phase. For TP (RT: 2.17 min) and BZ (RT: 3.30 min), a gradient elution (B: 12→98%, 0–4.5 min) with a flow rate of 0.7 mL min^–1^ was used. The column temperature was maintained at 30 °C, and the detection wavelength was set at 245 nm. MNZ (RT: 3.28 min) and GAL (RT: 1.30 min) analysis was performed using a gradient elution (MNZ - B: 2→70%, 0–3.5 min; GAL - B: 2→98%, 1–3.5 min), a flow rate of 0.8 mL min^–1^, the column temperature of 30 °C and the detection wavelength of 305 nm.

#### Permeability Measurements

The neat components (TP, BZ) and their mixture (TP:BZ PM, 1:1 molar ratio) were placed in the custom 3D-printed holders.^66^ The powders were then pressed to ensure an even, compacted surface. The permeability of a water drop through the powders was measured using an Ossila Contact Angle Goniometer. A short film was recorded during each measurement using a high-resolution camera set to 30 FPS and analyzed using the provided software.

## Results

### Systems under Investigation

Polymorphs of theophylline and benzamide (TP:BZ) cocrystal have been described extensively in the literature, including their structure and bonding motifs,^8,44,67^ cocrystallization mechanism,^8,20^ and the effect of solvent polarity on the polymorphic outcome in LAG experiments.^8,10^ The metastable form I (TP:BZ F1m, *Z′* = 2) is assembled during NG or LAG with nonpolar solvents, whereas the thermodynamically stable form II (TP:BZ F2s, *Z′* = 1) cocrystallizes in the presence of polar solvents. TRIS-PXRD observations of the ball milling indicated that the stable polymorph TP:BZ F2s is formed in a two-step mechanism, and TP:BZ F1m is observed as an intermediate in the cocrystallization process. The individual components, theophylline and benzamide, also exhibit polymorphism, and in addition to that, theophylline can crystallize in a hydrated form (TP-MH).^68–72^ In a recent study, Lampronti et al. showed that high-resolution TRIS-PXRD measurements of the ball milling with water, as the polar solvent, not only led to the crystallization of TP:BZ F2s with the transient formation of TP:BZ F1m, but also indicated the appearance of TP-MH in the very beginning of the process, which was previously unobservable.^8,20^

Based on these features, i.e., known polymorphism of a cocrystal and one of its individual components presenting a hydrated form, the second system of metronidazole and gallic acid was selected. It has two polymorphic forms, i.e., the metastable form II (MNZ:GAL F2m, *Z′* = 1) and the thermodynamically stable form I (MNZ:GAL F1s, *Z′* = 1).^45,46^ Among the cocrystallization techniques of the MNZ:GAL system, solution-based methods (including electrospraying or spray drying),^45,46,73^ thermal inkjet printing^46^ and LAG^46^ have been reported. However, LAG synthesis was carried out exclusively in the presence of methanol. While the effect of solvent properties on the polymorphic outcome has been studied using solvent-mediated crystallization,^73^ no systematic study using the ball milling experiments has been reported. Therefore, we performed a polymorphic screening with a set of solvents differing in polarity, confirming that the MNZ:GAL system is governed by the same motif as the TP:BZ cocrystal: the high-polarity solvents (*E*_*T*_^*N*^ in a range from 1.000 to 0.460)^74^ resulted in the pure MNZ:GAL F1s, whereas the low-polarity solvents (*E*_*T*_^*N*^ in a range from 0.117 to 0.006) yielded the mixture of MNZ:GAL F2m and unreacted starting materials (MNZ:GAL PM) (Figure S2). Dyba et al., based on the outcome of stirred suspension crystallization, determined that the thermodynamically stable form MNZ:GAL F1s is observed when the relative polarity value is above 0.35.^73^ This value falls into our intermediate zone (*E*_*T*_^*N*^ in a range from 0.386 to 0.228), where we observed no distinct pattern. The lack of a sharp transition point in LAG, in contrast to stirred suspension crystallization, could be due to the experimental design of both methods. During stirred suspension crystallization, the samples were periodically withdrawn and analyzed. The transition time varied from 3 days to 2 weeks, depending on the solvent, whereas in the ball milling experiments, all parameters (including the milling time) were kept constant.

Apart from polymorphism control, the stirred suspension crystallization results reveal the cocrystallization mechanism as MNZ:GAL F2m formed initially and then transformed into MNZ:GAL F1s.^73^ The same order of appearing polymorphs was observed in grinding. We confirmed this using TRIS-PXRD studies, which showed MNZ:GAL F2m formation prior to the occurrence of MNZ:GAL F1s (Figure 1). Worth noting is the polymorphic behavior of the starting materials. Metronidazole does not exhibit polymorphism, while gallic acid is known to have five anhydrate polymorphs and three polymorphic forms of a monohydrate (GAL-MH).^56^ As predicted, based on the TRIS-PXRD data of the TP:BZ system, in the process of MNZ:GAL cocrystal formation, the metastable polymorph is not the only transient product, and the appearance of GAL-MH is observed. In fact, TRIS-PXRD indicated only minor traces of neat GAL (11.64 nm^–1^), which converted immediately into the hydrated form of GAL (11.53 nm^–1^) (Figure 1). In Stage 1 (0–5 min), in addition to GAL-MH (5.83, 11.53, 13.55 nm^–1^) and MNZ (9.82, 12.76 nm^–1^) as starting materials, the instant formation of MNZ:GAL F2m was also noted (10.36, 10.80, 11.25, 13.88, 18.23, 19.77 nm^–1^). During Stage 2 (5–7.5 min), no traces of GAL-MH and MNZ were detected. However, MNZ:GAL F2m was still observable, and at the same time, the assembly of MNZ:GAL F1s occurred. In Stage 3 (8–60 min), the MNZ:GAL F1s polymorph remained the only product.

**Figure 1:**
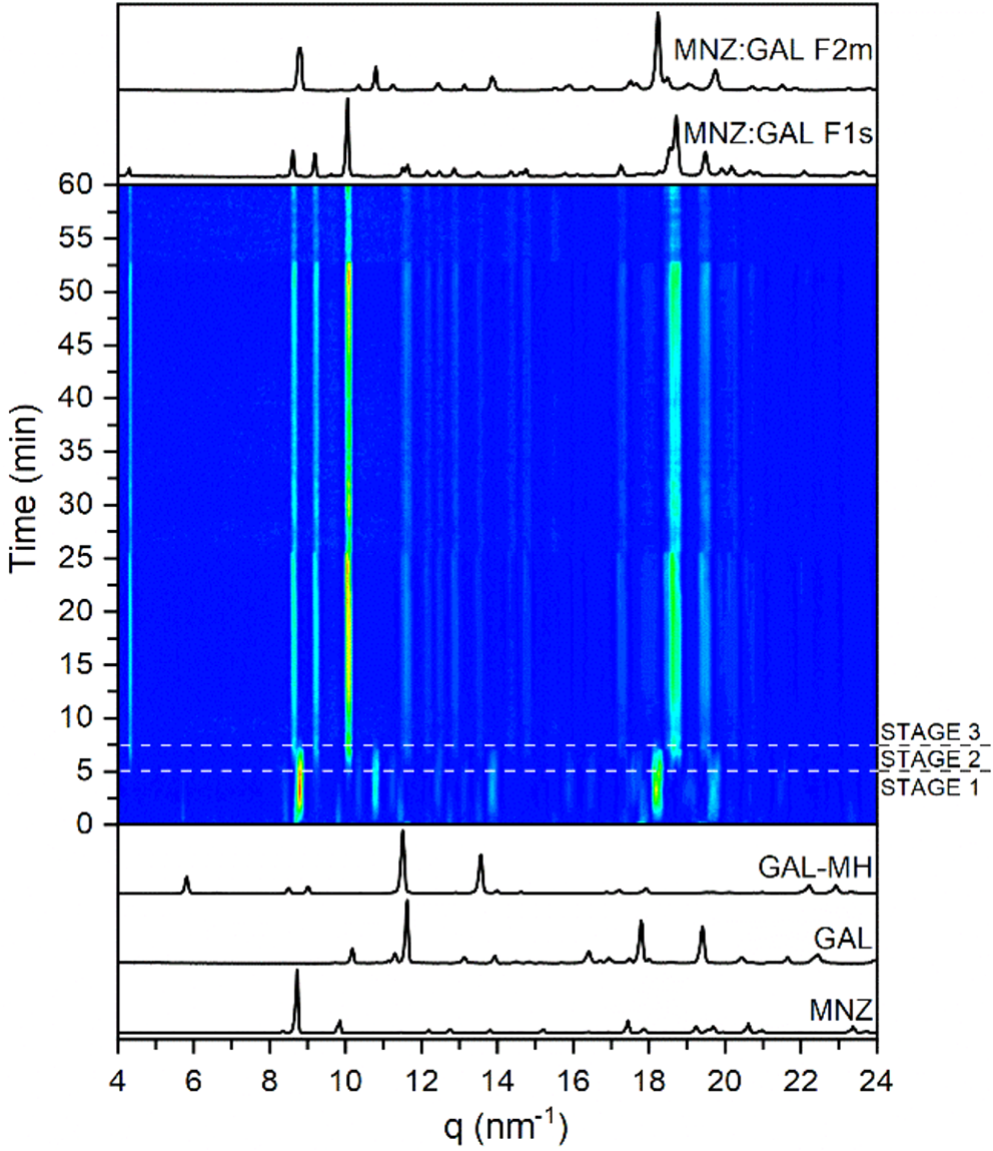
TRIS-PXRD measurement during LAG of MNZ:GAL with water (150 mg of powder, 25 μL of water, η = 0.17 μL mg^–1^, RT, 30 Hz, 60 min).

On the basis of polymorphic behavior and solvate formation patterns of the TP:BZ and MNZ:GAL systems, three solvents of different polarity, namely, toluene (low-polarity, no solvate formation), methanol (high-polarity, no solvate formation), and water (high-polarity, hydrate formation), were selected for further *in situ* NMR monitoring using the CLASSIC NMR approach.

### Experiment Design

*In situ* NMR studies of the cocrystallization mechanism are facilitated due to the mechanical force of the centrifugal pressure acting on the powders inside the spinning NMR rotor (Figure 2A). The pressure inside the rotor has been estimated to be ca. 6.5 MPa at the inner wall of the 4 mm rotor when spun at 12.5 kHz MAS frequency.^25,75^ This is much lower than the impulses of pressure inside the jar of a ball mill (calculated to be in the order of several GPa).^24,76^ However, it is sufficient to induce the mechanochemical reaction. Concurrently, the reaction inside the rotor proceeds at a slower rate compared to the ball mill,^25^ which enables step-by-step monitoring of the cocrystallization mechanism.

**Figure 2:**
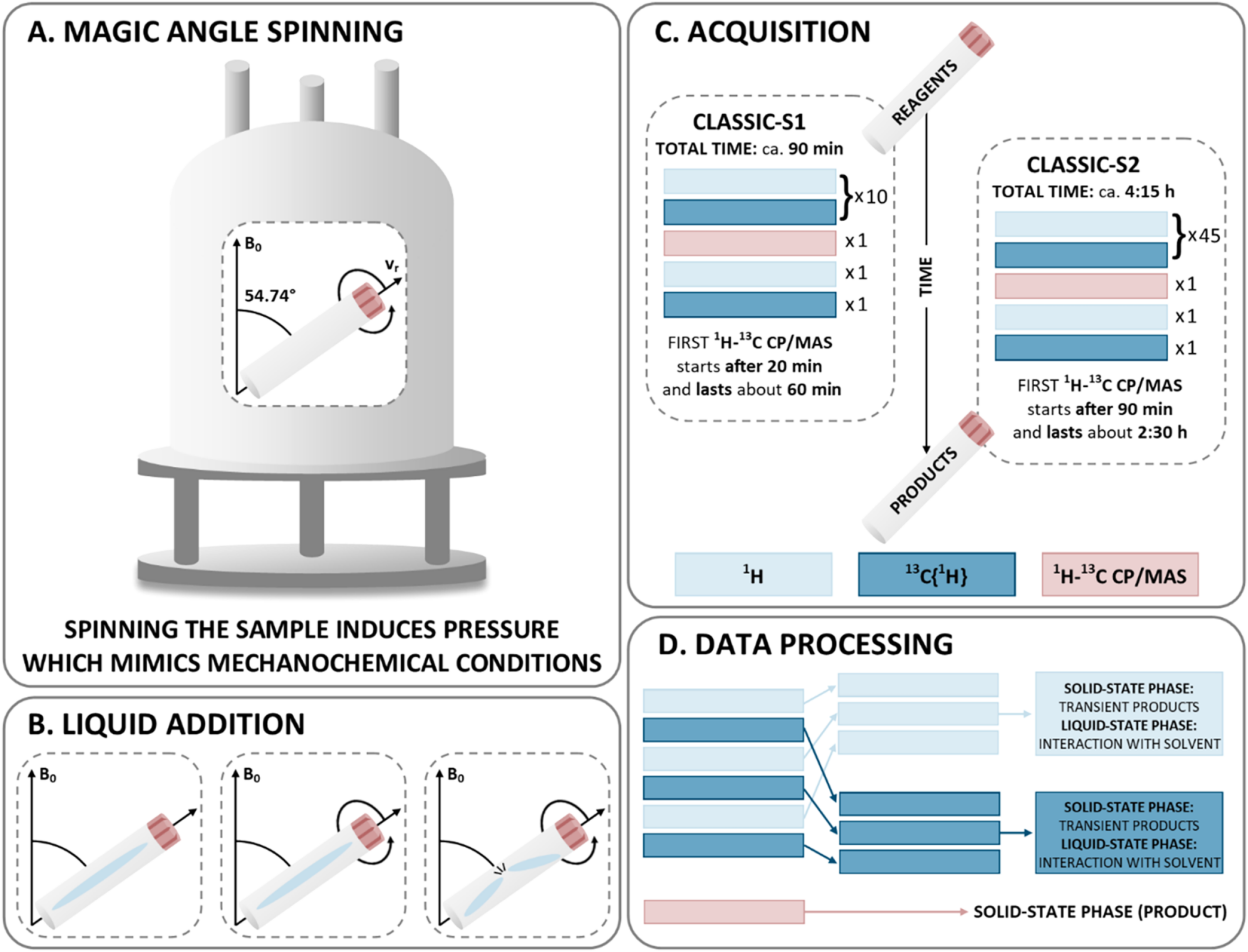
Design of CLASSIC NMR experiments: (A) A sample placing in the spectrometer and mechanochemical reactions conducted under the MAS (magic-angle spinning) conditions. (B) Addition of a liquid to a sample using a glass capillary to mimic the liquid-assisted grinding (LAG) experiment. (C) The order and timeline of the ^1^H NMR, ^13^C­{^1^H} NMR, and ^1^H–^13^C CP/MAS NMR experiments in CLASSIC-S1 and CLASSIC-S2 sequences. (D) Processing of the obtained data and information acquired from phase changes observed in time in ^1^H, ^13^C­{^1^H}, and ^1^H–^13^C CP/MAS NMR spectra.

The starting material for all of the experiments conducted in the study was a physical mixture of either TP and BZ (TP:BZ PM) or MNZ and GAL (MNZ:GAL PM). To avoid incomplete cocrystallization due to limited mixing inside the rotor, the powders were premixed using a benchtop vortexer to ensure the uniform distribution of the starting materials prior to subjecting the sample to MAS conditions. This setup would mimic the neat grinding (NG) experiment in a ball mill, when no additives are present during grinding. To mimic the liquid-assisted grinding (LAG) method, a solvent of choice was added to the system. To separate the powder from the solvent before the start of data acquisition, the liquid was sealed in a glass capillary (Figure 2B). The capillary was positioned concentrically inside the rotor, and the powder was evenly distributed around the capillary. Upon spinning of the rotor, the capillary broke, releasing the solvent. This allowed the start of *in situ* NMR data acquisition to be synchronized with the initial contact between the powder and the solvent. Otherwise, if the solvent was added directly to the powders during sample preparation, early phases of the cocrystallization process (even up to 30 min) could be overlooked, which was described by Xu et al. in their work.^30^ Deuterated solvents of increasing polarity: toluene-*d*_8_ (TOL_d_), methanol-*d*_4_ (MeOD), and deuterium oxide (D_2_O) were used to suppress solvent peaks in ^1^H NMR experiments. The ratio of solvent volume to sample weight (η)^12^ was either 0.14 μL mg^–1^ (one capillary) or 0.28 μL mg^–1^ (two capillaries) while the amount of powder was kept constant (70 mg), which is the typical ratio for LAG experiments, including when TRIS-PXRD is used for reaction monitoring.^12,20^ The phase transitions that occur in TP:BZ PM or MNZ:GAL PM when subjected to MAS conditions were followed based on changes of chemical shifts in the ^1^H, ^13^C­{^1^H}, and ^1^H–^13^C CP/MAS NMR spectra (Figure 4 and Figure 5).

The NMR studies have been designed based on the CLASSIC NMR sequence and adjusted to the nature of the investigated compounds and the rate of the expected phase transformations. The original CLASSIC NMR^34^ experiments consisted of alternating ^1^H MAS, ^13^C MAS, and ^1^H–^13^C CP/MAS NMR acquisitions to monitor changes in both liquid- (^13^C NMR) and solid-state (^1^H NMR, ^1^H–^13^C CP/MAS NMR). This is due to the cross-polarization (^1^H–^13^C CP/MAS NMR) being effective for rigid solids only, and direct-excitation measurements (^13^C NMR) detecting liquid phase selectively when a sufficiently short recycle delay is used.^34^ The CLASSIC NMR approach was originally applied to study crystallization of *m*-aminobenzoic acid from the saturated solution^34^ and to observe the precipitation of glycine crystals and further transformation from α to γ polymorph.^35,77^

The materials in our study have relatively long ^1^H spin–lattice relaxation times, e.g., benzamide requires up to 400 s of the recycle delay^69^ and theophylline ca. 130 s^78^ during ^1^H–^13^C CP/MAS NMR. This results in long times required to record a ^1^H–^13^C CP/MAS NMR spectrum with an acceptable signal-to-noise (S/N) ratio, to distinguish the respective phases in the reaction mixture. We expected rapid changes during the early stages of the experiment, which could not be followed with time-consuming ^1^H–^13^C CP/MAS NMR acquisitions. Instead, to provide an acceptable time resolution, we recorded a single ^1^H–^13^C CP/MAS NMR spectrum at the end of an experimental sequence, after a series of ^1^H NMR and ^13^C NMR acquisitions, which are significantly shorter measurements compared to ^1^H–^13^C CP/MAS NMR. Additionally, for the direct detection of ^13^C, we decided to implement the ^13^C­{^1^H} pulse sequence (with high-power ^1^H decoupling) to improve the S/N ratio and simplify the spectra. Consequently, two protocols differing in the number of alternating ^1^H NMR and ^13^C­{^1^H} NMR experiments were established: the shorter CLASSIC-S1 (^1^H NMR and ^13^C­{^1^H} NMR repeated 10 times) and the longer CLASSIC-S2 (^1^H NMR and ^13^C­{^1^H} NMR repeated 45 times), which enabled us to gain insight into the mechanism of the reaction and the transient products appearing at different time points (Figure 2C). When needed, the rotor was kept spinning, while the additional ^1^H–^13^C CP/MAS NMR spectra were recorded to extend the sequence.

The distinction between ^13^C (or ^13^C­{^1^H}) MAS NMR and ^1^H MAS NMR spectra being susceptible to changes in liquid- and solid-state, respectively, as designated in the original CLASSIC, is not that evident for our systems. In ^13^C­{^1^H} MAS NMR experiments, the relaxation delay was kept short to exclude peaks arising from neat powders and exclusively probe the liquid-state part of the sample, i.e., any components dissolving in a solvent of choice or solvent signals (Figure 3B). However, it is important to note that in both systems studied (TP:BZ and MNZ:GAL), there are methyl groups in the structure of APIs (TP, MNZ). These show short spin–lattice relaxation times. Therefore, despite being in a solid-state phase, those moieties are detectable in the ^13^C­{^1^H} NMR spectra recorded with short relaxation times (Figure 4D and Figure 5D). The position of the ^13^C peaks assigned to methyl groups in both TP:BZ (C6, C7) and MNZ:GAL (C4) systems is indicative of the solid form of the API, i.e., neat APIs, their hydrated forms, and cocrystal polymorphs can be differentiated based on those signals (Figure 4F and Figure 5F). When considering ^1^H NMR spectra, the proton lines of TP:BZ (H1) and MNZ:GAL (H10) allow for the monitoring of phase transitions in the solid-state during cocrystallization (Figure 4C and Figure 5C). However, the ^1^H NMR spectra might also be affected by the interaction of the components with the added solvent, e.g., even partial dissolution of the compound increases the mobility of a system, resulting in changes in the position and shape of the NMR signals. This is mostly reflected in the narrower lines, contrary to those for undissolved neat powders. However, the interpretation of the recorded ^1^H NMR data might pose a challenge as the spectra could present overlapping peaks of both dissolved and undissolved species (Figure 3A). For these reasons, we used the ^1^H–^13^C CP/MAS NMR experiment to get the information about the solid-state part of the reaction mixture selectively as CP (cross-polarization) requires the presence of a rigid structure providing a strong network of ^1^H–^13^C dipolar couplings.^36^

**Figure 3:**
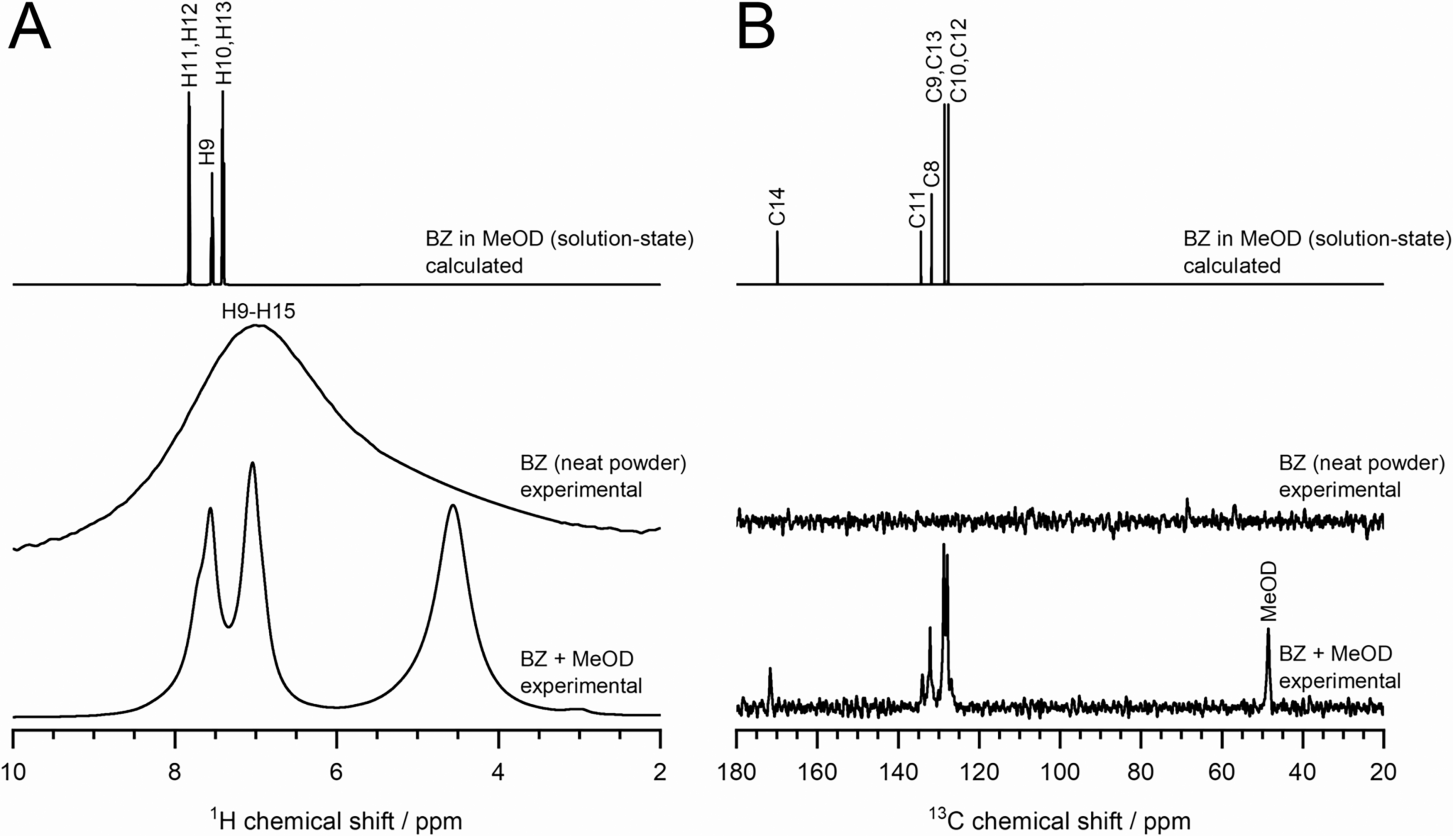
(A) ^1^H and (B) ^13^C­{^1^H} MAS NMR spectra of the BZ (neat powder) and BZ with MeOD delivered via sealed glass capillary (dissolved)^80^ recorded using solid-state NMR setup. These experimental data were compared with the calculated ^1^H and ^13^C solution-state NMR spectra of BZ, which were generated using MNova (MestreLab) software (solvent: MeOD; ^1^H: labile protons excluded, ^13^C: proton decoupled).

**Figure 4:**
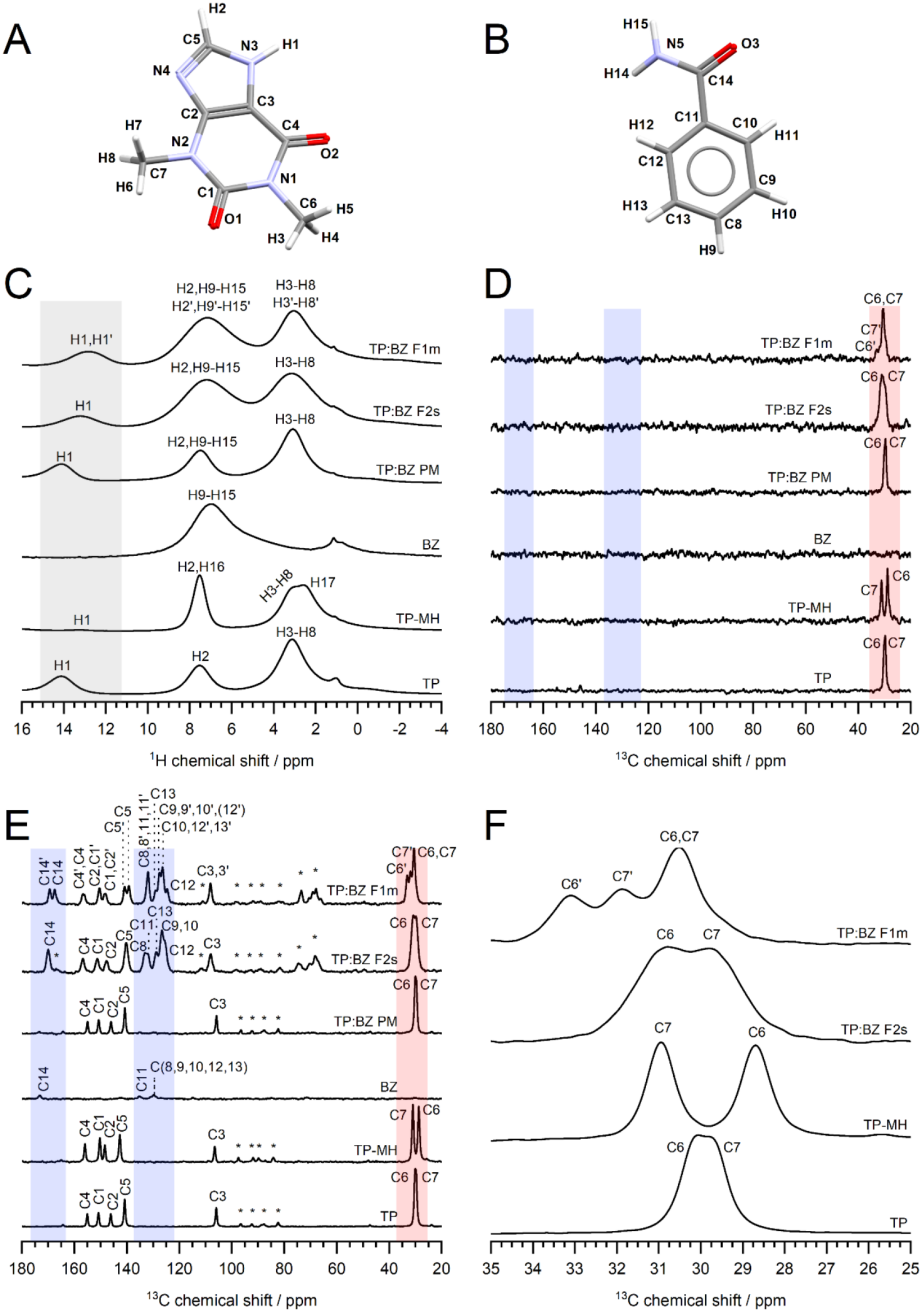
Molecular structures of (A) TP and (B) BZ with atom labeling, (C) ^1^H NMR (a peak at ca. 1 ppm was assigned to the background signal of the NMR probe), (D) ^13^C­{^1^H} NMR (d_1_ = 3 s), (E) ^1^H–^13^C CP/MAS NMR spectra of TP, TP-MH, BZ, TP:BZ PM, TP:BZ F1m, and TP:BZ F2s, (F) the peaks assigned to methyl groups of TP in ^13^C NMR spectra (35–25 ppm). The spinning sidebands are labeled with asterisks. The spectral regions assigned to the H1 site, the methyl group of TP, and the region distinct to BZ are marked with gray, red, and blue rectangles, respectively. The brackets provide alternative assignments (for computational details, see Table S1 and Table S2 in ESI).

**Figure 5:**
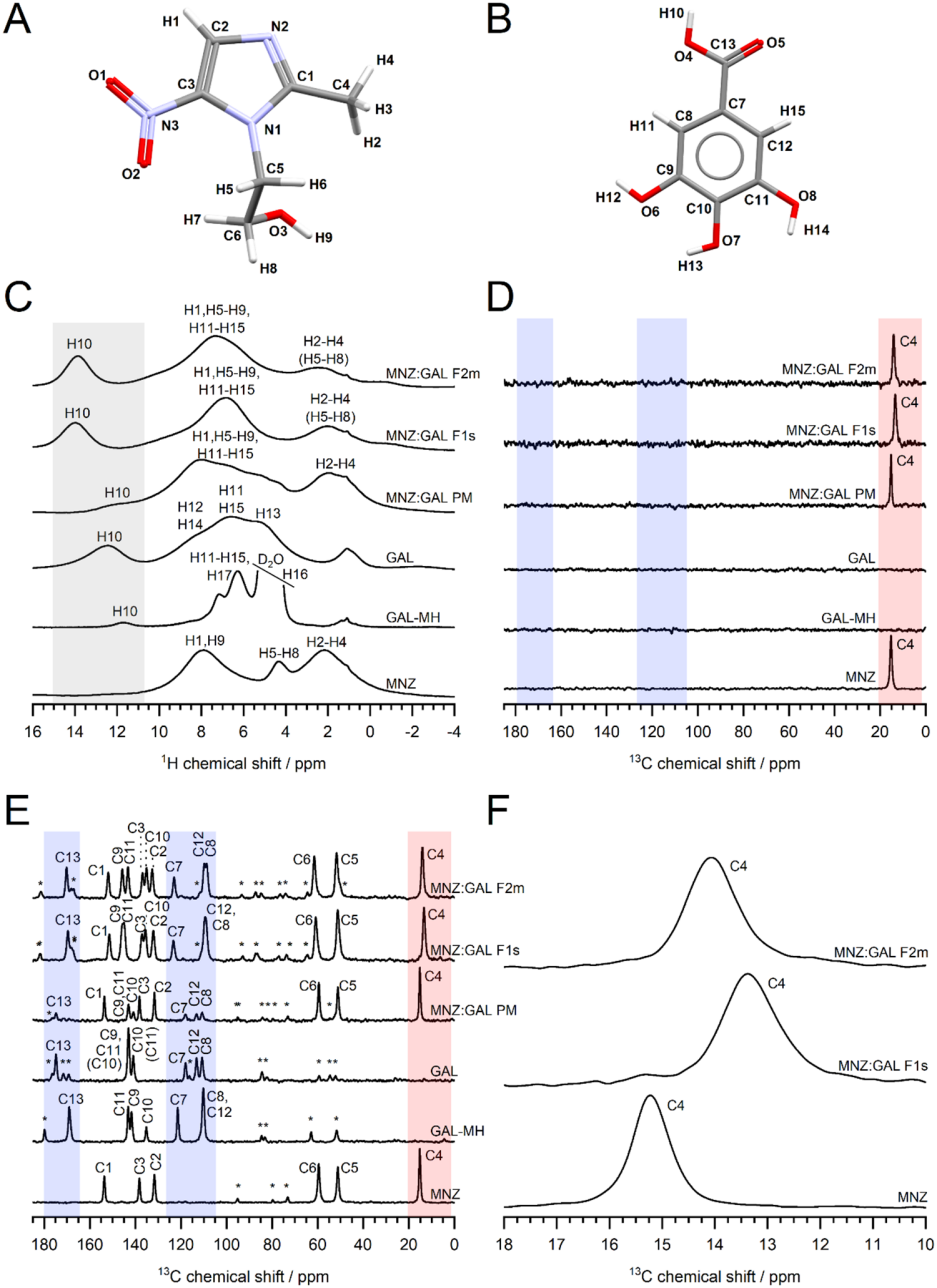
Molecular structures of (A) MNZ and (B) GAL with atom labeling, (C) ^1^H NMR (a signal at ca. 1 ppm was assigned to the background signal of the NMR probe; a signal arising from the residual water in GAL-MH was cropped), (D) ^13^C­{^1^H} NMR (d_1_ = 3 s), (E) ^1^H–^13^C CP/MAS NMR spectra of MNZ, GAL, GAL-MH, MNZ:GAL PM, MNZ:GAL F1s, and MNZ:GAL F2m, (F) the peaks assigned to the methyl group of MNZ in ^13^C NMR spectra (10–18 ppm). The spinning sidebands are labeled with asterisks. The spectral regions assigned to the H10 site, the methyl group of MNZ, and the region distinct to GAL are marked with gray, red, and blue rectangles, respectively. The brackets provide alternative assignments (for computational details, see Table S3 and Table S4 in ESI).

As the final step, the ^1^H and ^13^C­{^1^H} NMR results were analyzed as time-resolved maps, while ^1^H–^13^C CP/MAS NMR experiments were used to confirm phase identity by comparison with the spectra of the reference materials (Figure 2D). The ^1^H–^13^C CP/MAS NMR measurements were relatively long, with total acquisition times of 60 min for CLASSIC-S1 and 2.5 h for CLASSIC-S2 (Figure 2C). Therefore, in the analysis of the ^1^H–^13^C CP/MAS NMR data, the start and end times of each acquisition are denoted in brackets, e.g., CLASSIC-S1 (20–80 min). Unlike CLASSIC NMR and SASSY NMR, in our experiments, the data were collected at a constant temperature of ca. 35 °C, including frictional heating produced by MAS.^79^

### CLASSIC NMR Studies of TP:BZ

### Formation of a Hydrate, Component Dissolution, Cocrystal Precipitation, and Polymorphic Transition

For TP:BZ, the CLASSIC NMR studies were conducted using three solvents, namely, D_2_O, MeOD, and TOL_d_. The ratio of solvent volume in the capillary (μL) to the TP:BZ mixture (mg, 1:1 molar ratio) was kept constant at 0.14 μL mg^–1^. The data for TP:BZ D_2_O are presented in Figure 6 as a model data set, while those for TP:BZ MeOD and TP:BZ TOL_d_ can be found in the ESI (Figure S13 and Figure S14, respectively).

**Figure 6:**
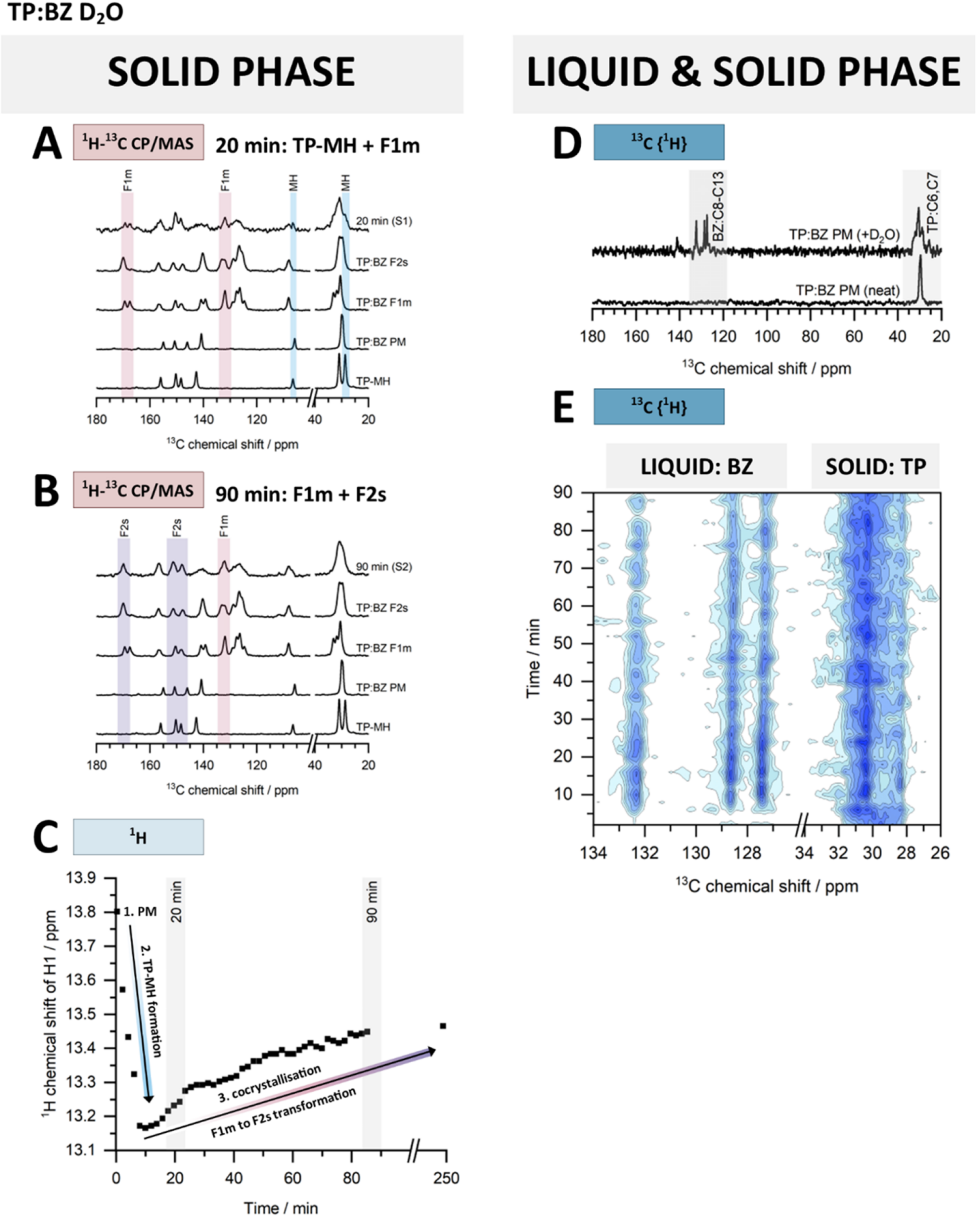
^1^H–^13^C CP/MAS NMR spectra of TP:BZ D_2_O sample (A) 20–80 min and (B) 90–250 min into CLASSIC NMR experiments compared with the reference spectra (TP-MH is marked with light blue rectangles, TP:BZ F1m with pink rectangles and TP:BZ F2s with purple rectangles). (C) Changes in the ^1^H NMR chemical shift (ppm) of the H1 proton of the TP:BZ system monitored during the CLASSIC NMR experiment conducted with the addition of D_2_O; for details, see Figure S20. (D) Comparison of the ^13^C­{^1^H} NMR spectra of neat TP:BZ PM and after the addition of D_2_O (regions changing are marked with gray rectangles). (E) Intensity contour plot comprising all ^13^C­{^1^H} NMR spectra recorded as a function of time (135–124 ppm region, d_1_ = 3 s).

Based on the TRIS-PXRD data reported by Lampronti et al.,^20^ when water is added during grinding, the cocrystallization of TP:BZ F2s is preceded by the conversion of TP into TP-MH, with no detectable traces of the transient TP:BZ F1m form. In contrast, Fischer et al.^8^ demonstrated that during LAG with MeOH, TP:BZ F1m forms prior to the assembly of the TP:BZ F2s polymorph. In this regard, we hypothesized that the mechanism of TP:BZ cocrystallization in the presence of water proceeds as follows: (i) TP converts to TP-MH prior to (ii) cocrystallization of TP:BZ F1m, which consequently (iii) transforms to TP:BZ F2s. Spectroscopic data, collected using CLASSIC-S1 and CLASSIC-S2 sequences, support this mechanism, confirming the initial formation of TP-MH followed by the subsequent assembly of TP:BZ F1m. The latter step, i.e., cocrystallization of TP:BZ F1m, may have been overlooked in TRIS-PXRD studies due to the rapid reaction rate and the short-lived nature of this intermediate. In such cases, *in situ* monitoring could benefit from the slower reaction rate observed during CLASSIC NMR, allowing better resolution of transient phases. The conclusion is supported by both ^1^H–^13^C CP/MAS NMR and ^1^H NMR data analysis. In the first ^1^H–^13^C CP/MAS NMR spectrum (CLASSIC-S1, 20–80 min), no residual TP was detected, confirming that the conversion of TP to TP-MH was complete before the 20 min mark. In addition, traces of TP-MH remained visible in the spectrum together with the peaks corresponding to the TP:BZ F1m cocrystal polymorph (Figure 6A), while no signals attributable to TP:BZ F2s were observed. The second ^1^H–^13^C CP/MAS NMR experiment (CLASSIC-S2, 90–250 min) shows no peaks originating from TP or TP-MH (Figure 6B), indicating that all of the starting materials have already been consumed to assemble the TP:BZ cocrystal. At this stage, the relative peak intensities of TP:BZ F1m and TP:BZ F2s reveal an almost complete transformation of TP:BZ F1m into TP:BZ F2s. Nonetheless, ^1^H–^13^C CP/MAS NMR experiments are time-consuming; therefore, they were performed at the end of each experimental sequence to confirm the phase identity. Prior to these measurements, a series of ^1^H acquisitions was collected to assess whether they could yield equivalent information with superior time resolution (i.e., a single ^1^H NMR and ^1^H–^13^C CP/MAS NMR experiments require ca. 20 s and 60 min, respectively). The analysis was based on the H1 peak position in ^1^H NMR, which is distinct for TP (14.1 ppm), TP-MH (13.2 ppm), TP:BZ F1m (12.8 ppm), and TP:BZ F2s (13.2 ppm) (Figure 4C and Table S2). The H1 peak at 13.8 ppm gradually shifts to lower frequency, reaching 13.15 ppm after 10 min (Figure 6C and Figure S20), consistent with the transformation of neat TP into TP-MH. The initial H1 position at 13.8 ppm (vs 14.1 ppm in the neat material) can be due to the immediate start of TP-MH formation upon contact with D_2_O, thus the first recorded spectrum shows a mixture of neat TP and TP-MH. After reaching 13.2 ppm, the H1 peak shifts toward higher frequency (13.4 ppm). Although this behavior follows the expected trend, the observed values do not match the exact reference shifts of TP:BZ F1m and TP:BZ F2s. This discrepancy likely arises from the coexistence of several phases (TP-MH, TP:BZ F1m, and TP:BZ F2s), whose overlapping resonances hinder their differentiation. Therefore, while ^1^H NMR effectively monitors rapid transformations such as the TP to TP-MH conversion and provides valuable kinetic insight, it should be interpreted in conjunction with ^1^H–^13^C CP/MAS NMR data for comprehensive phase identification.

These observations, revealed by both TRIS-PXRD^20^ and CLASSIC NMR, reflect changes occurring only within the ordered solid-state phases. However, NMR measurements can be tailored to probe not only the solid state but also the liquid phase (i.e., ^13^C­{^1^H} NMR with a short recycle delay), which is particularly valuable for investigating the role of the solvent in LAG processes. A series of the ^13^C­{^1^H} NMR spectra recorded during the TP:BZ D_2_O experiment (Figure 6D) were plotted to illustrate time-resolved changes occurring in the liquid phase (Figure 6E). Virtually no signal was detected in the ^13^C­{^1^H} liquid phase spectra (134–126 ppm) during the first 5 min. Bearing in mind a significant shift of the H1 ^1^H NMR peak within the same initial period of the CLASSIC NMR experiment (Figure 6C), this likely reflects that at the beginning of the reaction, water is primarily engaged in TP-MH formation. After 5 min, ^13^C­{^1^H} NMR peaks assigned to dissolved BZ appeared, reaching a maximum intensity between 10 and 15 min. Consequently, the signal intensity decreased as BZ was leaving the liquid phase, forming the TP:BZ cocrystal. No lines in the spectral regions characteristic of TP or TP-MH (except for the mobile CH_3_ groups) were detected in any of the ^13^C­{^1^H} NMR spectra, indicating that TP does not enter the liquid phase during the reaction. The ^13^C­{^1^H} NMR experiments recorded with a short recycle delay are also sensitive to the solid-state form of TP (Figure 6D). In the spectral region of 34–26 ppm, the peak produced by neat TP is quite narrow but broadens significantly after the addition of D_2_O and TP to TP-MH conversion (Figure S8E). In the TP:BZ D_2_O experiment, this broadening is observed from the beginning of the CLASSIC NMR sequence, consistent with the TP to TP-MH transformation, which aligns with the ^1^H NMR data indicating rapid TP-MH formation in the early stages of the reaction (Figure 6C). Further broadening of the TP CH_3_ resonance was expected once the TP:BZ cocrystal assembled due to the downfield chemical shift of the C6′ and C7′ atoms (TP:BZ F1m) (Figure 4F). This was indeed observed in the TP:BZ D_2_O sample at ca. the 10 min mark (Figure 6E). Further distinction between the TP:BZ F1m and TP:BZ F2s polymorphs was not possible based on the ^13^C­{^1^H} NMR data.

### The Change of Solvent Results in the Slower Cocrystallization

In the TP:BZ MeOD system, the first ^1^H–^13^C CP/MAS NMR spectrum (Figure S13B, CLASSIC-S1, 20–80 min) revealed peaks corresponding to neat TP (TP:BZ PM) and the TP:BZ F1m polymorph, with no evidence of TP:BZ F2s form. The second acquisition (CLASSIC-S2, 90–250 min) showed a mixture of all three phases. Compared to the corresponding TP:BZ D_2_O spectrum (i.e., 90–250 min), the phase distributions differed significantly. In TP:BZ D_2_O, no unreacted TP was detected, whereas in TP:BZ MeOD, some TP remained. Additionally, the C14 (and C14’) lines in the 172–165 ppm region allowed differentiation between TP:BZ F1m (*Z′* = 2, two peaks) and TP:BZ F2s (Z′ = 1, a single peak). Based on this spectral region, the TP:BZ D_2_O sample was nearly pure TP:BZ F2s, while TP:BZ MeOD remained a mixture of F1m and F2s. Later spectra (CLASSIC-S2, 740–900 min) were consistent with the pure TP:BZ F2s, revealing traces of TP:BZ F1m and residual TP. Although both D_2_O and MeOD ultimately yielded TP:BZ F2s, the cocrystallization rate in the presence of D_2_O was higher.

Further differences between the D_2_O and MeOD systems are reflected in the liquid phase behavior. In ^13^C­{^1^H} NMR spectra, peaks corresponding to dissolved BZ in MeOD (Figure S13C) were visible from the beginning of the experiment, whereas in D_2_O a ca. 5 min delay in BZ dissolution was observed (Figure 6E). Unlike in TP:BZ D_2_O, where water was incorporated into TP-MH, MeOD in TP:BZ MeOD did not form a solvate and remained available to BZ from the beginning. The signal intensity reached a maximum after 20 min and then gradually decreased, indicating the precipitation of BZ from the solution as the TP:BZ cocrystal forms. The ^1^H data analysis in the TP:BZ MeOD system further explored this matter. The precipitation of the cocrystal from the liquid phase can also be visualized by changes in the width (FWHM) of the ^1^H NMR peaks over time as the ^1^H lines produced by the liquid (dissolved) phase are significantly narrower than those from the solid phase. Changes in the phase distribution of BZ between the liquid and solid phases were monitored by the FWHM of the 7.5 ppm peak (Figure 3 and Figure S13D). From the beginning, this ^1^H peak was relatively narrow, confirming instant BZ dissolution. The ongoing dissolution was observed until the 20 min mark, as illustrated by the decreasing peak width. The subsequent increase in the width of the ^1^H BZ peak reflects the solidification of the system, i.e., cocrystallization and BZ precipitation, since the solid-state lines are broader than those from liquid-like species. This agrees with the mechanism proposed based on the ^13^C­{^1^H} NMR data analysis. Moreover, the comparable peak widths in ^1^H NMR at the start and after 40 min (Figure S13D) correspond to similar intensities in the ^13^C­{^1^H} NMR contour map at the respective time points (Figure S13C), further supporting the proposed mechanism.

In the last system investigated, i.e., TP:BZ TOL_d_, no dissolution of any component was revealed. Some traces of cocrystal assembly were detected, but the cocrystallization rate was decreased considerably compared to both D_2_O and MeOD samples. The details are provided in ESI (Figure S14).

### CLASSIC NMR Studies of MNZ:GAL

The MNZ:GAL samples were prepared and tested in accordance with TP:BZ. This involved using a solvent (D_2_O, MeOD, and TOL_d_) at a 0.14 μL mg^–1^ ratio (to the MNZ:GAL physical mixture). However, despite the similarities between the two systems, e.g., their solvent-induced polymorphic transition pathways (from metastable to stable form), hydrate formation (TP-MH or GAL-MH), or the presence of methyl groups in their structures (TP or MNZ), the analysis of the NMR data collected for the MNZ:GAL system posed some additional challenges. This is due to the structural similarity of the two MNZ:GAL cocrystal polymorphs and the strong resemblance of their NMR spectra. While differentiating between the phase-pure MNZ:GAL F2m and MNZ:GAL F1s does not present a problem upon the acquisition of the spectra with a high signal-to-noise ratio, (a) the experiment time during the CLASSIC NMR experiment is relatively short to ensure good time resolution and (b) both polymorphs could be present in a measured sample alongside the starting materials. These features affect the quality of the acquired spectra, which yield broad line and prevent accurate polymorph identification. Several regions in the ^1^H–^13^C CP/MAS NMR spectra can be used for the detection of MNZ:GAL cocrystal formation, e.g., 153–150, 147–144, 125–121 ppm (Figure 5E). The easiest way to illustrate this is to use the peak of the MNZ methyl group (17–11 ppm, C4), which is summarized in Figure 7.

**Figure 7:**
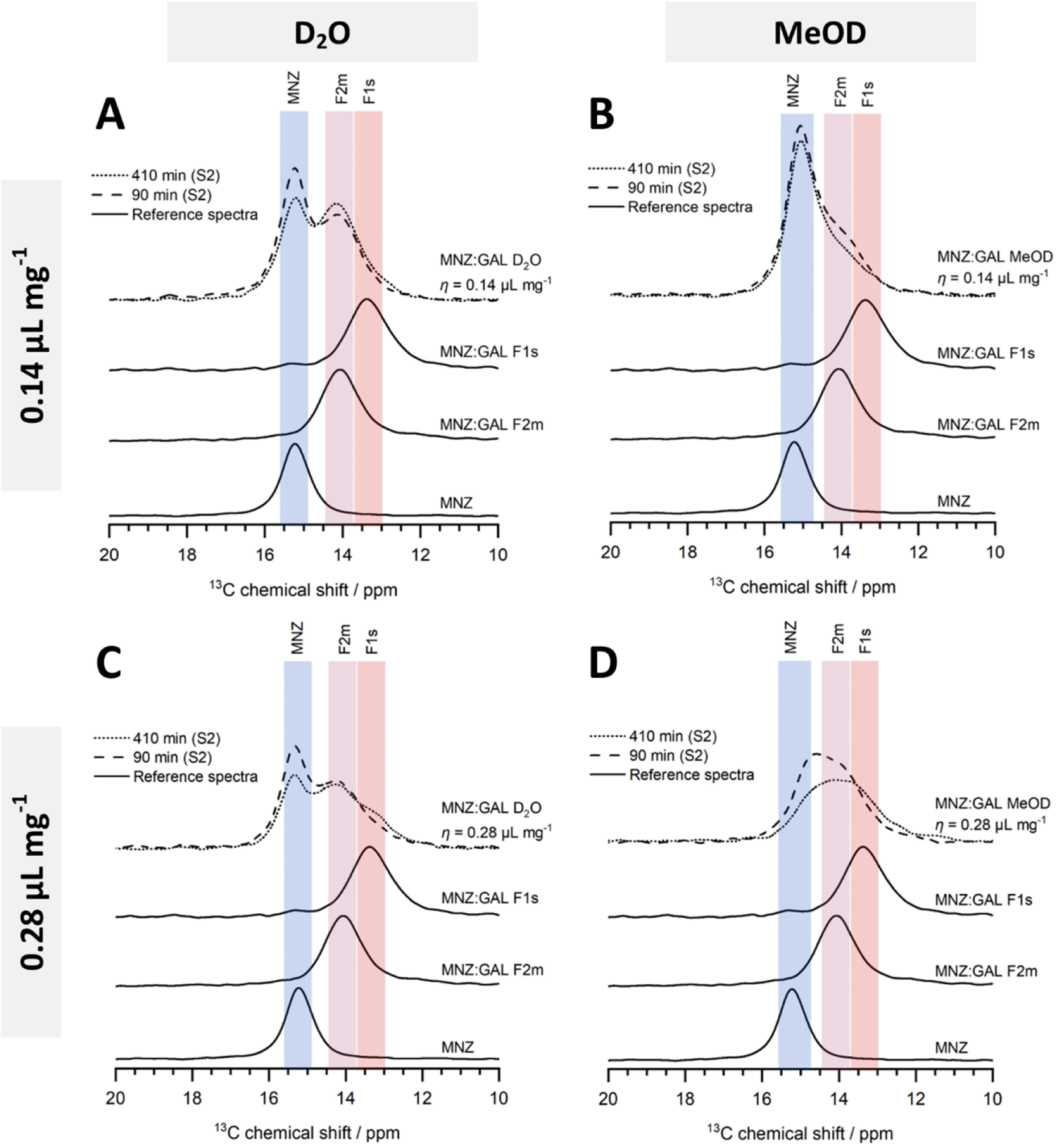
^1^H–^13^C CP/MAS NMR spectra recorded during the CLASSIC NMR of MNZ:GAL with (A) 1 capillary of D_2_O (0.14 μL mg^–1^), (B) 1 capillary of MeOD (0.14 μL mg^–1^), (C) 2 capillaries of D_2_O (0.28 μL mg^–1^), (D) 2 capillaries of MeOD (0.28 μL mg^–1^). The region presented (10–20 ppm) is assigned to the methyl group (C4) of MNZ. The spectra were acquired at different time points, i.e., 90 or 410 min (CLASSIC-S2 sequence) and compared with the reference spectra of MNZ (violet), MNZ:GAL F2m (cool pink), and MNZ:GAL F1s (warm pink).

### Different System, Similar Behavior

Regarding MNZ:GAL D_2_O (0.14 μL mg^–1^), the GAL-MH formation was expected, based on the TRIS-PXRD data (Figure 1), prior TP:BZ D_2_O sample observations, and CLASSIC NMR experiment of neat GAL with D_2_O (Figure S12A and Figure S12C). The ^1^H–^13^C CP/MAS NMR spectra confirmed its presence and revealed no residual traces of neat GAL after 20 min (CLASSIC-S1, 20–80 min), 90 min (CLASSIC-S2, 90–250 min), and 410 min (CLASSIC-S2, 410–570 min) (Figure S15A). This indicates full conversion of GAL into GAL-MH within the first 20 min of CLASSIC NMR acquisition. Simultaneously, gradual growth of the MNZ:GAL cocrystal (ca. 151, 145.5, and 123 ppm peaks) was visible in the ^1^H–^13^C CP/MAS NMR spectra. Subsequent review of the MNZ methyl group region (Figure 7A) demonstrated a predominant peak from the neat MNZ and the peak from concomitant MNZ:GAL F2m (90 min). Subsequently, the phase distribution changed, showing a decrease in unreacted MNZ and an increase in MNZ:GAL F2m (410 min) but with no indication of MNZ:GAL F1s formation. The ^1^H–^13^C CP/MAS NMR data on MNZ:GAL D_2_O were also supported by the ^13^C­{^1^H} NMR data revealing no dissolution (Figure S15E) which is due to the low water solubility of MNZ (Figure S6) and the fact that GAL transforms to GAL-MH instead of dissolving in water. This was further corroborated by ^1^H NMR analysis, and we focused on the peak of H10, similarly to the H1 peak in the TP:BZ system. The details are presented in ESI (Figure S16), but the results can be summarized as follows. The integrated intensities of the GAL-MH and MNZ:GAL cocrystal peaks showed that GAL-MH reached its maximum after 15 min, consistent with the ^1^H–^13^C CP/MAS NMR results, indicating its formation was complete before 20 min. Thereafter, the intensity of lines corresponding to GAL-MH decreased while the MNZ:GAL cocrystal peaks gradually increased, reflecting the consumption of GAL-MH during cocrystal formation.

In the next system investigated, MNZ:GAL MeOD (0.14 μL mg^–1^), neat GAL was detected (Figure S17A) as MeOD does not induce a phase transition of GAL (Figure S12B and Figure S12D). Similarly to MNZ:GAL D_2_O, the ^1^H–^13^C CP/MAS NMR acquisitions of the MNZ:GAL MeOD sample demonstrate occurring cocrystallization (Figure S17A), but the MNZ:GAL cocrystal peaks (ca. 151, 145.5, and 123 ppm) are far less pronounced than those observed in the D_2_O data set (Figure S15A). This is also reflected in the MNZ methyl group region of the ^1^H–^13^C CP/MAS NMR spectra: in the MeOD sample (Figure 7B), neat MNZ presents a signal of greater intensity than that of D_2_O (Figure 7A). At the same time, in MeOD, the peak assigned to MNZ:GAL F2m is visible only as a shoulder instead of the distinct peak observed for the D_2_O sample. This is due to a smaller amount of MNZ:GAL F2m being formed during the MNZ:GAL MeOD experiment compared to the MNZ:GAL D_2_O sample. The same is visible in the ^13^C­{^1^H} NMR spectra as the width of the methyl group peak is significantly broader for the MNZ:GAL D_2_O than for the MNZ:GAL MeOD sample (Figure S15C and Figure S17C). This therefore indicates that the MNZ:GAL cocrystallization process is more effective in the presence of D_2_O (higher polarity) than MeOD (lower polarity), as was also shown for the TP:BZ system.

### The Quantity of Solvent Matters—The More the Better?

Unlike in the TP:BZ system, neither of the two solvents produced a stable MNZ:GAL cocrystal polymorph (MNZ:GAL F1s). According to studies conducted by Fischer et al.^81^ and Friščić et al.,^12^ an increase in the amount of solvent added during the liquid-assisted grinding procedure can accelerate the cocrystal formation. Therefore, to address the lack of MNZ:GAL F1s cocrystallization, we decided to repeat the MNZ:GAL CLASSIC NMR experiments, but instead of placing 1 capillary (10 μL), 2 capillaries (20 μL) with a solvent of choice were inserted into a rotor. As the amount of MNZ:GAL physical mixture (MNZ:GAL PM) remained constant (70 mg), the solvent-to-powders ratio changed from 0.14 to 0.28 μL mg^–1^.

Evaluation of the ^1^H–^13^C CP/MAS NMR spectra of MNZ:GAL D_2_O (0.28 μL mg^–1^) confirmed the GAL to GAL-MH transition (with no traces of GAL residue) and the MNZ:GAL cocrystal formation (peaks at ca. 151 ppm, 145.5 ppm, 123 ppm) (Figure S15B, Figure S16C and Figure S16D), while no dissolution was observed in the ^13^C­{^1^H} NMR spectra (Figure S15F). This is consistent with the spectra collected for MNZ:GAL D_2_O (0.14 μL mg^–1^). However, to assess the exact phase distribution, the spectral region of 20–10 ppm of the ^1^H–^13^C CP/MAS NMR spectra was analyzed. The first spectrum recorded at 90 min (CLASSIC-S2, 90–250 min) did not suggest the presence of MNZ:GAL F1s in the product, but the 410 min (CLASSIC-S2, 410–570 min) spectrum clearly reveals the appearance of the stable form of the MNZ:GAL cocrystal (Figure 7C). This would indicate that at 90 min mark, no traces of MNZ:GAL F1s were detectable in the sample. However, the intensity contour plots comprising all MNZ:GAL D_2_O ^13^C­{^1^H} NMR spectra acquired from the beginning to the 90 min mark suggest otherwise. The MNZ methyl group signal in the 0.28 μL mg^–1^ sample (Figure S15D) is significantly broader than the respective peak in the spectra of the 0.14 μL mg^–1^ data set (Figure S15C). This was also corroborated by the width of the peak measured at its base. The width at the 90 min mark (^13^C­{^1^H} NMR) appears to be 720 and 893 Hz in the 0.14 and 0.28 μL mg^–1^ samples, respectively. This finding lends weight to the hypothesis of facilitated MNZ:GAL F1s formation in the environment of increased solvent-to-powder ratio.

In the MNZ:GAL MeOD system, changing the solvent-to-powder ratio affected the ^1^H–^13^C CP/MAS NMR spectra quite unexpectedly. While both MNZ and GAL were clearly visible in the 90 and 410 min spectra of the MeOD 0.14 μL mg^–1^ ratio data set (Figure S17A), a review of the respective data of the MeOD 0.28 μL mg^–1^ ratio resulted in the complete disappearance of the MNZ and GAL peaks (Figure S17B). At the same time, widening of the peaks was observed, compared to the lower solvent-to-powder ratio of MeOD (0.14 μL mg^–1^, Figure S17A) and the corresponding ratio of D_2_O (0.28 μL mg^–1^, Figure S15B). The same remains true for the MNZ methyl group region presented in Figure 7D: it shows wide lines with no distinct peaks, regardless of the moment of the acquisition within the CLASSIC NMR sequence, which had not been observed previously in any of the other samples (Figure 7A–7C). Therefore, identifying the phase distribution in the MNZ:GAL MeOD (0.28 μL mg^–1^) remains a challenge. The 90 min spectrum points toward the mixture of neat MNZ and MNZ:GAL F2m, while the upfield shift in the 410 min spectrum suggests the formation of MNZ:GAL F1s. The ^13^C­{^1^H} NMR spectra were then analyzed, and the contour plots also denote the cocrystallization of MNZ:GAL F1s in the MeOD 0.28 μL mg^–1^ sample (Figure S17D) while no such indication was noticed at the MeOD 0.14 μL mg^–1^ ratio (Figure S17C). This supports the hypothesis, made based on evaluation of the MNZ:GAL D_2_O system, that a higher solvent-to-powder ratio favors the cocrystallization of MNZ:GAL F1s compared to a lower solvent amount which promotes the assembly of MNZ:GAL F2m.

During the analysis of the MNZ:GAL MeOD data sets, the comparison of 90 and 410 min ^1^H–^13^C CP/MAS NMR spectra revealed that the intensity of the MNZ methyl group signal decreased over time at both ratios (0.14 and 0.28 μL mg^–1^). Meanwhile, no similar pattern was observed for the MNZ:GAL D_2_O samples (Figure 7). This was further followed by measuring the area under the curve of these peaks, as summarized in Table S5. The review of the obtained spectral areas confirmed the initial observation that no decrease (0%, 0.14 μL mg^–1^) or a minor decrease (5%, 0.28 μL mg^–1^) was observed for the MNZ:GAL D_2_O data. On the other hand, there was almost 10% and over 20% decrease of the spectral area for 0.14 and 0.28 μL mg^–1^ of MeOD, respectively. The signal loss in the ^1^H–^13^C CP/MAS NMR spectra could indicate a transition of components from the solid to the liquid phase; therefore, the ^13^C­{^1^H} NMR data were analyzed. In the ^13^C­{^1^H} NMR data set of the MNZ:GAL MeOD (0.14 μL mg^–1^) sample, GAL dissolution was detectable (Figure S17E) and the intensity of the GAL peaks further increased in the 0.28 μL mg^–1^ ratio sample (Figure S17F). A closer examination of the ^13^C­{^1^H} NMR of the MNZ:GAL MeOD (0.28 μL mg^–1^) data set revealed that the amount of the GAL dissolved in MeOD increased over time (Figure S18), indicating the ongoing solid-to-liquid phase transition. These ^13^C­{^1^H} NMR observations contrast with those noticed for the TP:BZ system as BZ, regardless of the solvent used, presented the maximum dissolution at ca. 15–20 min into the CLASSIC NMR experiments, after which it showed a gradual decrease (Figure 6E and Figure S13C). We hypothesized it might arise from the solubility differences, i.e., the assembling MNZ:GAL cocrystal is more soluble in MeOH than neat components. Therefore, we attempted to evaluate the cocrystal solubility using HPLC. However, only the solubility of the stable MNZ:GAL F1s polymorph could be determined as suspensions of the physical mixture or the metastable MNZ:GAL F2m form underwent rapid (<5 min) phase transitions. The solubility of the MNZ:GAL F1s cocrystal does not support the suggested hypothesis (Figure S6). Nevertheless, the solubility and dissolution rate of the initially forming MNZ:GAL F2m polymorph may differ significantly from those of the stable form.

For MNZ:GAL TOL_d_, only the 0.28 μL mg^–1^ ratio experiment was performed, and no cocrystallization occurred within 250 min of data acquisition. More details can be found in ESI (Figure S19).

### CLASSIC NMR vs LAG

Due to no systematic study on ball milling of the MNZ:GAL system and differences in grinding parameters during liquid-assisted grinding of the TP:BZ mixture, such as milling time, frequency, experimental setup (the volume and material of a milling jar; the number, weight and material of the milling balls), the loading of a mill and the solvent-to-powder ratio, that were applied,^8,20,67^ we decided to conduct the milling experiments on both systems keeping most of the parameters constant. The only variables were: (i) the solvent: the same set of solvents as in the CLASSIC NMR experiments was used, i.e., H_2_O, MeOH, and TOL; (ii) the amount of solvent added: to correspond to the two solvent-to-powder ratios employed during the NMR studies. To ensure this, either 50 μL (η = 0.17 μL mg^–1^) or 100 μL (η = 0.33 μL mg^–1^) of a solvent was added to 300 mg of an equimolar physical mixture (TP:BZ PM or MNZ:GAL PM), which represented the 0.14 and 0.28 μL mg^–1^ ratios used during CLASSIC NMR acquisitions, respectively. This approach not only allowed us to compare a polymorphic behavior of both studied systems but also to review the CLASSIC NMR results.

Before the effect of the solvent type and volume was evaluated, neat grinding experiments were carried out. In the TP:BZ system, the standard 20 min grinding procedure revealed an assembly of a metastable TP:BZ F1m polymorph and the remaining residue of the starting materials (2θ = 7.14°, 7.98°), which disappeared after an additional 20 min of grinding. No signs of the TP:BZ F2s were noticed (Figure 8A). The subsequent analysis of neatly ground MNZ:GAL PM suggested that the two studied systems behaved vastly differently. Neither 20 nor 40 min of neat grinding produced a neat MNZ:GAL cocrystal. The collected PXRD patterns showed that the measured samples remained mostly the MNZ:GAL physical mixture with some traces of MNZ:GAL F2m (2θ = 26.06°) and a high degree of amorphization after grinding (Figure 8B).

**Figure 8:**
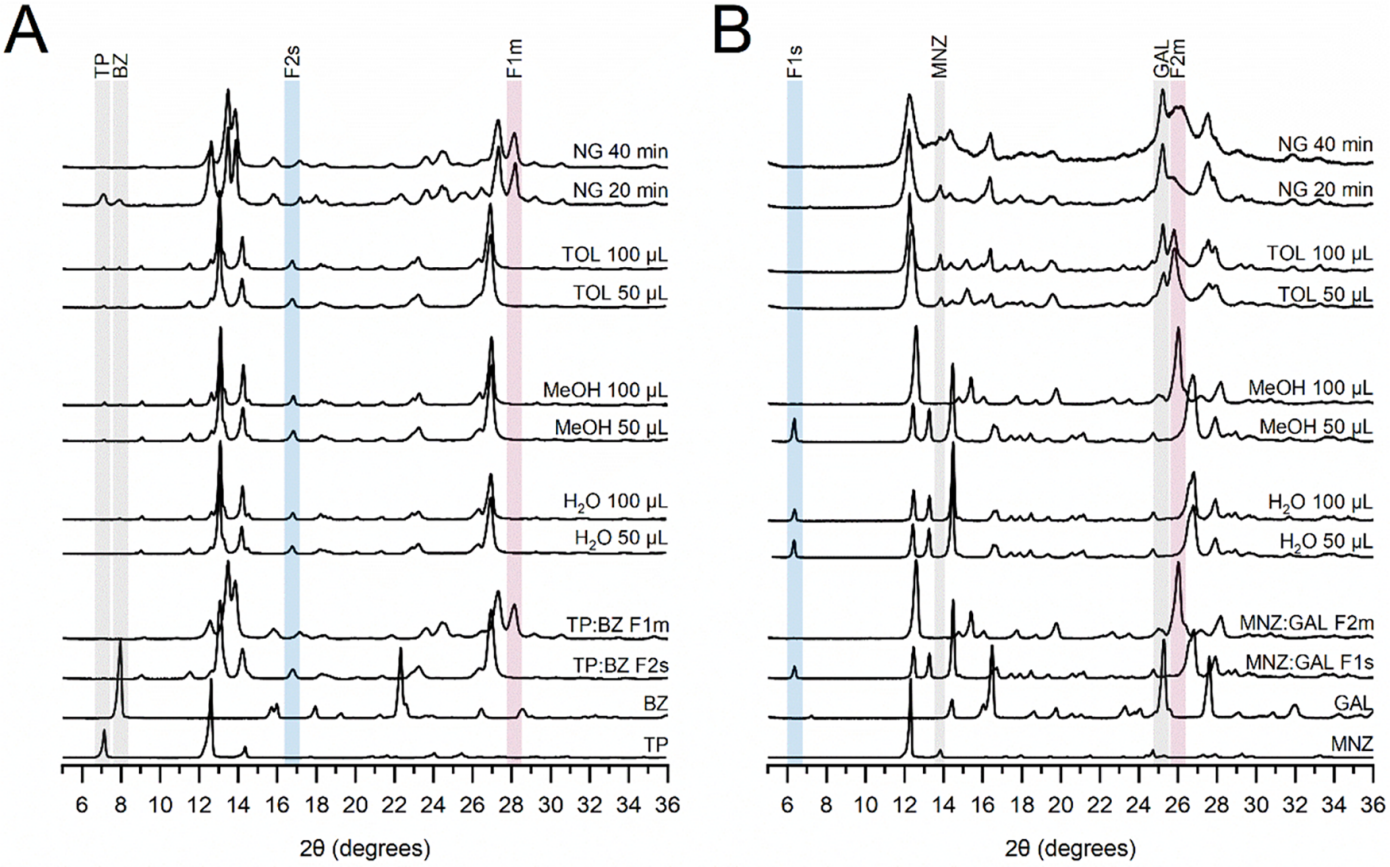
Experimental PXRD patterns of the products of LAG (solvents: H_2_O, MeOH, TOL) compared with the reference PXRD patterns of the starting materials and cocrystals: (A) TP:BZ system and (B) MNZ:GAL system. The addition of 50 or 100 μL of solvent corresponds to η = 0.17 μL mg^–1^ or η = 0.33 μL mg^–1^, respectively. The starting materials are marked with gray rectangles, while pink and blue rectangles were assigned to the metastable and stable forms of the cocrystals, respectively.

The PXRD patterns of the products of liquid-assisted grinding of the TP:BZ PM showed that, upon the addition of any solvent (H_2_O, MeOH, TOL) in volumes of either 50 or 100 μL volume, cocrystallization of the TP:BZ F2s polymorph occurred (Figure 8A). The appearance of TP:BZ F2s in H_2_O- and MeOH-assisted grinding was expected due to previously reported TP:BZ ball milling data.^8,20^ At the same time, it was presumed that TOL, as a low-polarity solvent, would produce a metastable polymorph, as has been reported for other compounds when ground or subjected to stirred suspension experiments with TOL.^73,82^ However, for the TP:BZ system, it appears that a nonpolar solvent, e.g., cyclohexane, heptane, or pentane,^8^ or a low-polarity solvent must be added in a concentration lower than the one planned in our study,^10^ to ensure crystallization of the TP:BZ metastable form.

Therefore, the CLASSIC data for the TP:BZ samples spun in the presence of D_2_O and MeOD are consistent with the results of the LAG experiments performed with H_2_O and MeOH. This indicates that a lower solvent-to-powder ratio was sufficient to drive crystallization toward the stable TP:BZ cocrystal form in both LAG and CLASSIC NMR experiments. However, the accuracy of the CLASSIC NMR and LAG data for the TP:BZ TOL system cannot be fully assessed because of uncertainty regarding the cocrystal polymorph produced in MAS-induced experiments.

The data for MNZ:GAL show that the solvent-to-powder ratio seems to be of greater importance than in the TP:BZ system, in both LAG and CLASSIC NMR. In the LAG with H_2_O, both 50 and 100 μL led to the formation of pure MNZ:GAL F1s (2θ = 6.38°), and so did LAG in the presence of 50 μL of MeOH (Figure 8B). Followingly, the unexpected outcome was observed in the experiment with 100 μL of MeOH as the reflexes in the PXRD pattern (e.g., 2θ = 26.06°) revealed the appearance of metastable MNZ:GAL F2m. As mentioned before, the increase in the solvent quantity (η) during grinding usually leads to accelerating the rate of cocrystal formation.^10,12,81^ This is the approach we implemented in CLASSIC NMR experiments, and indeed, the higher solvent-to-powder ratio (0.28 μL mg^–1^) facilitated the MNZ:GAL F1s cocrystallization, while the lower ratio (0.14 μL mg^–1^) resulted in the assembly of MNZ:GAL F2m, which was observed for both D_2_O and MeOD. However, the increase of the solvent amount during LAG of MNZ:GAL system seems to have a decelerating effect, as proven not only by the MeOH data but also TOL:MNZ:GAL TOL 50 μL sample, which comprised more cocrystal (MNZ:GAL F2m, 2θ = 26.06°) and less residue of unreacted materials (2θ = 13.87°, 25.28°) compared to the 100 μL sample. The consistent behavior across two solvents suggests a dependence on solvent quantity rather than type. Hasa et al.^11^ observed that the polymorphic outcome of the caffeine–anthranilic acid cocrystallization differs depending on the η ratio, despite the same solvent being used. It was also noticed that some solvents (e.g., methanol, ethanol) promoted the assembly of a more stable Form I at the η intermediate ratios (0.2–0.3 μL mg^–1^), while both lower and higher η values produced Form II of lower relative stability compared to Form I. The same phenomenon was reported by Gonnet et al.^7^ and named a ‘η-sweet-spot’ (η_max_) at which the maximum conversion is achieved, and when η is either above or below η_max_, the conversion drops. The differences between the results of LAG and CLASSIC NMR for the MNZ:GAL system raise a question of whether the ‘η-sweet-spot’ for both methods is the same. Additionally, although the TP:BZ system appears less susceptible to variations in the η ratio, the MNZ:GAL system may exhibit greater sensitivity to such changes. Friščić et al.^12^ observed that the effect of η is most pronounced in systems where the cocrystal components exhibit incongruent solubilities. This applies to the MNZ:GAL MeOH system but also to TP:BZ MeOH, where no such η-dependent behavior was observed. The observed effect of solvent quantity during LAG could also be attributed to less efficient mechanical energy transfer when larger amounts of solvent are present in the milling jar.^83^ Additionally, different pressures and mixing properties^25^ of a milling jar during LAG and the NMR rotor under the MAS conditions might result in a different reaction outcome.

When it comes to corroborating the PXRD data with the NMR results, the accuracy of the LAG and CLASSIC NMR experiments might also be system-dependent. Undoubtedly, water (H_2_O, D_2_O) is more effective in promoting cocrystallization of both TP:BZ and MNZ:GAL systems than other solvents studied, as revealed by both LAG and CLASSIC NMR experiments. Moreover, the polarity and quantity of the solvent have a greater impact on the cocrystallization outcome in the MNZ:GAL than TP:BZ system.

## Discussion

For the first time, CLASSIC NMR was applied to monitor *in situ* solid-state mechanochemical cocrystallization process. We successfully mimicked the liquid-assisted grinding process and identified the same solid-state phases as TRIS-PXRD, i.e., hydrates and metastable cocrystal polymorphs, confirming its suitability for monitoring such complex reactions. Moreover, its accessibility offers a practical advantage over synchrotron-based methods as it utilizes a standard solid-state NMR setup. Due to modifications introduced into the CLASSIC NMR protocol, no isotopic enrichment of the compounds was needed, which proves the feasibility and versatility of this approach. Additionally, sealing solvent in glass capillaries and using them to deliver liquids prevented missing the early stages of the reaction, which may occur when solvent is added directly to the NMR rotor.

CLASSIC NMR provided insights into the behavior of the liquid phase of the systems, revealing processes such as dissolution and cocrystal formation from solution (seen as either changes in intensity in ^13^C­{^1^H} NMR or FWHM of the peaks in ^1^H NMR spectra). These are not detectable by either ^1^H–^13^C CP/MAS NMR or diffraction-based methods such as TRIS-PXRD, which supports the importance of employing CLASSIC NMR in a standard reaction monitoring protocol of multiphase reactions. The observations highlight the solvent’s critical role in the cocrystallization mechanism (Figure 9). If the system produced a hydrated form, e.g., TP:BZ D_2_O, the hydrate TP-MH appeared immediately upon the addition of water preceding the assembly of a metastable cocrystal. At this point, no dissolution of BZ was observed due to water being entrapped in the crystal lattice of TP-MH. As the TP:BZ cocrystal is more thermodynamically stable compared to TP-MH, reaction proceeded toward cocrystallization of TP and BZ. This released the water in which, subsequently, BZ dissolved. The amount of the dissolved BZ reached a maximum and then decreased, which could be associated with the formation of the metastable form and further transition toward the stable polymorph of lower solubility. For nonhydrated systems, e.g., TP:BZ MeOD, the dissolution of BZ was observed from the beginning as the solvent was not involved in the assembly of a solvate. In MNZ:GAL D_2_O, GAL-MH appeared prior to cocrystallization, but when water was released from the hydrate lattice, no dissolution was observed, in contrast to TP:BZ D_2_O. However, this is consistent with the solubility data as neither MNZ nor GAL was expected to dissolve in water. The behavior of MNZ:GAL MeOD was analogous to TP:BZ MeOD, as no lag in GAL dissolution was observed. The only noticeable difference is the lack of maximum dissolution of GAL and its further decrease, as described for BZ. We presume it results from differences in phase distribution in both samples. While in TP:BZ MeOD, the stable cocrystal form is predominant, in MNZ:GAL MeOD, the sample remains mostly in a metastable form. In the case of further transition toward MNZ:GAL F1s, a similar drop in liquid-phase signal intensity could appear. Nevertheless, regardless of the system, the water-induced reactions exhibited the fastest kinetics, likely due to the involvement of intermediate hydrates or the higher polarity of water compared to other tested solvents. At the same time, the kinetics does not seem to be solubility-dependent. In addition, the optimal amount of solvent required to facilitate cocrystallization was found to be system-dependent as the 0.14 μL mg^–1^ ratio was sufficient to assemble the stable TP:BZ F2s polymorph, but at the same time, the MNZ:GAL system needed the solvent-to-powder ratio of 0.28 μL mg^–1^ to show any traces of MNZ:GAL F1s (Figure 9).

**Figure 9:**
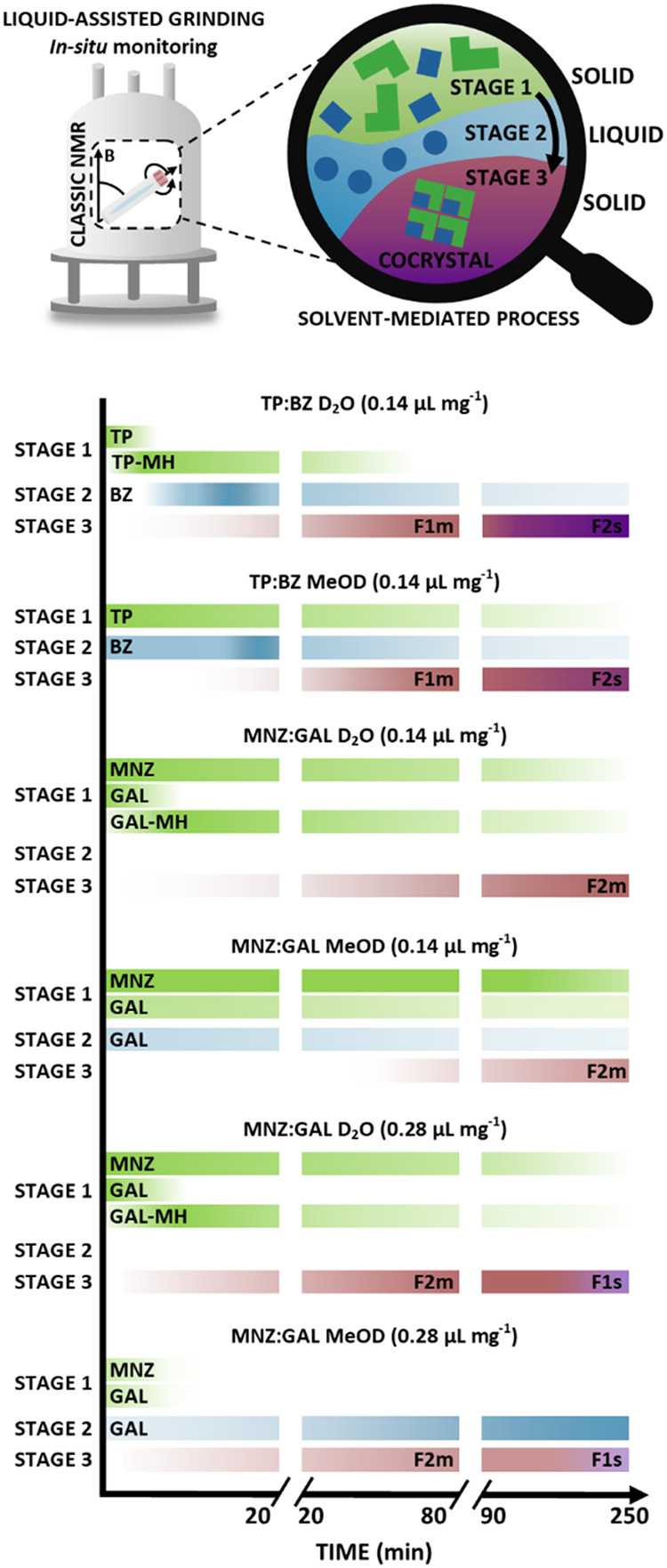
Schematic representation of phase evolution over time in CLASSIC NMR experiments (the TP:BZ and MNZ:GAL systems tested using D_2_O and MeOD at 0.14 μL mg^–1^ and, where applicable, 0.28 μL mg^–1^).

The developed approach has some limitations that are worth noting. As revealed in the course of the study, the CLASSIC NMR might not always accurately reflect the ball milling process; thus, corroborating with the outcome of standard grinding experiments is recommended. Most likely, this is due to the lower pressure and limited mixing in an NMR rotor compared to a milling jar, which results in slower conversion. However, this could also be considered an asset as it supports the detection of intermediate phases, particularly with regard to the time required to record a ^1^H–^13^C CP/MAS NMR spectrum of sufficient signal-to-noise (S/N) ratio. To achieve the maximum sensitivity during the NMR acquisition, and hence to achieve the best possible time resolution for *in situ* studies, the CLASSIC NMR experiments are advised to be carried out using a high-field NMR spectrometer. Testing other NMR techniques for signal enhancement, e.g., insensitive nuclei enhanced by polarization transfer (INEPT) for liquid-phase detection, is also recommended. Depending on the properties of the investigated materials (including their structure) or the kinetics of the reaction, the suggested experimental sequences might require adjusting, e.g., the length of sequence, the number of scans collected, the order of NMR experiments in the CLASSIC NMR sequence, the recycle delay used, or the nuclei observed. The presence of mobile moieties in the structures is advantageous but not required; in their absence, the use of isotopically enriched materials may be preferable. We also decided to use a 4 mm probe as smaller rotors, i.e., 3.2 mm, did not fit a capillary with the solvent. Using smaller diameter rotors and a higher rate of MAS would be beneficial, especially for ^1^H acquisition and peak resolution but would also require adding solvent directly to the rotor or performing the sample preparation and transferring of a MAS rotor into the probe at temperatures much lower than that needed for triggering the cocrystal formation. Consequently, the problem with overlooking the beginning of the reaction might arise. Alternatively, the application of homonuclear decoupling sequences to enhance the ^1^H spectral resolution could be explored.

## Conclusions

For the first time, we report a successful implementation of the CLASSIC NMR protocol to mimic and monitor *in situ* a process of solvent-mediated mechanochemical cocrystallization using a standard solid-state NMR setup. Using this approach, we studied two model pharmaceutical polymorphic cocrystals with three different solvents. Each exhibited distinct cocrystallization mechanisms or reaction kinetics, influenced by the solvent type, its amount, and the solubility of the neat compounds and resulting cocrystals. Correlating the solid-state phase data with liquid-phase observations enabled us to elucidate these mechanisms, demonstrating that *in situ* CLASSIC NMR is a powerful and accessible tool for monitoring cocrystallization, complementary to TRIS-PXRD experiments.

The developed protocol can be readily applied to monitor other reactions, particularly multiphase systems where both solid- and liquid-state phases, as well as their transitions, occur during the reaction, which are not necessarily mechanochemically induced. Potential applications include the mechanosynthesis of organic compounds, the incorporation of molecules into polymer matrices, and the dissolution from carrier materials. In pharmaceutical contexts, this encompasses mechanochemical synthesis of APIs, preparation of amorphous solid dispersions (API and polymer interactions), and elucidation of API release mechanisms from porous drug carriers. The ability to simultaneously observe changes in both solid and liquid phases offers a deeper understanding of the reaction mechanisms. Insights gained from *in situ* NMR studies could ultimately improve process control, leading to enhanced therapeutic efficacy and safety of pharmaceutical products.

## Supplementary Material



## Notes

^1^H and ^13^C NMR peaks assignment details, solubility data, additional tables and figures comprising PXRD and CLASSIC NMR data (PDF)

## ⊥

Deceased author (25th March 2023).

## References

[1] Aitipamula S., Banerjee R., Bansal A. K., Biradha K., Cheney M. L., Choudhury A. R., Desiraju G. R., Dikundwar A. G., Dubey R., Duggirala N. (2012). Polymorphs, Salts, and
Cocrystals: What’s in a Name?. Cryst.
Growth Des..

[2] Shan N., Zaworotko M. J. (2008). The Role of Cocrystals in Pharmaceutical Science. Drug Discovery Today.

[3] Cruz-Cabeza A. J., Reutzel-Edens S. M., Bernstein J. (2015). Facts and Fictions about Polymorphism. Chem. Soc. Rev..

[4] Duggirala N. K., Perry M. L., Almarsson Ö., Zaworotko M. J. (2016). Pharmaceutical
Cocrystals: Along the Path to Improved Medicines. Chem. Commun..

[5] Stolar T., Lukin S., Tireli M., Sović I., Karadeniz B., Kereković I., Matijašić G., Gretić M., Katančić Z., Dejanović I. (2019). Control of Pharmaceutical Cocrystal Polymorphism on Various Scales
by Mechanochemistry: Transfer from the Laboratory Batch to the Large-Scale
Extrusion Processing. ACS Sustainable Chem.
Eng..

[6] Trzeciak K., Dudek M. K., Potrzebowski M. J. (2024). Mechanochemical
Transformations of
Pharmaceutical Cocrystals: Polymorphs and Coformer Exchange. Chem. – Eur. J..

[7] Gonnet L., Borchers T. H., Lennox C. B., Vainauskas J., Teoh Y., Titi H. M., Barrett C. J., Koenig S. G., Nagapudi K., Friščić T. (2023). The “ *η*-Sweet-Spot” (*η*
_max_) in Liquid-Assisted Mechanochemistry: Polymorph Control
and the Role of a Liquid Additive as Either a Catalyst or an Inhibitor
in Resonant Acoustic Mixing (RAM). Faraday Discuss..

[8] Fischer F., Heidrich A., Greiser S., Benemann S., Rademann K., Emmerling F. (2016). Polymorphism
of Mechanochemically Synthesized Cocrystals:
A Case Study. Cryst. Growth Des..

[9] Hasa D., Carlino E., Jones W. (2016). Polymer-Assisted Grinding, a Versatile
Method for Polymorph Control of Cocrystallization. Cryst. Growth Des..

[10] Belenguer A. M., Lampronti G. I., De Mitri N., Driver M., Hunter C. A., Sanders J. K. M. (2018). Understanding
the Influence of Surface Solvation and
Structure on Polymorph Stability: A Combined Mechanochemical and Theoretical
Approach. J. Am. Chem. Soc..

[11] Hasa D., Miniussi E., Jones W. (2016). Mechanochemical
Synthesis of Multicomponent
Crystals: One Liquid for One Polymorph? A Myth to Dispel. Cryst. Growth Des..

[12] Friščić T., Childs S. L., Rizvi S. A. A., Jones W. (2009). The Role of Solvent
in Mechanochemical and Sonochemical Cocrystal Formation: A Solubility-Based
Approach for Predicting Cocrystallisation Outcome. CrystEngComm.

[13] Kulla H., Becker C., Michalchuk A. A. L., Linberg K., Paulus B., Emmerling F. (2019). Tuning the Apparent Stability of Polymorphic Cocrystals
through Mechanochemistry. Cryst. Growth Des..

[14] Casali L., Carta M., Michalchuk A. A. L., Delogu F., Emmerling F. (2024). Kinetics of
the Mechanically Induced Ibuprofen–Nicotinamide Co-Crystal
Formation by *in Situ* X-Ray Diffraction. Phys. Chem. Chem. Phys..

[15] Linberg K., Sander P. C., Emmerling F., Michalchuk A. A. L. (2024). *In Situ* Investigation of Controlled
Polymorphism in Mechanochemistry
at Elevated Temperature. RSC Mechanochem..

[16] Linberg K., Röder B., Al-Sabbagh D., Emmerling F., Michalchuk A. A. L. (2023). Controlling
Polymorphism in Molecular Cocrystals by
Variable Temperature Ball Milling. Faraday Discuss..

[17] Mazzeo P. P., Prencipe M., Feiler T., Emmerling F., Bacchi A. (2022). On the Mechanism of Cocrystal Mechanochemical
Reaction
via Low Melting Eutectic: A Time-Resolved In Situ Monitoring Investigation. Cryst. Growth Des..

[18] Michalchuk A. A. L., Hope K. S., Kennedy S. R., Blanco M. V., Boldyreva E. V., Pulham C. R. (2018). Ball-Free Mechanochemistry: *In Situ* Real-Time Monitoring of Pharmaceutical Co-Crystal
Formation by Resonant
Acoustic Mixing. Chem. Commun..

[19] Kulla H., Greiser S., Benemann S., Rademann K., Emmerling F. (2017). Knowing When
To Stop—Trapping Metastable Polymorphs in Mechanochemical Reactions. Cryst. Growth Des..

[20] Lampronti G. I., Michalchuk A. A. L., Mazzeo P. P., Belenguer A. M., Sanders J. K. M., Bacchi A., Emmerling F. (2021). Changing the
Game of Time Resolved X-Ray Diffraction on the Mechanochemistry Playground
by Downsizing. Nat. Commun..

[21] Rautenberg M., Bhattacharya B., Witt J., Jain M., Emmerling F. (2022). *In
Situ* Time-Resolved Monitoring of Mixed-Ligand Metal–Organic
Framework Mechanosynthesis. CrystEngComm.

[22] Martins I.
C. B., Carta M., Haferkamp S., Feiler T., Delogu F., Colacino E., Emmerling F. (2021). Mechanochemical *N*-Chlorination Reaction
of Hydantoin: In Situ Real-Time Kinetic Study
by Powder X-Ray Diffraction and Raman Spectroscopy. ACS Sustainable Chem. Eng..

[23] Ardila-Fierro K. J., Lukin S., Etter M., Užarević K., Halasz I., Bolm C., Hernández J. G. (2020). Direct
Visualization of a Mechanochemically Induced Molecular Rearrangement. Angew. Chem., Int. Ed..

[24] Silva I. d’. A., Bartalucci E., Bolm C., Wiegand T. (2023). Opportunities and Challenges
in Applying Solid-State NMR Spectroscopy in Organic Mechanochemistry. Adv. Mater..

[25] Bartalucci E., Schumacher C., Hendrickx L., Puccetti F., d’Anciães
Almeida Silva I., Dervişoğlu R., Puttreddy R., Bolm C., Wiegand T. (2023). Disentangling the Effect of Pressure
and Mixing on a Mechanochemical Bromination Reaction by Solid-State
NMR Spectroscopy. Chem. – Eur. J..

[26] Gupta S., Kobayashi T., Hlova I. Z., Goldston J. F., Pruski M., Pecharsky V. K. (2014). Solvent-Free Mechanochemical Synthesis of Alane, AlH_3_: Effect of Pressure on the Reaction Pathway. Green Chem..

[27] Friščić T., Halasz I., Beldon P. J., Belenguer A. M., Adams F., Kimber S. A. J., Honkimäki V., Dinnebier R. E. (2013). Real-Time and in Situ Monitoring of Mechanochemical
Milling Reactions. Nat. Chem..

[28] Halasz I., Kimber S. A. J., Beldon P. J., Belenguer A. M., Adams F., Honkimäki V., Nightingale R. C., Dinnebier R. E., Friščić T. (2013). In Situ and
Real-Time Monitoring of Mechanochemical Milling Reactions Using Synchrotron
X-Ray Diffraction. Nat. Protoc..

[29] Schiffmann J. G., Emmerling F., Martins I. C. B., Van Wüllen L. (2020). In-Situ Reaction
Monitoring of a Mechanochemical Ball Mill Reaction with Solid State
NMR. Solid State Nucl. Magn. Reson..

[30] Xu Y., Champion L., Gabidullin B., Bryce D. L. (2017). A Kinetic Study
of Mechanochemical Halogen Bond Formation by in Situ ^31^P Solid-State NMR Spectroscopy. Chem. Commun..

[31] Dudek M. K., Trzeciak K., Tajber L., Zając J., Kaźmierski S., Pindelska E., Makowski T., Svyntkivska M., Potrzebowski M. J. (2024). A New Look
at the Mechanism of Cocrystal Formation
and Coformers Exchange in Processes Forced by Mechanical and/or Thermal
Stimuli – *Ex Situ* and *in Situ* Studies of Low-Melting Eutectic Mixtures. Chem. – Eur. J..

[32] Mandala V. S., Loewus S. J., Mehta M. A. (2014). Monitoring
Cocrystal Formation via
In Situ Solid-State NMR. J. Phys. Chem. Lett..

[33] Hareendran C., Alsirawan B., Paradkar A., Ajithkumar T. G. (2024). In Situ
Monitoring of Competitive Coformer Exchange Reaction by ^1^ H MAS Solid-State NMR. Mol. Pharmaceutics.

[34] Hughes C. E., Williams P. A., Harris K. D. M. (2014). CLASSIC NMR”: An In-Situ NMR
Strategy for Mapping the Time-Evolution of Crystallization Processes
by Combined Liquid-State and Solid-State Measurements. Angew. Chem., Int. Ed..

[35] Harris K. D. M., Hughes C. E., Williams P. A., Edwards-Gau G. R. (2017). `NMR
Crystallization’: *In-Situ* NMR Techniques for
Time-Resolved Monitoring of Crystallization Processes. Acta Crystallogr., Sect. C: Struct. Chem..

[36] Ghosh
Biswas R., Soong R., Jenne A., Bastawrous M., Simpson M. J., Simpson A. J. (2023). SASSY NMR: Simultaneous Solid and
Solution Spectroscopy. Angew. Chem., Int. Ed..

[37] Friščić T., Jones W. (2009). Recent Advances in Understanding the Mechanism of Cocrystal Formation
via Grinding. Cryst. Growth Des..

[38] Arhangelskis M., Bučar D.-K., Bordignon S., Chierotti M. R., Stratford S. A., Voinovich D., Jones W., Hasa D. (2021). Mechanochemical
Reactivity Inhibited, Prohibited and Reversed by Liquid Additives:
Examples from Crystal-Form Screens. Chem. Sci..

[39] Xu M., Harris K. D. M., Thomas J. M., Vaughan D. E. W. (2007). Probing the Evolution
of Adsorption on Nanoporous Solids by In Situ Solid-State NMR Spectroscopy. ChemPhysChem.

[40] Xu M., Harris K. D. M., Thomas J. M. (2008). Mapping
the Evolution of Adsorption
of Water in Nanoporous Silica by in Situ Solid-State ^1^H
NMR Spectroscopy. J. Am. Chem. Soc..

[41] Xu M., Harris K. D. M., Thomas J. M. (2009). In Situ
Solid-State 1H NMR Studies
of Hydration of the Solid Acid Catalyst ZSM-5 in Its Ammonium Form. Solid State Nucl. Magn. Reson..

[42] Xu M., Harris K. D. M., Thomas J. M. (2009). Preferential
Clustering of Water
Molecules During Hydration of the Ammonium Form of the Solid Acid
Catalyst ZSM-5. Catal. Lett..

[43] Harris K. D. M., Xu M., Thomas J. M. (2009). Probing
the Evolution of Water Clusters
during Hydration of the Solid Acid Catalyst H-ZSM-5. Philos. Mag..

[44] Fischer F., Schmidt M. U., Greiser S., Emmerling F. (2016). The Challenging
Case of the Theophylline–Benzamide Cocrystal. Acta Crystallogr., Sect. C: Struct. Chem..

[45] Zheng K., Li A., Wu W., Qian S., Liu B., Pang Q. (2019). Preparation,
Characterization, in Vitro and in Vivo Evaluation of Metronidazole–Gallic
Acid Cocrystal: A Combined Experimental and Theoretical Investigation. J. Mol. Struct..

[46] Seera R., Guru Row T. N. (2020). Evaluation of Cocrystallization
Outcomes of Multicomponent
Adducts: Rapid Fabrication to Achieve Uniform Particle Size Distribution
Using Thermal Inkjet Printing. Cryst. Growth
Des..

[47] Guo C., Holland G. P. (2015). Alanine Adsorption and Thermal Condensation at the
Interface of Fumed Silica Nanoparticles: A Solid-State NMR Investigation. J. Phys. Chem. C.

[48] Zizak I. (2016). MySpot: A
Versatile Microfocussing Station for Scanning Methods at BESSY II. J. Large-Scale Res. Facil. JLSRF.

[49] Benecke G., Wagermaier W., Li C., Schwartzkopf M., Flucke G., Hoerth R., Zizak I., Burghammer M., Metwalli E., Müller-Buschbaum P., Trebbin M. (2014). A Customizable Software for Fast Reduction and Analysis of Large
X-Ray Scattering Data Sets: Applications of the New *DPDAK* Package to Small-Angle X-Ray Scattering and Grazing-Incidence Small-Angle
X-Ray Scattering. J. Appl. Crystallogr..

[50] Baek S.-J., Park A., Ahn Y.-J., Choo J. (2015). Baseline Correction
Using Asymmetrically Reweighted Penalized Least Squares Smoothing. Analyst.

[51] Clark S. J., Segall M. D., Pickard C. J., Hasnip P. J., Probert M. I. J., Refson K., Payne M. C. (2005). First Principles Methods Using CASTEP. Z. Kristallogr. - Cryst. Mater..

[52] Ebisuzaki Y., Boyle P. D., Smith J. A. (1997). Methylxanthines.
I. Anhydrous Theophylline. Acta Crystallogr.,
Sect. C: Cryst. Struct. Commun..

[53] Liu H., Stephen Chan H. C., Zhang L., Lu Y., Li J., Li J., Li L., Zhou Z. (2023). The Molecular Mechanisms of Plasticity
in Crystal Forms of Theophylline. Chin. Chem.
Lett..

[54] Blake C. C. F., Small R. W. H. (1972). The Crystal Structure of Benzamide. Acta Crystallogr., Sect. B: Struct. Sci..

[55] Blaton N. M., Peeters O. M., De Ranter C. J. (1979). 2-(2-Methyl-5-Nitro-1-Imidazolyl)­Ethanol
(Metronidazole). Acta Crystallogr., Sect. B:
Struct. Sci..

[56] Braun D. E., Bhardwaj R. M., Florence A. J., Tocher D. A., Price S. L. (2013). Complex
Polymorphic System of Gallic Acid—Five Monohydrates, Three
Anhydrates, and over 20 Solvates. Cryst. Growth
Des..

[57] Okabe N., Kyoyama H., Suzuki M. (2001). Gallic Acid Monohydrate. Acta Crystallogr., Sect. E: Struct. Rep. Online.

[58] Perdew J. P., Burke K., Ernzerhof M. (1996). Generalized
Gradient Approximation
Made Simple. Phys. Rev. Lett..

[59] Vanderbilt D. (1990). Soft Self-Consistent
Pseudopotentials in a Generalized Eigenvalue Formalism. Phys. Rev. B.

[60] Tkatchenko A., Scheffler M. (2009). Accurate Molecular
Van Der Waals Interactions from
Ground-State Electron Density and Free-Atom Reference Data. Phys. Rev. Lett..

[61] Pickard C. J., Mauri F. (2001). All-Electron Magnetic
Response with Pseudopotentials: NMR Chemical
Shifts. Phys. Rev. B.

[62] Yates J. R., Pickard C. J., Mauri F. (2007). Calculation
of NMR Chemical Shifts
for Extended Systems Using Ultrasoft Pseudopotentials. Phys. Rev. B.

[63] Hodgkinson P. (2020). NMR Crystallography
of Molecular Organics. Prog. Nucl. Magn. Reson.
Spectrosc..

[64] Baias M., Widdifield C. M., Dumez J.-N., Thompson H. P. G., Cooper T. G., Salager E., Bassil S., Stein R. S., Lesage A., Day G. M., Emsley L. (2013). Powder Crystallography
of Pharmaceutical
Materials by Combined Crystal Structure Prediction and Solid-State
1H NMR Spectroscopy. Phys. Chem. Chem. Phys..

[65] United States Pharmacopeial Convention . Solubility (<1090>). In United States Pharmacopeia and National Formulary (USP 47–NF 42), Rockville, MD, 2024.

[66] Kozakiewicz-Latała M., Nartowski K. P., Dominik A., Malec K., Gołkowska A. M., Złocińska A., Rusińska M., Szymczyk-Ziółkowska P., Ziółkowski G., Górniak A., Karolewicz B. (2022). Binder Jetting 3D Printing of Challenging
Medicines: From Low Dose Tablets to Hydrophobic Molecules. Eur. J. Pharm. Biopharm..

[67] Eddleston M. D., Arhangelskis M., Fábián L., Tizzard G. J., Coles S. J., Jones W. (2016). Investigation
of an Amide-Pseudo
Amide Hydrogen Bonding Motif within a Series of Theophylline:Amide
Cocrystals. Cryst. Growth Des..

[68] Thun J., Seyfarth L., Butterhof C., Senker J., Dinnebier R. E., Breu J. (2009). Wöhler and Liebig
Revisited: 176 Years of Polymorphism in
Benzamide - and the Story Still Continues!. Cryst. Growth Des..

[69] Thun J., Seyfarth L., Senker J., Dinnebier R. E., Breu J. (2007). Polymorphism in Benzamide: Solving
a 175-Year-Old Riddle. Angew. Chem., Int. Ed..

[70] Blagden N., Davey R., Dent G., Song M., David W. I. F., Pulham C. R., Shankland K. (2005). Woehler and Liebig Revisited: A Small
Molecule Reveals Its Secrets - The Crystal Structure of the Unstable
Polymorph of Benzamide Solved after 173 Years. Cryst. Growth Des..

[71] Fucke K., McIntyre G. J., Wilkinson C., Henry M., Howard J. A. K., Steed J. W. (2012). New Insights into
an Old Molecule: Interaction Energies
of Theophylline Crystal Forms. Cryst. Growth
Des..

[72] Pinon A. C., Rossini A. J., Widdifield C. M., Gajan D., Emsley L. (2015). Polymorphs
of Theophylline Characterized by DNP Enhanced Solid-State NMR. Mol. Pharmaceutics.

[73] Dyba A. J., Wiącek E., Nowak M., Janczak J., Nartowski K. P., Braun D. E. (2023). Metronidazole Cocrystal Polymorphs with Gallic and
Gentisic Acid Accessed through Slurry, Atomization Techniques, and
Thermal Methods. Cryst. Growth Des..

[74] Reichardt C. (1994). Solvatochromic
Dyes as Solvent Polarity Indicators. Chem. Rev..

[75] Hughes C. E., Williams P. A., Kariuki B. M., Harris K. D. M. (2018). Establishing
the Transitory Existence of Amorphous Phases in Crystallization Pathways
by the CLASSIC NMR Technique. ChemPhysChem.

[76] Urakaev F. Kh., Boldyrev V. V. (2000). Mechanism and Kinetics of Mechanochemical Processes
in Comminuting Devices. Powder Technol..

[77] Hughes C. E., Harris K. D. M. (2008). A Technique for In Situ Monitoring
of Crystallization
from Solution by Solid-State ^13^C CPMAS NMR Spectroscopy. J. Phys. Chem. A.

[78] Pindelska E., Sokal A., Szeleszczuk L., Pisklak D. M., Kolodziejski W. (2014). Solid-State
NMR Studies of Theophylline Co-Crystals with Dicarboxylic Acids. J. Pharm. Biomed. Anal..

[79] Bernard G. M., Goyal A., Miskolzie M., McKay R., Wu Q., Wasylishen R. E., Michaelis V. K. (2017). Methylammonium Lead Chloride: A Sensitive
Sample for an Accurate NMR Thermometer. J. Magn.
Reson..

[80] Ouyang J., Zhou L., Liu Z., Xiao S., Huang X., Heng J. Y. Y. (2019). Solubility Determination and Modelling of Benzamide
in Organic Solvents at Temperatures from 283.15 and 323.15 K, and
Ternary Phase Diagrams of Benzamide-Benzoic Acid Cocrystals in Ethanol
at 298.15 K. J. Mol. Liq..

[81] Fischer F., Scholz G., Benemann S., Rademann K., Emmerling F. (2014). Evaluation
of the Formation Pathways of Cocrystal Polymorphs in Liquid-Assisted
Syntheses. CrystEngComm.

[82] Trask A. V., Shan N., Motherwell W. D. S., Jones W., Feng S., Tan R. B. H., Carpenter K. J. (2005). Selective
Polymorph Transformation
via Solvent-Drop Grinding. Chem. Commun..

[83] Skala M. E., Zeitler S. M., Golder M. R. (2024). Liquid-Assisted Grinding Enables
a Direct Mechanochemical Functionalization of Polystyrene Waste. Chem. Sci..

