# Supplementary material for: Unveiling Solvent-Mediated Mechanochemical Cocrystallization Pathways by In Situ CLASSIC NMR Spectroscopy
Source: Mol Pharm. 2026 Apr 27;23(6):3309–27. doi: 10.1021/acs.molpharmaceut.5c01909 (PMC13231416; doi:10.1021/acs.molpharmaceut.5c01909)
Supplement: Supplementary file 1 [file mp5c01909_si_001.pdf]

Supplementary Information

Unveiling Solvent-Mediated Mechanochemical Cocrystallisation Pathways by *In-Situ* CLASSIC NMR Spectroscopy

Anna M. Gołkowska[1,*], Maciej Nowak[1,*], László Fábián[2], Dinu Iuga[3], Franziska Emmerling[4], †Karol P. Nartowski[1,2], Bożena Karolewicz[1], Yaroslav Z. Khimyak[2,*]

[1] Department of Drug Form Technology, Wroclaw Medical University, Borowska 211A, 50-556 Wroclaw, Poland

[2] School of Chemistry, Pharmacy and Pharmacology, University of East Anglia, Norwich Research Park, Norwich NR4 7TJ, United Kingdom

[3] Department of Physics, University of Warwick, CV4 7AL Coventry, United Kingdom

[4] Federal Institute for Materials Research and Testing (BAM), Richard-Willstätter-Strasse 11, 12489 Berlin, Germany

† Deceased author (25th March 2023)

* Corresponding authors: Y.Khimyak@uea.ac.uk, a.golkowska@umw.edu.pl

Table of contents

1 Powder X-ray Diffraction (PXRD) 3

2 Nuclear Magnetic Resonance (NMR) peaks assignment 4

3 Solubility studies 14

4 Individual components 16

4.1 LAG-induced phase transformations 16

4.2 MAS-induced phase transformations (CLASSIC NMR – control experiments) 17

4.2.1 TP 17

4.2.2 BZ 21

4.2.3 MNZ 23

4.2.4 GAL 24

5 Cocrystallisation studies (CLASSIC NMR) 26

5.1 TP:BZ - MeOD 26

5.2 TP:BZ – TOL_d 27

5.3 MNZ:GAL – D2O 29

5.4 MNZ:GAL – MeOD 32

5.5 MNZ:GAL - TOL_d 34

5.6 Representative ^{1}H NMR spectra (H1 position) 35

6 References 36

1 Powder X-ray Diffraction (PXRD)

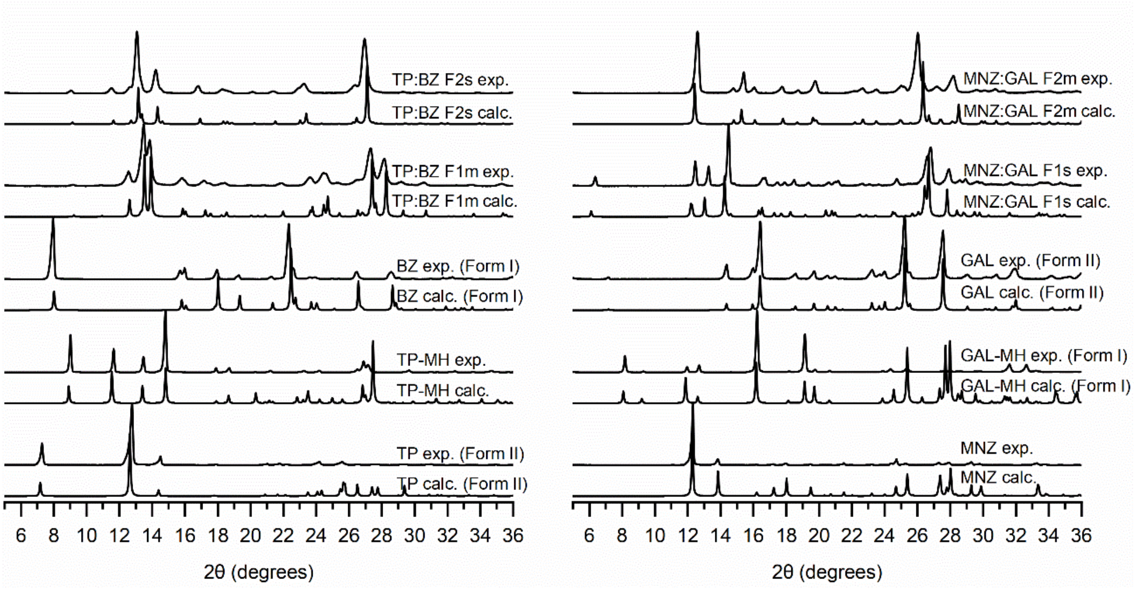

Figure S1. Experimental and calculated PXRD patterns of starting materials (TP, TP-MH, BZ, MNZ, GAL-MH, GAL) and products of ball milling procedures (TP:BZ F1m, TP:BZ F2s, MNZ:GAL F1s, MNZ:GAL F2m). The CSD refcodes used for calculating PXRD patterns: TP (CSD refcode: BAPLOT01),[1] TP-MH (CSD refcode: THEOPH06),[2] BZ (CSD refcode: BZAMID01),[3] TP:BZ F1m (CSD refcode: RABXIE02),[4] TP:BZ F2s (CSD refcode: RABXIE01),[5] MNZ (CSD refcode: MNIMET),[6] GAL (CSD refcode: IJUMEG05),[7] GAL-MH (CSD refcode: KONTIQ01),[8] MNZ:GAL F1s (CSD refcode: VOKYEC),[9] MNZ:GAL F2m (CSD refcode: VOKYEC01)[10].

Among the individual components (*i.e.* TP, BZ, MNZ, GAL), MNZ is the only monomorphic compound.[11] TP, BZ and GAL exhibit polymorphism and, based on PXRD measurements, structures deposited in the CSD (Cambridge Structural Database) and literature data,[7,12–15] the starting materials were identified as TP Form II, BZ Form I and GAL Form II. These reagents were used as starting materials for further NMR studies and grinding experiments without purification.

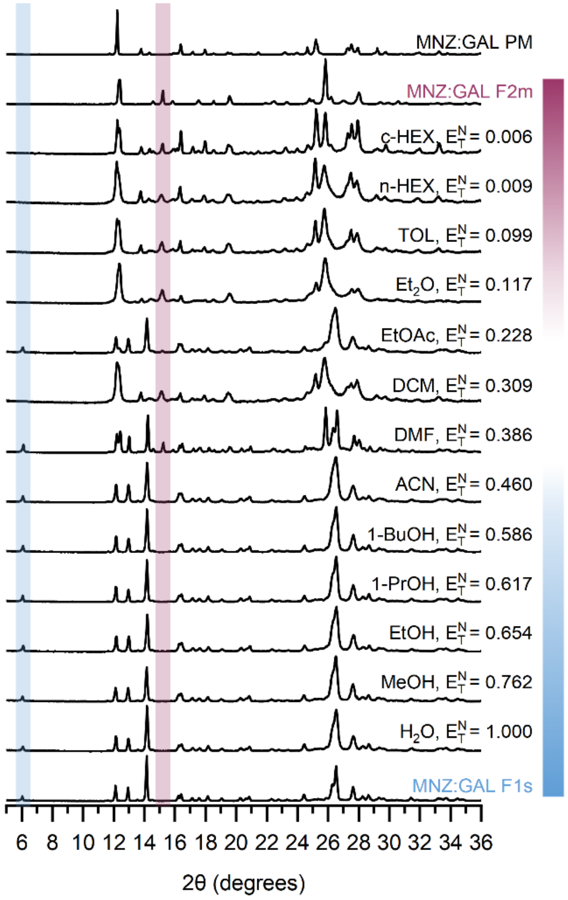

Figure S2. PXRD patterns showing a polymorphic screening of the MNZ:GAL system using LAG method with solvents of different polarity. The results have been arranged according to the increasing values of the normalised solvent polarity (E_T^N).[16]

2 Nuclear Magnetic Resonance (NMR) peaks assignment

The assignment of ^{1}H and ^{13}C NMR peaks of APIs (TP, MNZ), coformers (BZ, GAL), their hydrated forms (TP-MH, GAL-MH) and cocrystals polymorphs (TP:BZ F1m, TP:BZ F2s, MNZ:GAL F1s, MNZ:GAL F2m) was completed based on CASTEP calculated isotropic chemical shifts (**Table S1**, **Table S2**, **Table S3**, **Table S4**) and literature data.[4,5,15,17–25] The assignment is presented in the **Figure 4** (TP:BZ system), **Figure 5** (MNZ:GAL system) and **Figure S4.**

In the ^{1}H NMR spectra, since most of the aromatic lines overlap, the most prominent spectral region revealing the cocrystallisation process is the deshielded peak of (i) H1 (or H1 and H1’) in TP (TP:BZ system) and (ii) H10 in GAL (MNZ:GAL system). H1 is assigned to the proton bound to the nitrogen atom of the imidazole ring in TP and H10 is assigned to the proton of the carboxyl group in GAL. In neat TP, H1 forms a hydrogen-bond with N4 of the adjacent TP molecule (1.924 Å). In the cocrystal structure, this interaction is replaced by an H-bond between H1 (H1’) and O3 (O3’) of the amide group of BZ (F1m: 1. 975, 1.923 Å; F2s: 1.855 Å). Consequently, we observe an upfield shift of the H1 signal

in the ^{1}H NMR spectrum from 14.1 ppm (neat TP or TP:BZ PM) to 13.2 ppm (TP:BZ F2s) or 12.8 ppm (TP:BZ F1m) once the cocrystal is formed (**Figure 4C**). This is in agreement with the pattern observed for the CASTEP calculated ^{1}H chemical shifts changing from 15.1 ppm (TP) to 14.4 ppm (TP:BZ F2s) or 13.7 ppm (TP:BZ F1m). In contrast, in the MNZ:GAL system a down-field shift is observed upon cocrystallisation. In neat GAL, H10 is hydrogen-bonded to O5 of a carboxyl group of another GAL molecule (1.692 Å). When the cocrystal is formed, this H-bond is substituted with the interaction between H10 and N2 of MNZ (F1s: 1.480 Å; F2m: 1.650 Å). Simultaneously, this is reflected in a low-field shift from 12.4 ppm (GAL or MNZ:GAL PM) to 13.8 ppm (MNZ:GAL F2m) or 13.9 ppm (MNZ:GAL F1s) (**Figure 5C**). This corresponds to the changes observed for the CASTEP calculated ^{1}H chemical shifts, *i.e.* a shift from 13.5 ppm (GAL) to 14.2 ppm (MNZ:GAL F2m) or 14.5 ppm (MNZ:GAL F1s). At the same time, as mentioned above, TP and GAL can incorporate water into their structure producing monohydrates. When considering hydrated TP and GAL, the signals of H1 and H10 are not as pronounced as in anhydrous materials. Both H1 and H10 are acidic and exchangeable protons. Our *in-situ* NMR studies used deuterated (^{2}H) solvents and the addition of deuterium oxide (D_2O) resulted in the substitution of ^{1}H for ^{2}H at these positions, thus they could not be observed in ^{1}H NMR measurements (**Figure S3**). It also means that the signal intensity of H1 and H10 in TP-MH and GAL-MH results from the level of deuteration of the solvent used.

The $^{13}C\{^1H\}$ NMR spectra were recorded with a short recycle delay (**Table 1**, **Figure 2**) meaning that under the given experimental conditions the observed signals originate from mobile species. In our systems, these correspond to the methyl groups of TP and MNZ (two and one CH_3 groups, respectively), which act as freely rotating moieties (**Figure 4A**, **Figure 5A**). The shape and position of the resulting peaks depend on the molecular environment of the CH_3 groups, producing distinct resonances for the neat compounds, hydrates, and cocrystal polymorphs. Consequently, the cocrystallisation process can be monitored effectively by tracking spectral changes in the 35 – 10 ppm region (**Figure 4D**, **Figure 4F, Figure 5D, Figure 5F**). The assignment of the ^{13}C peaks of neat TP was based on both CASTEP calculations and literature data.[15,17,19–23] In the ^{13}C spectra, two peaks arising from each of the methyl groups exhibit very similar chemical shifts (C6: 30.1 ppm; C7: 29.8 ppm). The small difference of *ca.* 0.3 ppm might result from the fact that both C6 and C7 are bonded to electronegative nitrogen atoms (N1 and N2, respectively), however, C6 is located between two oxygen atoms (O1 and O2), whereas C7 is adjacent to only one (O1). Upon hydration of TP to form TP-MH, the incorporation of water into the crystal structure causes a reversal in the positions of the C6 and C7 resonances (C6: 28.7 ppm; C7: 30.9 ppm). Cocrystallisation to form TP:BZ F2s does not affect the position of C7 resonance (TP: 29.8 ppm; TP:BZ F2s: 29.8 ppm). In contrast, the C6 signal shifts from 30.1 ppm in neat TP to a noticeably higher frequency of 30.8 ppm in TP:BZ F2s. In neat TP, C6 is linearly positioned between N1, to which

the CH_3 group is bonded, and the O1 atom of an adjacent TP molecule (C6 to O1: 3.155 Å). Upon formation of TP:BZ F2s, this distance increases to 3.629 Å, accompanied by change in interaction geometry, which becomes non-linear. As a result, the deshielding effect of N1 is enhanced, leading to the downfield shift of the C6 resonance in TP:BZ F2s relative to neat TP. The second cocrystal polymorph, TP:BZ F1m contains two symmetry-independent TP molecules in the asymmetric unit (Z' = 2) (**Figure S4A**), each giving rise to its own set of peaks. Therefore, we expected four distinct peaks produced by each CH_3 group. Consequently, four distinct signals from the CH_3 groups were expected. However, only three peaks are observed in the methyl region of the NMR spectra, as the C6 and C7 resonances overlap at 30.5 ppm, producing a peak of doubled intensity, while C6' (33.1 ppm) and C7' (31.9 ppm) remain distinct and resonate at higher frequencies. In contrast to the TP:BZ system, in MNZ:GAL possesses a single CH_3 group and all structures crystallise in the Z' = 1 units only. Consequently, a single resonance at 15.2, 14.1 or 13.4 ppm enables identification of MNZ, MNZ:GAL F2m, and MNZ:GAL F1s, respectively. Because MNZ is a more flexible molecule than TP — owing to the presence of an aliphatic region — its spectra are more sensitive to intramolecular geometry. The distance between C4 and the O3 atom of the hydroxyl group appears to play a key role in determining the C4 chemical shift. This distance measures 3.444 Å in MNZ, 3.538 Å in MNZ:GAL F2m, and 3.860 Å in MNZ:GAL F1s, consistent with the observed upfield shift of the C4 resonance. It is worth nothing that while methyl region allows monitoring of cocrystallisation in both systems hydrate formation can be detected only in the TP:BZ system, as GAL-MH lacks mobile moieties in its structure. The peaks of BZ and GAL, as well as those of TP and MNZ other than CH_3 groups, are not visible in the $^{13}C\{^1H\}$ NMR spectra (**Figure 4D**, **Figure 5D**) and appear only when the compounds are dissolved in the investigated solvent (**Figure 3**). The ^{13}C-rich solvents also produce distinct signals in the $^{13}C\{^1H\}$ NMR spectra (MeOD: 48 ppm; TOL_d: 137, 128, 125 and 20 ppm) (**Figure 3**).

In the ^1H-^{13}C CP/MAS NMR spectra, the methyl groups peaks also serve as an indicator of phase changes. Additional resonances arising from TP, MNZ, BZ and GAL in the spectral region of 180 – 40 ppm facilitate phase identification, allowing the determination of the identity of the reaction product and/or the remaining residue of the starting materials.

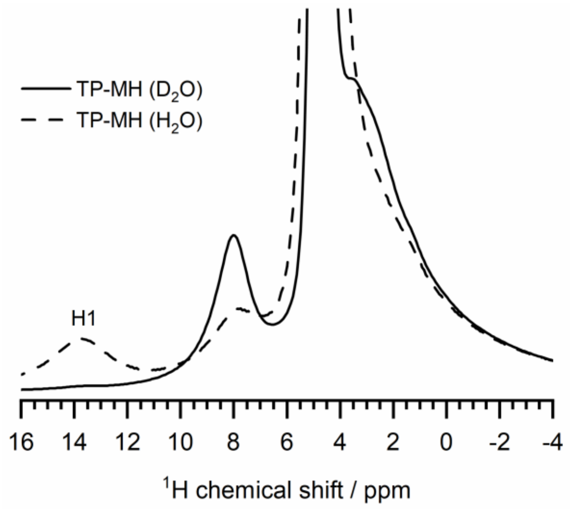

Figure S3. ^{1}H NMR spectra of TP-MH prepared with water (H_2O) and deuterium oxide (D_2O) presenting the behaviour of H1 (acidic, exchangeable proton) in the presence of the ^{1}H- and ^{2}H-rich solvents.

Table S1. Experimental and CASTEP calculated ^{13}C chemical shifts of the reference materials for TP:BZ system.

	TP exp.	TP calc.	TP-MH exp.	TP-MH calc.	TP:BZ F1m exp.	TP:BZ F1m calc.	TP:BZ F2s exp.	TP:BZ F2s calc.	BZ exp.	BZ calc.
RMSD	1.6879		1.9812		1.7335		1.5903		1.3882	
C1	150.8	150.28	150.3	148.38	148.5	149.18	151.3	149.88		
C2	146.1	146.48	148.4	147.78	150.4[a]	149.58	147.7	148.08		
C3	105.9	108.48	106.5	108.88	108.1[a]	110.78	108.0	109.98		
C4	155.1	154.18	156.0	153.48	156.2	154.68	156.8	155.28		
C5	140.8	140.38	142.6	142.58	139.2	139.38	140.3	140.58		
C6	30.1	28.78	28.7	27.18	30.5[a]	29.78	30.8	28.58		
C7	29.8	26.58	30.9	27.88	30.5	28.28	29.8	27.78		
C8					131.9[a]	134.48	133.1	135.08	129.8[a]	131.68
C9					127.5[a]	129.18	126.6[a]	127.98	129.8	129.88
C10					126.4[a]	128.28	126.6	128.68	129.8	130.48
C11					131.9	133.88	132.1	133.08	135.2	137.08
C12					124.6	126.68	125.6	126.98	129.8	130.88
C13					129.1	130.98	128.7	130.88	129.8	131.68
C14					167.5	166.78	170.0	169.68	173.3	174.28
C1’					150.4	149.18				
C2’					148.1	148.68				
C3’					108.1	110.18				
C4’					156.7	155.58				
C5’					140.8	140.58				
C6’					33.1	30.48				
C7’					31.9	30.08				
C8’					131.9	133.78				
C9’					127.5	129.78				
C10’					127.5	129.18				
C11’					131.9	133.78				
C12’					126.4 (127.5)[b]	129.08				
C13’					126.4	127.98				
C14’					169.4	168.88				

[a] Due to peak overlap in the experimental spectra, multiple calculated shieldings were assigned to a single observed peak (for details see Experimental Section: Computational Details)

[b] In cases of computational uncertainty, alternative assignments are provided in brackets

Table S2. Experimental and CASTEP calculated ^{1}H chemical shifts of the reference materials for TP:BZ system.

	TP exp.	TP calc.	TP-MH exp.	TP-MH calc.	TP:BZ F1m exp.	TP:BZ F1m calc.	TP:BZ F2s exp.	TP:BZ F2s calc.	BZ exp.	BZ calc.
RMSD	0.4575		1.0869		1.0209		0.9067		1.3998	
H1	14.1	15.12	13.2	14.82	12.8[a]	13.72	13.2	14.42		
H2	7.5	7.02	7.5[a]	7.22	7.2[a]	8.02	7.2	7.12		
H3	3.1[a]	2.82[b]	3.0[a]	2.32[b]	3.1[a]	2.68[b]	3.1[a]	2.78[b]		
H4	3.1	2.82	3.0	2.32	3.1	2.68	3.1	2.78		
H5	3.1	2.82	3.0	2.32	3.1	2.68	3.1	2.78		
H6	3.1	2.88[b]	3.0	2.65[b]	3.1	2.85[b]	3.1	2.28[b]		
H7	3.1	2.88	3.0	2.65	3.1	2.85	3.1	2.28		
H8	3.1	2.88	3.0	2.65	3.1	2.85	3.1	2.28		
H9					7.2[a]	6.52	7.2[a]	6.92	7.0[a]	6.22
H10					7.2	5.92	7.2	6.02	7.0	4.82
H11					7.2	6.92	7.2	6.52	7.0	5.72
H12					7.2	6.92	7.2	6.32	7.0	6.52
H13					7.2	6.72	7.2	6.32	7.0	5.72
H14					7.2	8.32	7.2	8.32	7.0	8.22
H15					7.2	9.72	7.2	9.12	7.0	8.82
H16			7.5	6.32						
H17			2.6	0.22						
H1’					12.8	14.52				
H2’					7.2	7.52				
H3’					3.1	2.72[b]				
H4’					3.1	2.72				
H5’					3.1	2.72				
H6’					3.1	2.85[b]				
H7’					3.1	2.85				
H8’					3.1	2.85				
H9’					7.2	6.22				
H10’					7.2	6.12				
H11’					7.2	7.02				
H12’					7.2	7.12				
H13’					7.2	6.32				

H14’	7.2	10.52
H15’	7.2	8.42

[a] Due to peak overlap in the experimental spectra, multiple calculated shieldings were assigned to a single observed peak (for details see for details see Experimental Section: Computational Details)

[b] Due to rapid rotational averaging of the TP methyl groups, the three equivalent protons of each CH_3 moiety were treated as a single entity, resulting in one σ_{calc} value per methyl group (for details see *Experimental Section, Computational Details*)

Table S3. Experimental and CASTEP calculated ^{13}C chemical shifts of the reference materials for MNZ:GAL system.

	MNZ exp.	MNZ calc.	MNZ:GAL F1s exp.	MNZ:GAL F1s calc.	MNZ:GAL F2m exp.	MNZ:GAL F2m calc.	GAL exp.	GAL calc.	GAL-MH exp.	GAL-MH calc.
RMSD	2.2909		2.1900		2.1009		1.6320		1.4361	
C1	153.6	155.04	151.4	152.14	151.9	153.54				
C2	131.6	134.94	132.1	134.84	132.6	136.24				
C3	138.2	139.94	137.1	138.64	136.9	137.84				
C4	15.2	11.74[c]	13.4	8.74[c]	14.1	9.74[c]				
C5	51.2	49.34	51.2	49.34	51.7	50.54				
C6	59.6	59.94	60.9	60.94	61.5	61.44				
C7			123.3	120.34	123.0	120.74	118.0	115.74	121.4	119.74
C8			109.4[a]	106.44	108.7	106.14	110.7	108.94	110.2[a]	109.74
C9			145.7	146.84	145.7	147.64	143.0[a]	144.34	141.7	143.24
C10			135.6	137.84	135.1	136.54	140.9 (143.0)[b]	143.44	135.2	137.64
C11			145.0	146.34	143.2	144.44	143.0 (140.9)[b]	143.04	143.2	144.84
C12			109.4	108.24	109.8	108.54	113.1	111.64	110.2	109.74
C13			169.6	169.44	170.1	170.14	174.8	174.74	169.0	169.04

[a] Due to peak overlap in the experimental spectra, multiple calculated shieldings were assigned to a single observed peak (for details see for details see Experimental Section: Computational Details)

[b] In cases of computational uncertainty, alternative assignments are provided in brackets

[c] Significant deviation between experimental and calculated shifts (CH_3 of MNZ)

Table S4. Experimental and CASTEP calculated ^{1}H chemical shifts of the reference materials for MNZ:GAL system.

	MNZ exp.	MNZ calc.	MNZ:GAL F1s exp.	MNZ:GAL F1s calc.	MNZ:GAL F2m exp.	MNZ:GAL F2m calc.	GAL exp.	GAL calc.	GAL-MH exp.	GAL-MH calc.
RMSD	0.6767		1.8390		2.1273		0.6677		1.3160	
H1	7.9[a]	8.08	6.9[a]	7.28	7.4[a]	7.48				
H2	2.2[a]	1.71[b]	2.0[a]	1.54[b]	2.5[a]	1.61[b]				
H3	2.2	1.71	2.0	1.54	2.5	1.61				
H4	2.2	1.71	2.0	1.54	2.5	1.61				
H5	4.3[a]	3.98	6.9 (2.0)[c]	4.98	7.4 (2.5)[c]	5.08				
H6	4.3	3.48	6.9 (2.0)[c]	3.68	7.4 (2.5)[c]	3.58				
H7	4.3	3.58	6.9 (2.0)[c]	4.28	7.4 (2.5)[c]	3.88				
H8	4.3	4.18	6.9 (2.0)[c]	4.48	7.4 (2.5)[c]	4.08				
H9	7.9	6.48	6.9	5.38	7.4	6.18				
H10			14.0	14.48	13.9	14.18	12.4	13.48	11.7	12.38
H11			6.9	6.18	7.4	6.08	6.6[a]	6.68	6.3[a]	6.58
H12			6.9	6.88	7.4	10.88	8.2[a]	9.18	7.1[a]	9.38
H13			6.9	10.98	7.4	9.18	5.4	5.08	7.1	8.18
H14			6.9	8.88	7.4	9.28	8.2	7.78	7.1	9.28
H15			6.9	6.68	7.4	6.48	6.6	5.98	6.3	5.98
H16									-[d]	-
H17									6.3	7.18

[a] Due to peak overlap in the experimental spectra, multiple calculated shieldings were assigned to a single observed peak (for details see for details see Experimental Section: Computational Details)

[b] Due to rapid rotational averaging of the TP methyl groups, the three equivalent protons of each CH_3 moiety were treated as a single entity, resulting in one σ_{calc} value per methyl group (for details see *Experimental Section, Computational Details*)

[c] In cases of computational uncertainty, alternative assignments are provided in brackets

[d] H16 peak excluded due to overlap with residual water signal (GAL-MH)

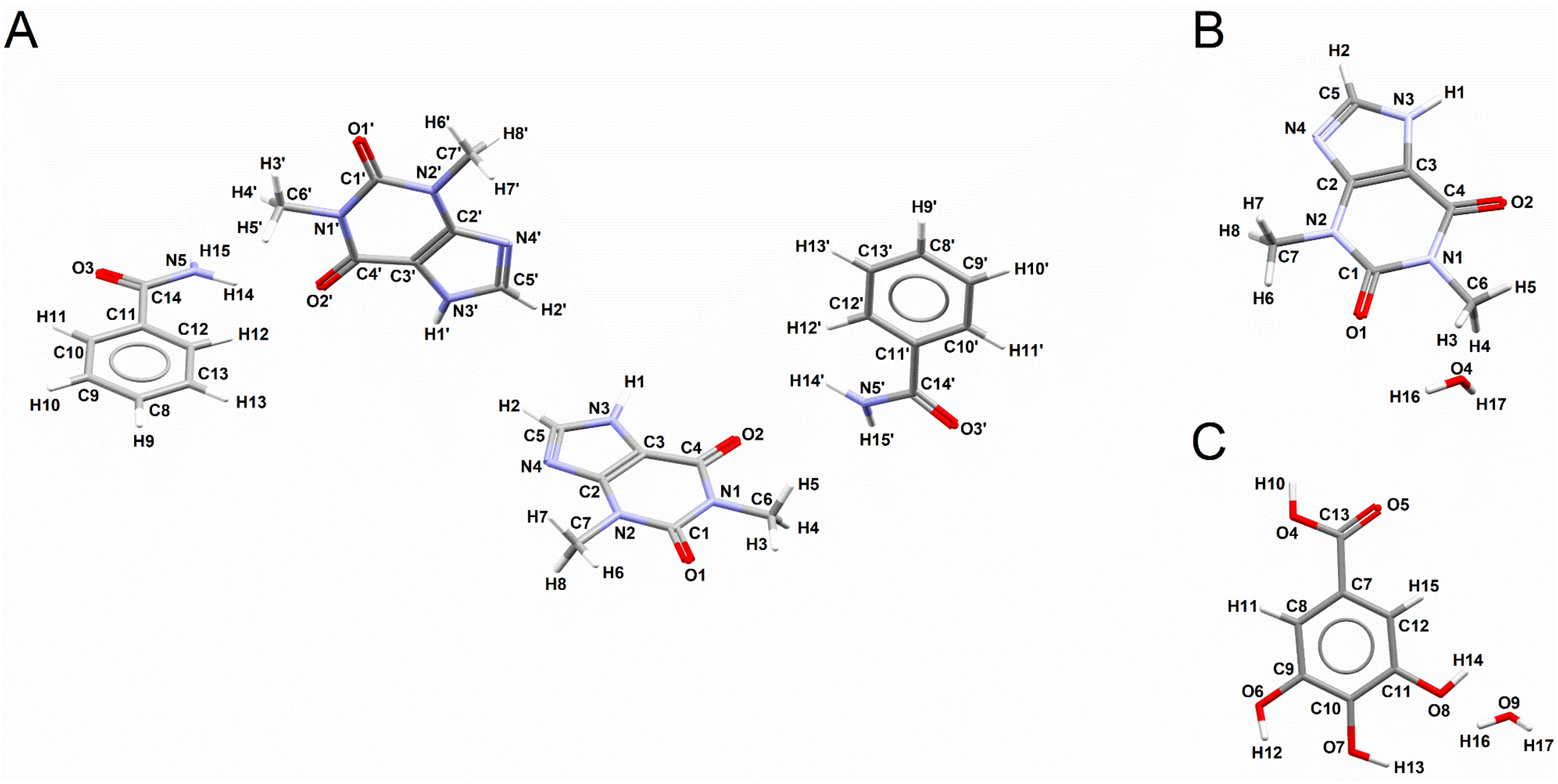

Figure S4. A) An asymmetric unit of TP:BZ F1m; B) a molecular structure of TP-HM with atom labelling; C) a molecular structure of GAL-HM with atom labelling.

3 Solubility studies

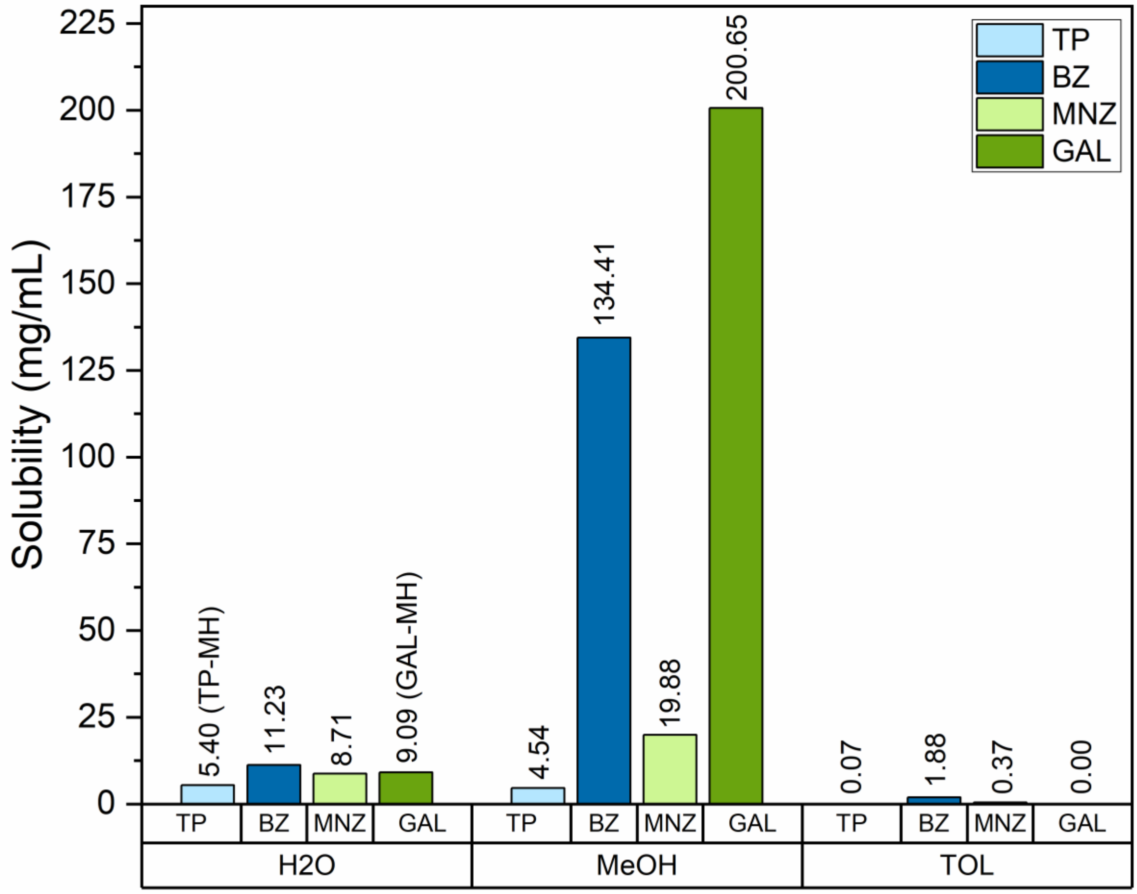

Figure S5. The solubility of TP, BZ, MNZ and GAL in H_2O, MeOH and TOL (mg mL^{-1}) determined by HPLC analysis.

Saturated solutions for solubility determination were prepared by suspending compounds in the solvents of choice and stirring at RT for 24 h. The obtained filtrates were diluted if needed and subjected to HPLC analysis. The remaining powders were measured using PXRD to address the possibility of solvent-mediated phase transitions which could occur during stirring.

The majority of the samples did not undergo any phase transformation. The exceptions were TP in water and GAL in water which immediately converted into TP-MH and GAL-MH, respectively. Therefore, the determined solubility of these compounds refers to TP-MH and GAL-MH in water, as shown in the (**Figure S5**).

In addition, phase transitions of TP in MeOH and GAL in MeOH were observed after 24 h of stirring. Therefore, the stirring time for the samples was reduced to 2 h and 20 min, respectively. This ensured the saturation of the solutions and prevented phase transformations from occurring before solution withdrawal.

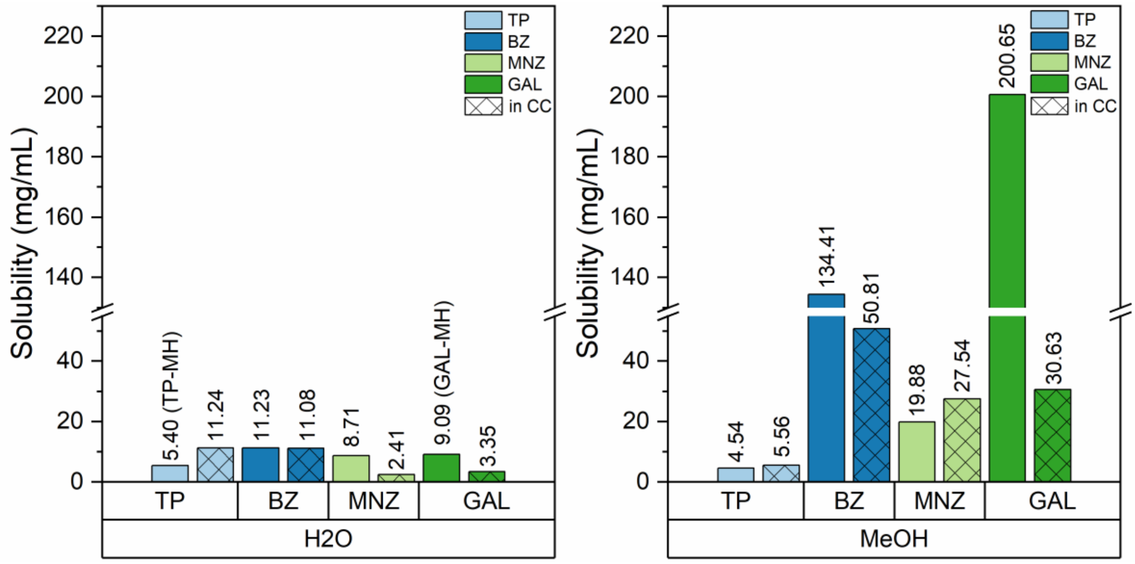

Figure S6. The solubility of TP and BZ in a form of TP:BZ F2s cocrystal (blue bars with pattern) and MNZ and GAL in a form of MNZ:GAL F1s cocrystal (green bars with pattern) in H_2O and MeOH (mg mL^{-1}) determined by HPLC analysis. The data were compared with the solubility of neat compounds (bars without pattern).

The established solubility (**Figure S6**) refers to the stable cocrystal polymorphs, *i.e.* TP:BZ F2s and MNZ:GAL F1s. The PXRD measurements confirmed lack of solvent-mediated transition and after 24 h of stirring the suspensions the powders remained TP:BZ F2s and MNZ:GAL F1s.

4 Individual components

4.1 LAG-induced phase transformations

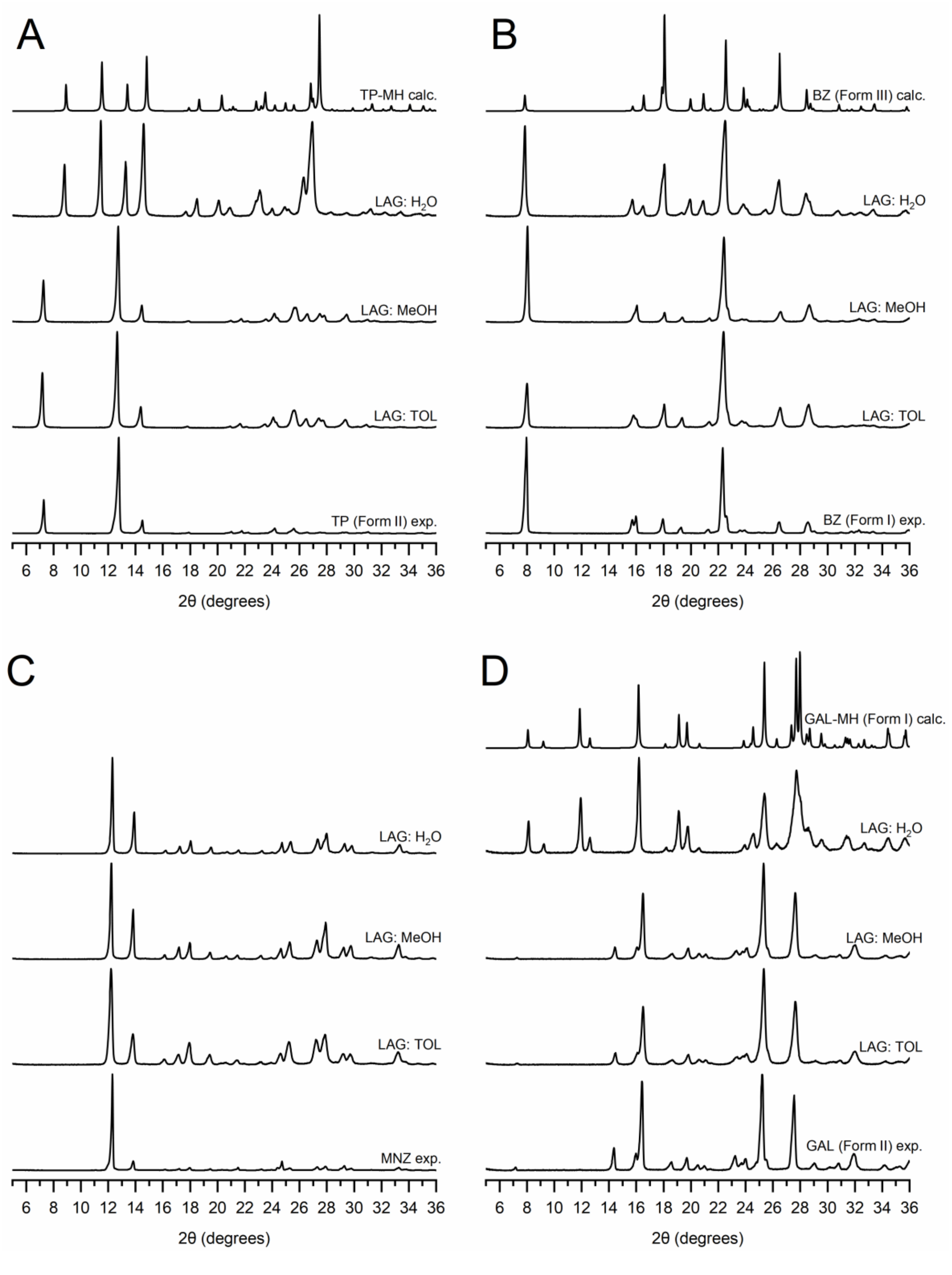

Figure S7. Experimental PXRD patterns of the products of LAG procedures compared with the experimental PXRD patterns of the starting materials (and calculated PXRD patterns of monohydrates and polymorphs if applicable). Grinding of A) TP, B) BZ, C) MNZ and D) GAL was conducted in the presence of H_2O, MeOH, TOL (η = 0.17 µL mg^{-1}). The CSD refcodes used for calculating PXRD patterns: TP-MH (CSD refcode: THEOPH06),[2] GAL-MH (CSD refcode: KONTIQ01),[8] BZ form III (CSD refcode: BZAMID08).[26]

The possibility of phase transformations of the individual components (TP, BZ, MNZ, GAL) during LAG experiments was evaluated in the presence of the solvents selected for the NMR studies, *i.e.* H_2O, MeOH, TOL (η = 0.17 µL mg^{-1}). The described experiments did not result in phase transitions of the examined compounds (**Figure S7**), with the exception of TP and GAL ground with H_2O, which led to the formation of TP-MH (**Figure S7A**) and GAL-MH (**Figure S7D**) and BZ ground with H_2O, which converted to BZ Form III (**Figure S7B**).

4.2 MAS-induced phase transformations (CLASSIC NMR – control experiments)

The neat components (TP and BZ or MNZ and GAL) were tested using the same experimental setup as their physical mixtures (TP:BZ PM and MNZ:GAL). The CLASSIC NMR studies were conducted in the presence of D_2O and MeOD to evaluate the interaction of each compound with the solvent.

4.2.1 TP

As mentioned in the main manuscript, TP immediately converts into TP-MH upon contact with water (**Figure S7A**) as reflected by changes of the ^1H NMR chemical shift of the H1 site (**Figure S8A**). At the same time, the addition of MeOH does not induce any phase transition of TP (**Figure S7A**) and the position of the H1 peak remains unchanged throughout the TP:MeOD CLASSIC NMR experiment (**Figure S8B**).

In terms of the H1 signal shift in TP:D_2O, the reaction started at 14.06 ppm (the reference value of neat TP: 14.13 ppm) and gradually shifted towards 13.4 ppm over the following 45 minutes. It did not reach the expected H1 proton reference value for TP-MH (13.17 ppm) but it might be due to the fact that the measured sample was still a mixture of TP and TP-MH. According to stoichiometry, the amount of D_2O added to neat TP was sufficient to convert TP into TP-MH. However, incomplete conversion might result from the limited mixing in the rotor.

When the TP:D_2O data were compared with the TP:BZ D_2O data, the peak of H1 in the latter reached 13.15 ppm which is closer to the TP-MH reference value. This could be the consequence of the different TP to D_2O ratios in TP:D_2O (70 mg : 10 µL) and TP:BZ D_2O (41.9 mg : 10 µL). Therefore, this might explain the difference in conversion time which was *ca.* 40 minutes for TP:D_2O and 10 minutes for TP:BZ D_2O.

The transition of TP to TP-MH was confirmed using ^1H-^{13}C CP/MAS NMR measurements (**Figure S8C**). Changes in phase distribution demonstrate an ongoing increase of TP-MH and a corresponding decrease of TP in the analysed sample. At the same time, the TP:MeOD did not exhibit any phase transformation (**Figure S8D**). The $^{13}C\{^1H\}$ NMR data corroborate with the described observations. The reference width of the methyl groups peak(s) is broader for TP-MH (3.08 ppm, 658.49 Hz) compared

to TP (1.17 ppm, 253.67 Hz) which is noticeable in the $^{13}C\{^{1}H\}$ NMR contour maps for TP:D_2O (**Figure S8E**) and TP:MeOD (**Figure S8F**).

Meanwhile, regardless of the solvent used, TP does not enter the liquid phase. The **Figure S9A** and **Figure S9B** demonstrate only signals arising from the methyl groups which appear in the $^{13}C\{^{1}H\}$ NMR spectra due to their short spin-lattice relaxation time. If dissolution were to occur, other peaks assigned to TP (*e.g.* in the 160–135 ppm region) would be visible on the contour plots.

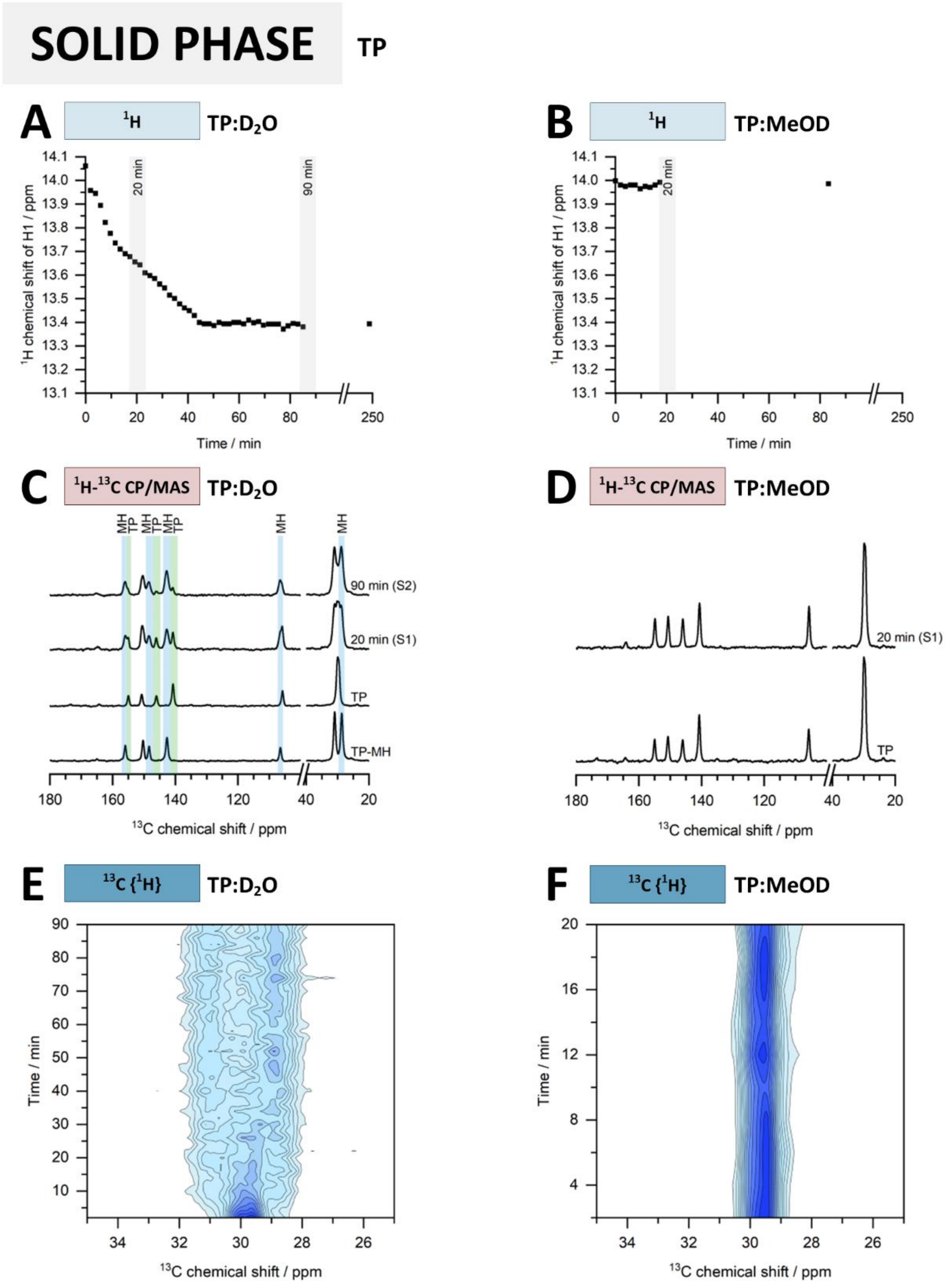

Figure S8. The comparison of the solid phase behaviour of neat TP upon the addition of D_2O and MeOD, monitored with the CLASSIC NMR. The ^{1}H NMR chemical shift (ppm) of the H1 proton of neat TP monitored after the addition of A) D_2O and B) MeOD; The ^{1}H-^{13}C CP/MAS NMR spectra of C) TP:D_2O: 20 – 80 and 90 – 250 minutes and D) TP:MeOD: 20 – 80 minutes into the CLASSIC NMR experiments compared with the reference spectra (TP:BZ PM is marked with green rectangles and TP-MH with light blue rectangles); The intensity contour plot comprising all ^{13}C{^{1}H} NMR spectra recorded as a function of time (methyl groups region 35 – 25 ppm, d_1 = 3 s) for E) TP:D_2O and F) TP:MeOD.

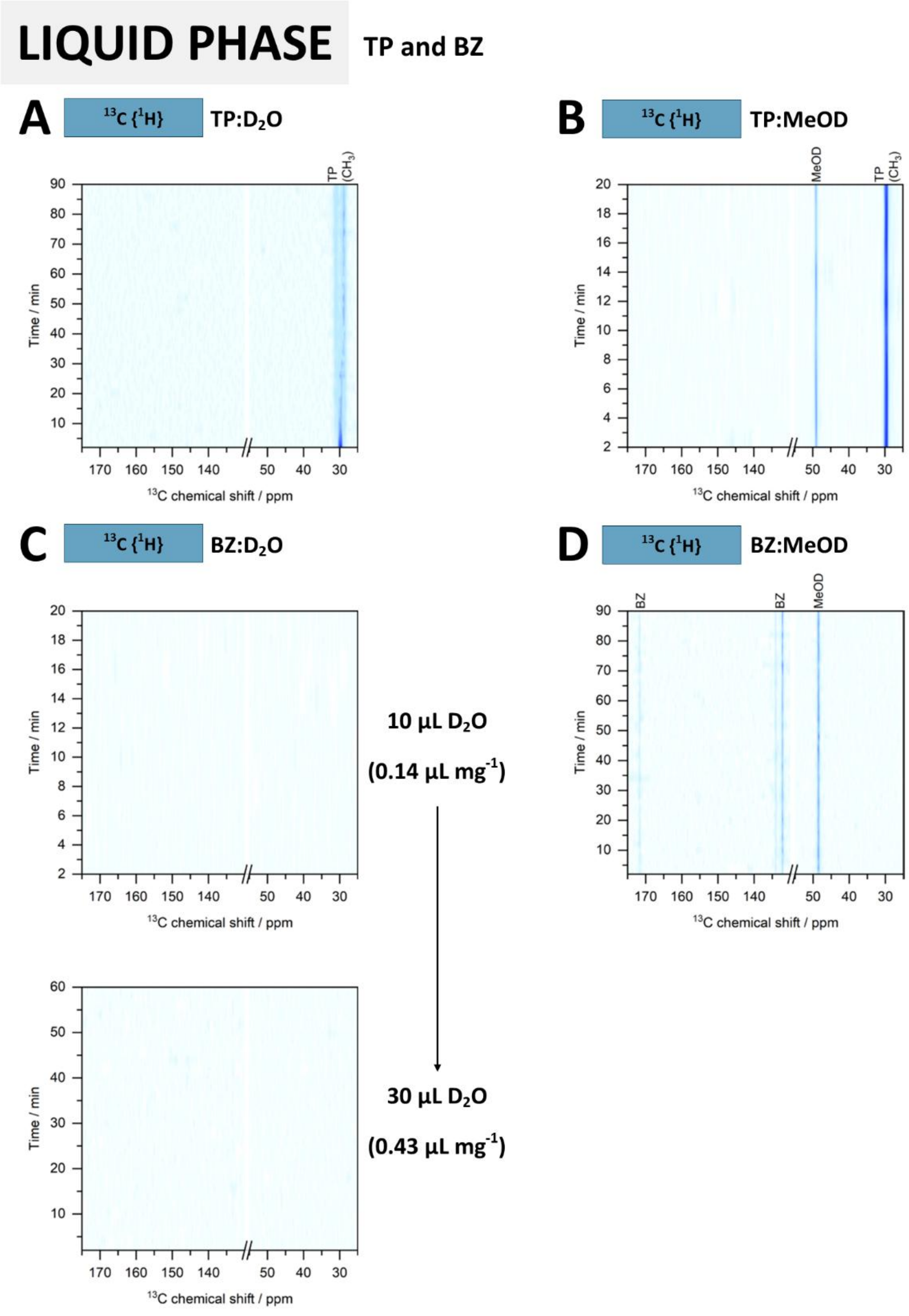

Figure S9. The comparison of the liquid phase behaviour of neat TP and neat BZ upon the addition of D_2O and MeOD, monitored with the CLASSIC NMR. The intensity contour plot comprising all $^{13}C\{^1H\}$ NMR spectra (d_1 = 3 s) recorded as a function of time for A) TP:D_2O, B) TP:MeOD, C) BZ:D_2O (with the addition of 10 µL and 30 µL of D_2O), D) BZ:MeOD.

4.2.2 BZ

Changes in the solid state behaviour of BZ were difficult to track due to its long spin-lattice relaxation time of BZ compared to the relatively short recycle delay that was used during ^{1}H-^{13}C CP/MAS NMR acquisitions. Consequently, BZ peaks were not visible in the recorded spectra. Furthermore, the complexity of the ^{1}H NMR spectra and the absence of any distinct spectral regions prevented the analysis of the solid-phase behaviour of BZ. The possible appearance of the BZ form III, which occurs when BZ is ground in the presence of water (**Figure S7B**), was not confirmed in any of the CLASSIC NMR experiments, including the neat BZ and TP:BZ datasets. During TRIS-PXRD measurements reported by Lampronti *et al.*,[27] the occurrence of BZ form III was also not revealed during cocrystallisation of TP:BZ with water.

Regarding $^{13}C\{^{1}H\}$ NMR measurements (**Figure S9D**), they confirmed dissolution of BZ in MeOD. In contrast, no signal was detected in BZ:D_2O experiments (**Figure S9C**), even when the acquisition was repeated in the presence of an increased amount of D_2O (30 µL) instead of the amount routinely used (10 µL) for CLASSIC NMR studies. This stands in agreement with the established solubility of BZ in MeOH and water (**Figure S5**).

Interestingly, BZ dissolution was observed during the TP:BZ D_2O CLASSIC NMR (**Figure 6E**). This suggests that the solubility of BZ in water increases in the presence of TP. However, HPLC studies revealed no significant influence of TP addition on BZ solubility in water. It is worth noting that the amount of BZ in the solution was evaluated by HPLC after 24 h of stirring, thus after reaching an equilibrium, which might not reflect the BZ solubility observed during CLASSIC NMR studies (up to 90 min).

Nonetheless, during solubility studies, it was noticed that BZ powder had poor water permeability. This led to the hypothesis that the lack of BZ dissolution and subsequent enhancement upon mixing BZ with TP might be due to differences in the water permeability of TP, BZ and their mixture. This hypothesis was verified by measurements conducted using a goniometer (**Figure S10**). As expected, the time needed for a drop of water to go through a powder bed was the longest for the BZ sample, *i.e.* 215 s. In contrast, it took less than 0.5 seconds for a water drop to permeate the TP surface. When the components were mixed at a 1:1 molar ratio, there was no sign of the water drop after 1.5 seconds. The improved water permeability of BZ in the mixture with TP, compared to neat BZ, could explain the dissolution observed in TP:BZ D_2O sample and its lack in BZ:D_2O CLASSIC NMR experiments.

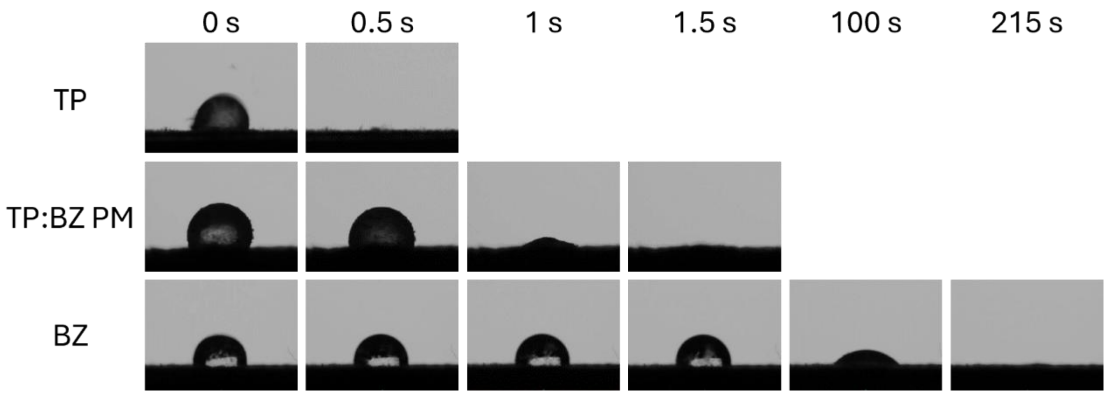

Figure S10. Frames from the films recorded during permeability measurements using a goniometer (a single drop of water placed on the TP, BZ or TP:BZ PM powder bed).

4.2.3 MNZ

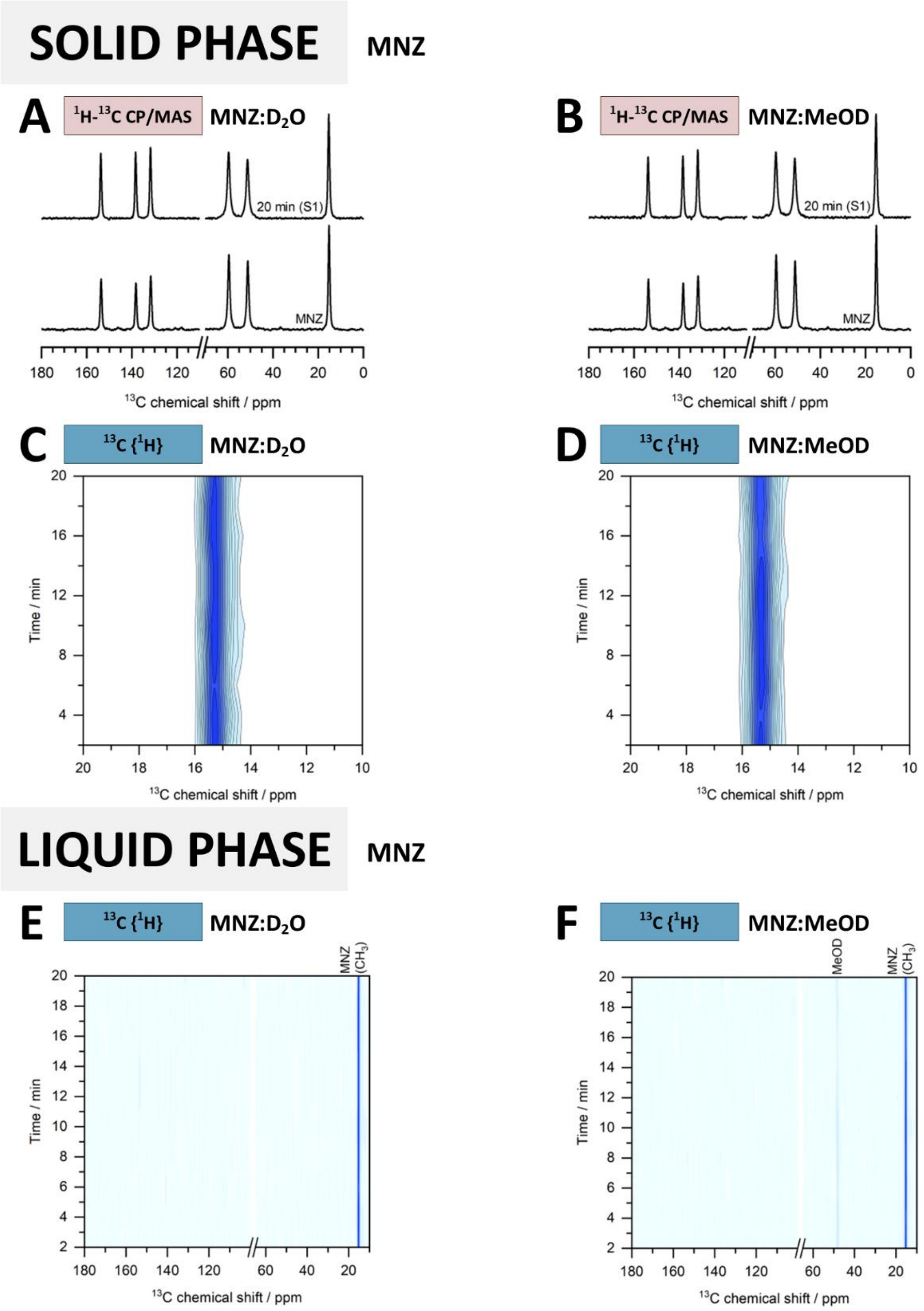

Figure S11. The comparison of the solid phase and liquid phase behaviour of neat MNZ upon the addition of D_2O and MeOD, monitored with the CLASSIC NMR. The ^{1}H-^{13}C CP/MAS NMR spectra (20 – 80 min into the CLASSIC NMR experiments) of A) MNZ:D_2O and B) MNZ:MeOD compared with the reference MNZ spectrum; The intensity contour plot comprising all ^{13}C{^{1}H} NMR spectra (d_1 = 3 s) recorded as a function of time for C) MNZ:D_2O (methyl group region 20 – 10 ppm), D) MNZ:MeOD (methyl group region 20 – 10 ppm), E) MNZ:D_2O and F) MNZ:MeOD.

Neat MNZ did not undergo any phase transitions upon the addition of D_2O and MeOD. This was confirmed by ^{1}H-^{13}C CP/MAS NMR spectra (Figure S11A, Figure S11B), which were in agreement with the neat MNZ spectrum. Additionally, evaluation of the MNZ methyl group position in the $^{13}C\{^{1}H\}$ NMR spectra (Figure S11C, Figure S11D) revealed that it remained unchanged throughout the CLASSIC NMR experiments. At the same time, MNZ did not enter the liquid phase which was expected based on the solubility studies conducted (Figure S5). This was proven in the NMR data based on the $^{13}C\{^{1}H\}$ NMR spectra (Figure S11E, Figure S11F) which lacked MNZ peaks other than those assigned to the MNZ methyl group.

4.2.4 GAL

Similarly to TP, GAL transforms into its hydrated form (GAL-MH) immediately after adding D_2O to the investigated system. This is evident in the ^{1}H-^{13}C CP/MAS NMR data (**Figure S12C**) which show the occurrence of GAL-MH, alongside the remaining traces of unreacted GAL. The same was true for the TP:D_2O sample, for which hydrate formation was incomplete within the timeframe of the experiment. At the same time, MeOD does not induce any phase transition (**Figure S12D**) and the spectrum acquired at the end of the CLASSIC NMR sequence is consistent with the neat, reference GAL. These observations are also reflected in the position of the H10 peak in the ^{1}H NMR spectra, which oscillates around 12.1 ppm for MeOD (**Figure S12B**) and 11.5 ppm for D_2O (**Figure S12A**) while the H10 reference values are 12.4 ppm and 11.7 ppm for GAL and GAL-MH, respectively.

Unlike TP, the GAL structure contains no methyl groups; therefore, the GAL-MH formation process cannot be tracked using $^{13}C\{^{1}H\}$ NMR and these spectra serve as only serve as an indicator of dissolution. For D_2O dataset no signal was detected (**Figure S12E**), confirming that in D_2O the GAL-MH formation occurs instead of GAL dissolution. At the same time, according to the solubility studies (**Figure S5**), GAL was expected to dissolve and appear in the $^{13}C\{^{1}H\}$ NMR spectra after MeOD addition. However, we did not observe this in $^{13}C\{^{1}H\}$ NMR dataset (**Figure S12F**).

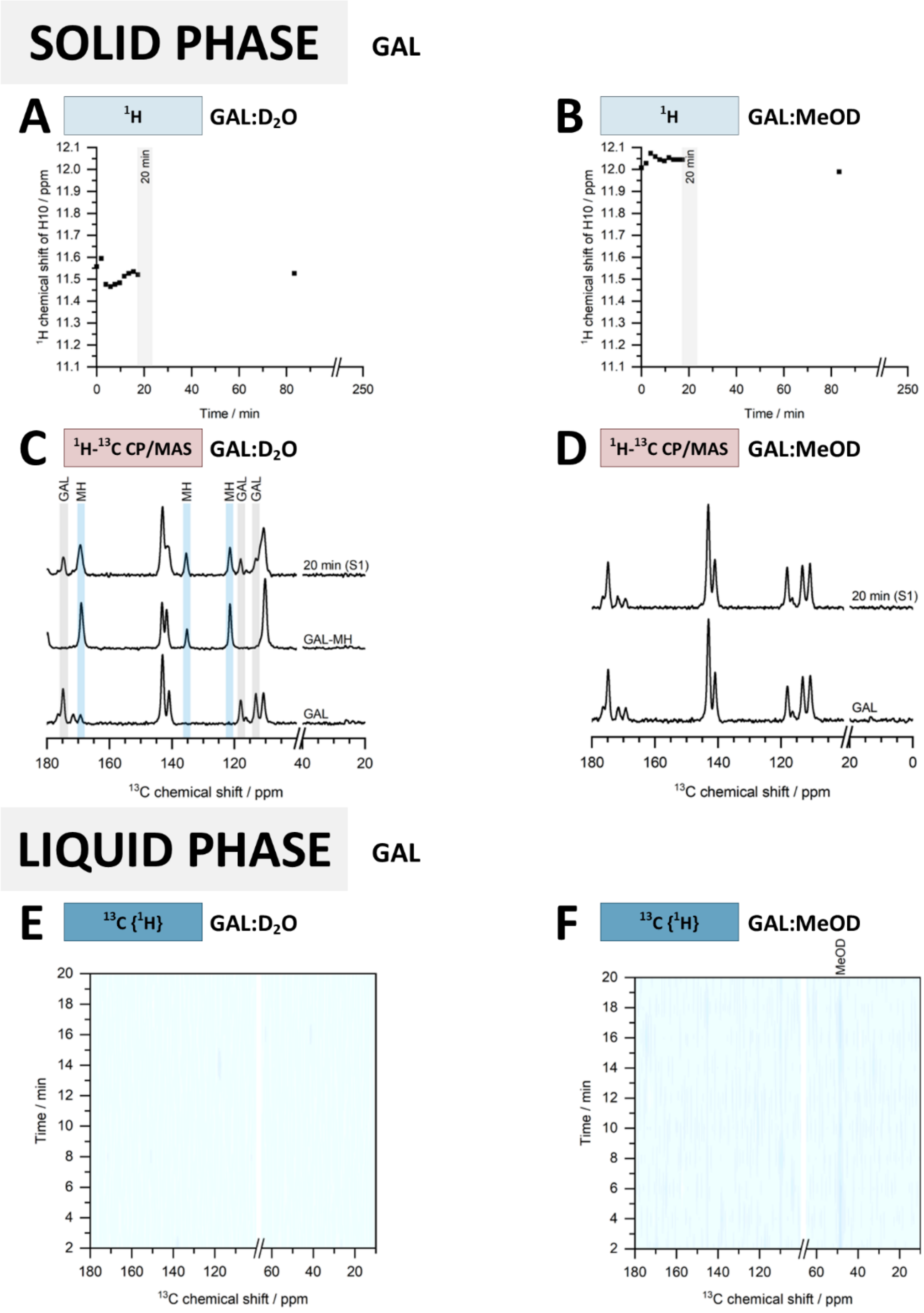

Figure S12. The comparison of the solid phase and liquid phase behaviour of neat GAL upon the addition of D_2O and MeOD, monitored with the CLASSIC NMR. The ^{1}H NMR chemical shift (ppm) of the H10 proton of neat GAL monitored after the addition of A) D_2O and B) MeOD; The ^{1}H-^{13}C CP/MAS NMR spectra (20 – 80 min into the CLASSIC NMR experiments) of C) GAL:D_2O and D) GAL:MeOD compared with the reference spectra (GAL is marked with grey rectangles and GAL-MH with light blue rectangles); The intensity contour plot comprising all $^{13}C\{^{1}H\}$ NMR spectra (d_1 = 3 s) recorded as a function of time for E) GAL:D_2O and F) GAL:MeOD.

5 Cocrystallisation studies (CLASSIC NMR)

5.1 TP:BZ - MeOD

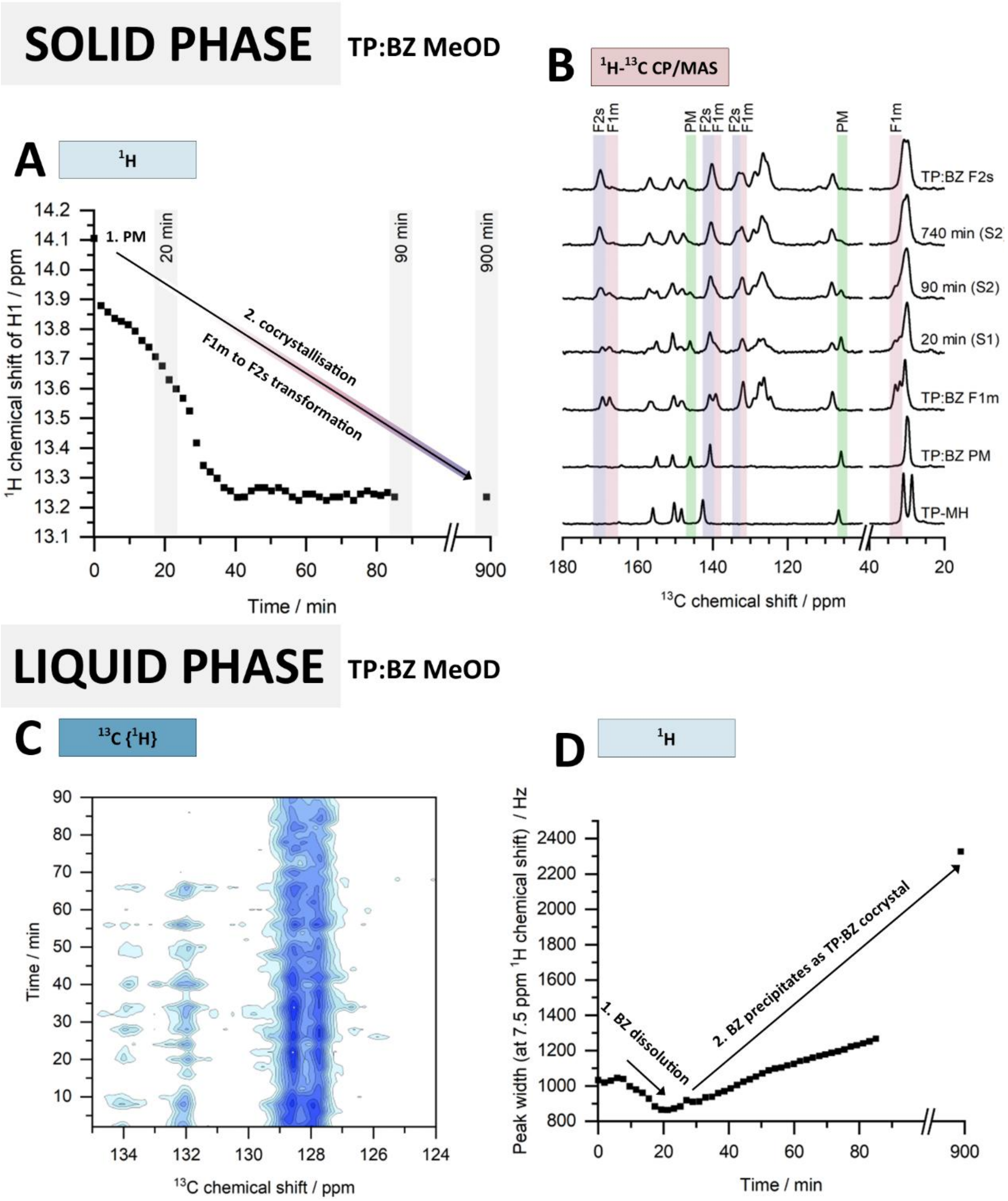

Figure S13. A) The ^{1}H NMR chemical shift (ppm) of the H1 proton of the TP:BZ system monitored during the CLASSIC NMR experiment conducted with the addition of MeOD – for details see **Figure S20**; B) The ^{1}H-^{13}C CP/MAS NMR spectra of TP:BZ MeOD: 20 – 80, 90 – 250 and 740 – 900 minutes into the CLASSIC NMR experiments compared with the reference spectra (TP:BZ PM is marked with green rectangles, TP:BZ F1m with pink rectangles and TP:BZ F2s with purple rectangles); C) The intensity contour plot comprising all ^{13}C{^{1}H} NMR spectra recorded as a function of time (135 – 124 ppm region, d_1 = 3 s); D) Changes in the peak width (Hz) of the ^{1}H NMR signal assigned to BZ molecule observed during the TP:BZ MeOD CLASSIC NMR experiment.

5.2 TP:BZ – TOL_d

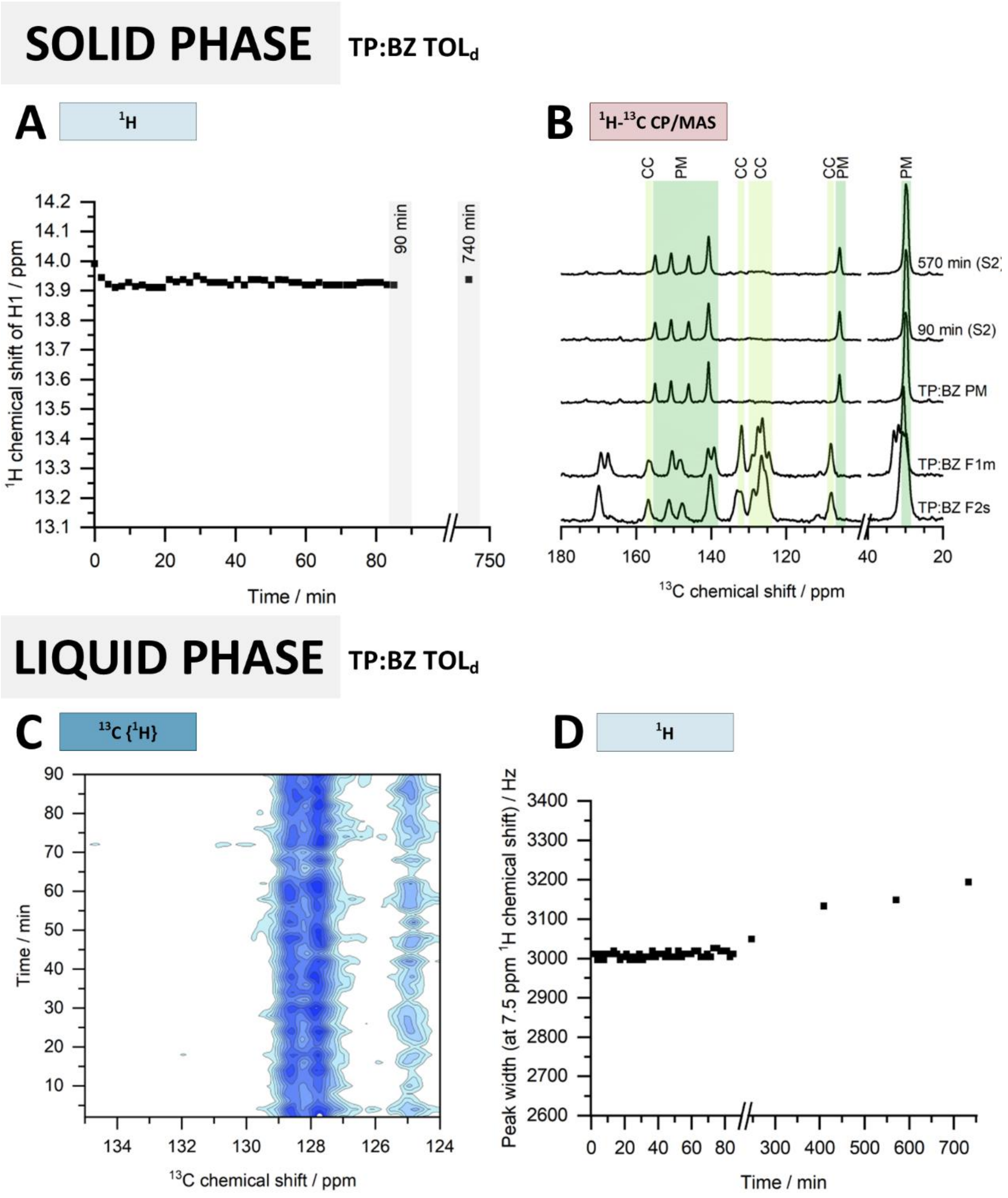

Figure S14. A) The ^{1}H NMR chemical shift (ppm) of the H1 proton of the TP:BZ system monitored during the CLASSIC NMR experiment conducted with the addition of TOL_d; B) The ^{1}H-^{13}C CP/MAS NMR spectra of TP:BZ TOL_d: 90 – 250 and 570 – 740 minutes into the CLASSIC NMR experiments compared with the reference spectra (TP:BZ PM is marked with green rectangles and TP:BZ cocrystal with lime green rectangles); C) The intensity contour plot comprising all ^{13}C{^{1}H} NMR spectra recorded as a function of time (135 – 124 ppm region, d_1 = 3 s); D) Changes in the peak width (Hz) of the ^{1}H NMR signal assigned to BZ molecule observed during the TP:BZ TOL_d CLASSIC NMR experiment.

The TP:BZ TOL_d experiment was expected to result in the formation of TP:BZ F1m due to the low polarity of TOL.[11,28] However, contrary to the TP:BZ D_2O and TP:BZ MeOD datasets, the cocrystallisation

process could not be observed based on the chemical shift changes of the H1 proton as the peak position remained constant (**Figure S14A**). The analysis of the first ^{1}H-^{13}C CP/MAS NMR spectrum (CLASSIC S2, 90 – 250 min) did not indicate any phase transformations, therefore, additional acquisitions were collected. The final spectra acquired in the sequence (CLASSIC-S2, 570 – 740 min) showed minor traces of the TP:BZ cocrystal, however, in the spectral regions that do not allow for differentiation between TP:BZ F1m and TP:BZ F2m (**Figure S14B**). Inspection of the liquid phase data, *i.e.* $^{13}C\{^{1}H\}$ NMR, revealed no dissolution of TP or BZ in TOL_d (**Figure S14C**), which is in agreement with their established poor solubility in toluene (**Figure S5**). The peaks in the $^{13}C\{^{1}H\}$ NMR spectra presented the constant intensity throughout the experiment and were attributed to TOL_d. These could be mistaken for dissolved BZ as they appear in a similar spectral range. However, BZ presents additional peaks in the 135 – 131 ppm region, which were also visible in the D_2O and MeOD datasets upon BZ dissolution (**Figure 6E, Figure S13C**). Furthermore, the width of the BZ signal peak did not indicate cocrystallisation until the 90 min mark (**Figure S14D**). After that point, gradual peak broadening became noticeable. The comparison of the peak width of neat TP, TP:BZ F1m and TP:BZ F2s revealed the increase upon cocrystallisation from 3448 Hz to 3723 Hz and 3784 Hz, respectively. This could suggest that the width of the peak (FWHM) could be the parameter which is the most susceptible to phase changes when analysing a mixture of phases. It reflects transitions that are almost invisible in the ^{1}H-^{13}C CP/MAS NMR spectra and unnoticeable in the H1 proton signal shift. Regardless, the TP:BZ cocrystallisation rate is significantly lower for low polarity solvents (TOL_d) compared to polar ones (D_2O, MeOD).

5.3 MNZ:GAL – D2O

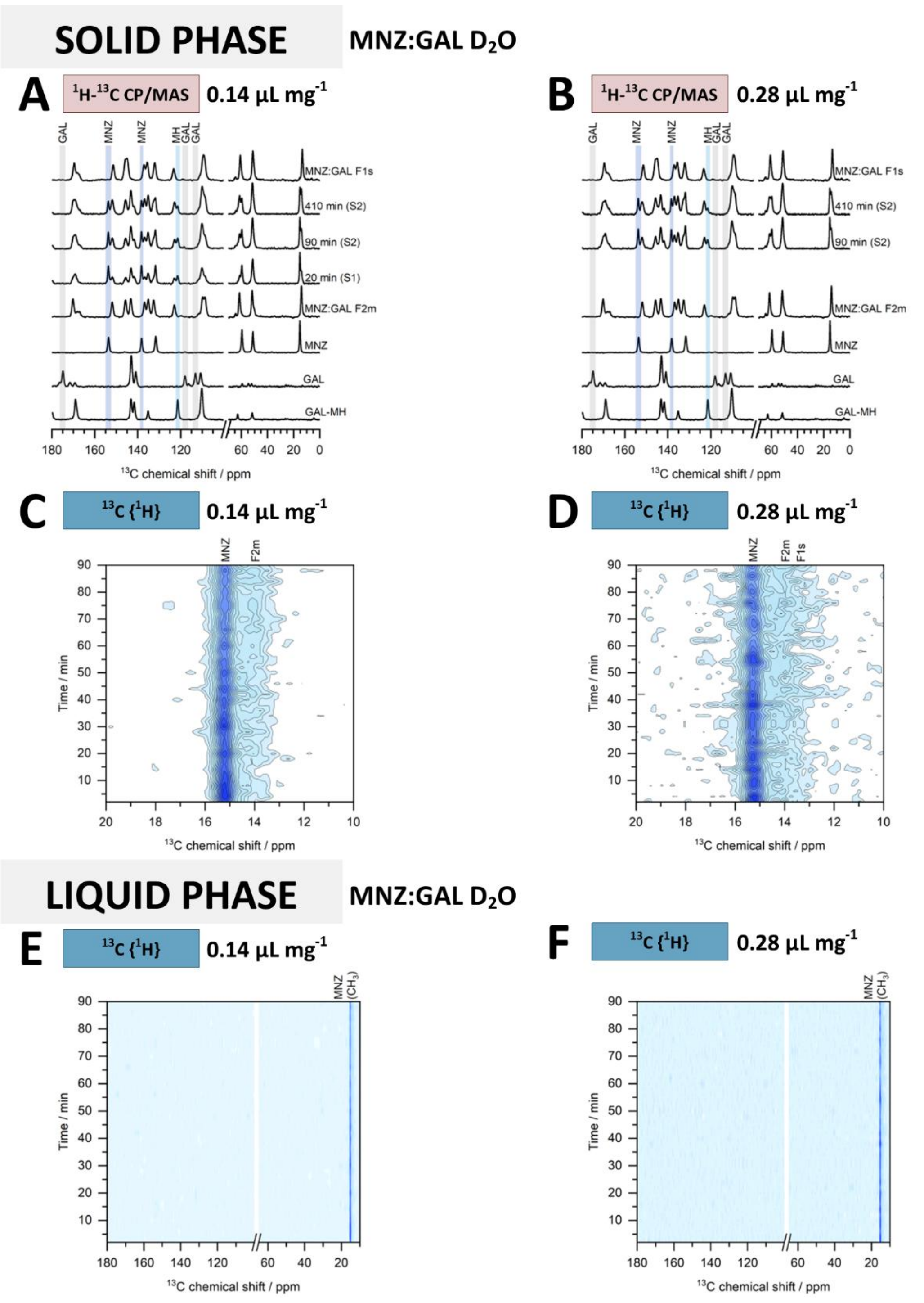

Figure S15. The ^{1}H-^{13}C CP/MAS NMR spectra of MNZ:GAL D_2O: 20 – 80, 90 – 250 and 410 – 570 minutes into the CLASSIC NMR experiments compared with the reference spectra (GAL is marked with grey rectangles, GAL-MH with a light blue rectangle and MNZ with violet rectangles) acquired with A) 0.14 µL mg^{-1} and B) 0.28 µL mg^{-1} D_2O-to-powders ratio; The intensity contour plot comprising all MNZ:GAL D_2O $^{13}C\{^{1}H\}$ NMR spectra (d_1 = 3 s) recorded as a function of time: C) 0.14 µL mg^{-1} (methyl group region 20 – 10 ppm), D) 0.28 µL mg^{-1} (methyl group region 20 – 10 ppm), E) 0.14 µL mg^{-1} and F) 0.28 µL mg^{-1}.

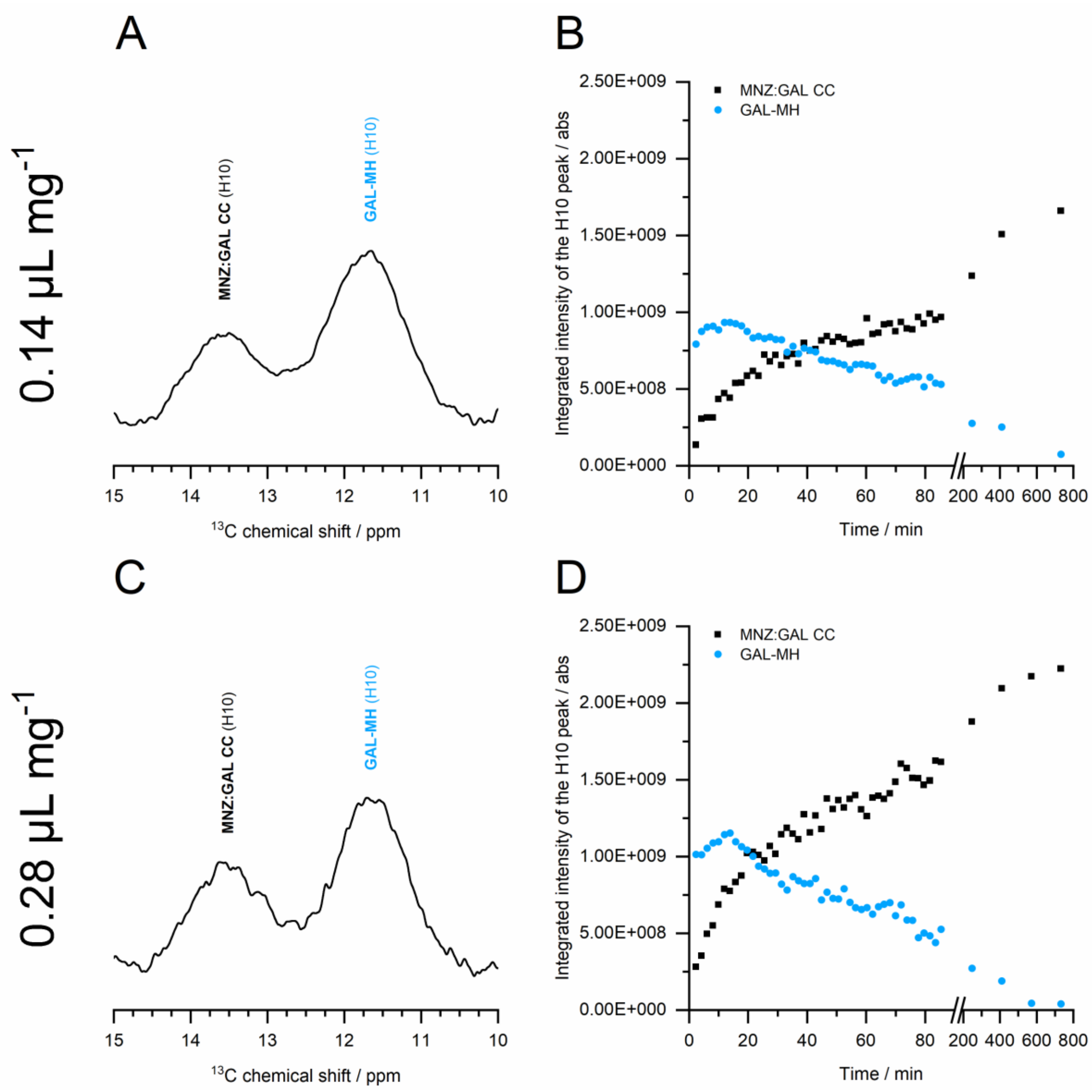

Figure S16. A) The 15 – 10 ppm region of the ^1H NMR spectrum (MNZ:GAL D_2O, 0.14 µL mg^{-1}, 10 min into CLASSIC NMR experiment) presenting peaks assigned to MNZ:GAL cocrystal (MNZ:GAL CC) and GAL-MH and B) the changes in the integrated intensity (abs) of the H10 peaks assigned to MNZ:GAL CC (black) and GAL-MH (blue). C) The 15 – 10 ppm region of the ^1H NMR spectrum (MNZ:GAL D_2O, 0.28 µL mg^{-1}, 10 min into CLASSIC NMR experiment) presenting peaks assigned to MNZ:GAL cocrystal (MNZ:GAL CC) and GAL-MH and D) the changes in the integrated intensity (abs) of the H10 peaks assigned to MNZ:GAL CC (black) and GAL-MH (blue).

The difference in position of H10 peak in GAL-MH (^1H NMR, H10: 11.74 ppm) and the metastable polymorph of MNZ:GAL F2m cocrystal (^1H NMR, H10: 13.85 ppm) peaks is greater than 2 ppm. Consequently, in ^1H NMR spectra of MNZ:GAL D_2O CLASSIC NMR two distinct peaks are observable in the region assigned to H10 (**Figure S16A**). It is worth noting that the structural similarity between the two MNZ:GAL cocrystal polymorphs is reflected both in ^1H-^{13}C CP/MAS NMR and ^1H MAS NMR spectra. Thus, in the ^1H NMR spectra, the H10 resonates at 13.85 and 13.98 ppm in MNZ:GAL F2m and MNZ:GAL F1s, respectively. Therefore, ^1H NMR enables monitoring of the cocrystallisation process (*i.e.* the

growth of a ^{1}H NMR peak at *ca.* 13.9 ppm) but not differentiating between the two MNZ:GAL cocrystal polymorphs. The second upfield peak could be produced by either neat GAL (12.43 ppm) or GAL-MH (11.74 ppm) but in the first spectrum recorded during the MNZ:GAL D_2O CLASSIC NMR sequence, this peak was positioned at 11.75 ppm proving the immediate formation of GAL-MH upon contact with D_2O. Further analysis of the integrated intensities of both peaks (**Figure S16B**) revealed that the maximum intensity for the GAL-MH peak was reached after 15 min which supports this observation, arising from the ^{1}H-^{13}C CP/MAS NMR analysis, that the GAL-MH formation completed before the 20 min mark. Followingly, a decrease in the GAL-MH peak intensity was noted, alongside gradual increase in the MNZ:GAL cocrystal intensity which results from GAL-MH consumption during MNZ:GAL cocrystal formation.

5.4 MNZ:GAL – MeOD

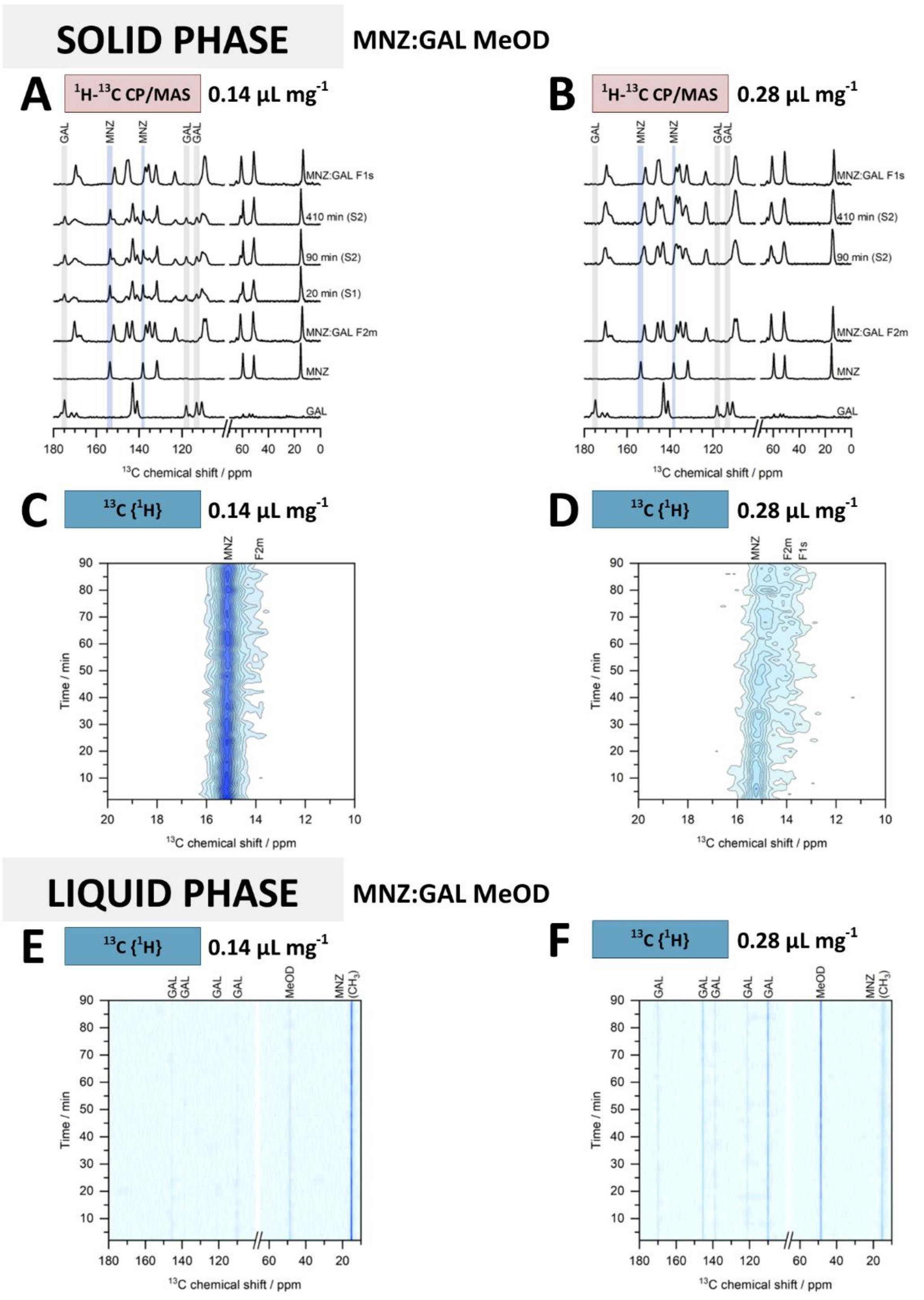

Figure S17. The ^{1}H-^{13}C CP/MAS NMR spectra of MNZ:GAL MeOD: 20 – 80, 90 – 250 and 410 – 570 minutes into the CLASSIC NMR experiments compared with the reference spectra (GAL is marked with grey rectangles and MNZ with violet rectangles) acquired with A) 0.14 µL mg^{-1} and B) 0.28 µL mg^{-1} MeOD-to-powders ratio; The intensity contour plot comprising all MNZ:GAL MeOD $^{13}C\{^{1}H\}$ NMR spectra (d_1 = 3 s) recorded as a function of time: C) 0.14 µL mg^{-1} (methyl group region 20 – 10 ppm), D) 0.28 µL mg^{-1} (methyl group region 20 – 10 ppm), E) 0.14 µL mg^{-1} and F) 0.28 µL mg^{-1}.

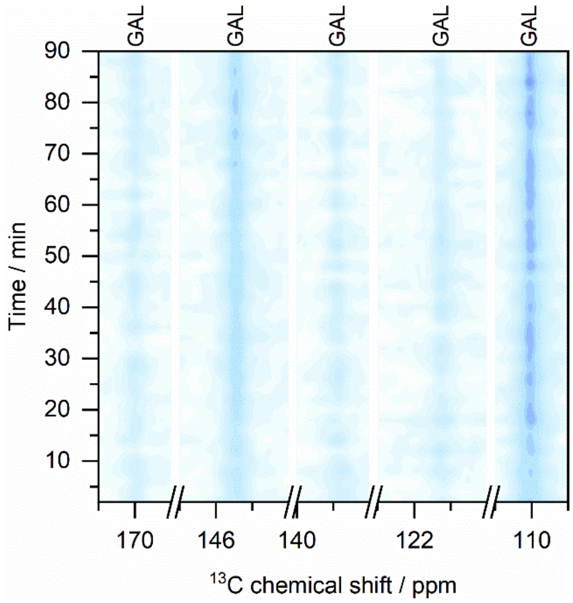

Figure S18. The intensity contour plot comprising all MNZ:GAL MeOD $^{13}C\{^1H\}$ MAS NMR spectra (d_1 = 3 s) recorded as a function of time (0.28 µL mg^{-1}). Compared to the **Figure S17F**, this graph was zoomed into the signals assigned to GAL (*ca.* 110, 121, 139, 145, 170 ppm).

Table S5. The area under the curve measured for the MNZ CH_3 peaks(^1H – ^{13}C CP/MAS NMR) at 90 min (CLASSIC-S2, 90 – 250 min) and 410 min (CLASSIC-S2, 410 – 570 min) mark.

	MNZ:GAL D_2O		MNZ:GAL MeOD	
	0.14 µL mg^{-1}	0.28 µL mg^{-1}	0.14 µL mg^{-1}	0.28 µL mg^{-1}
90 min	1.06×10^{12}	8.72×10^{11}	1.08×10^{12}	8.39×10^{11}
410 min	1.06×10^{12}	8.29×10^{11}	9.78×10^{11}	6.43×10^{11}
Δ	0.00 (0%)	0.43×10^{11} (4.9%)	1.02×10^{11} (9.4%)	1.96×10^{11} (23.4%)

5.5 MNZ:GAL - TOL_d

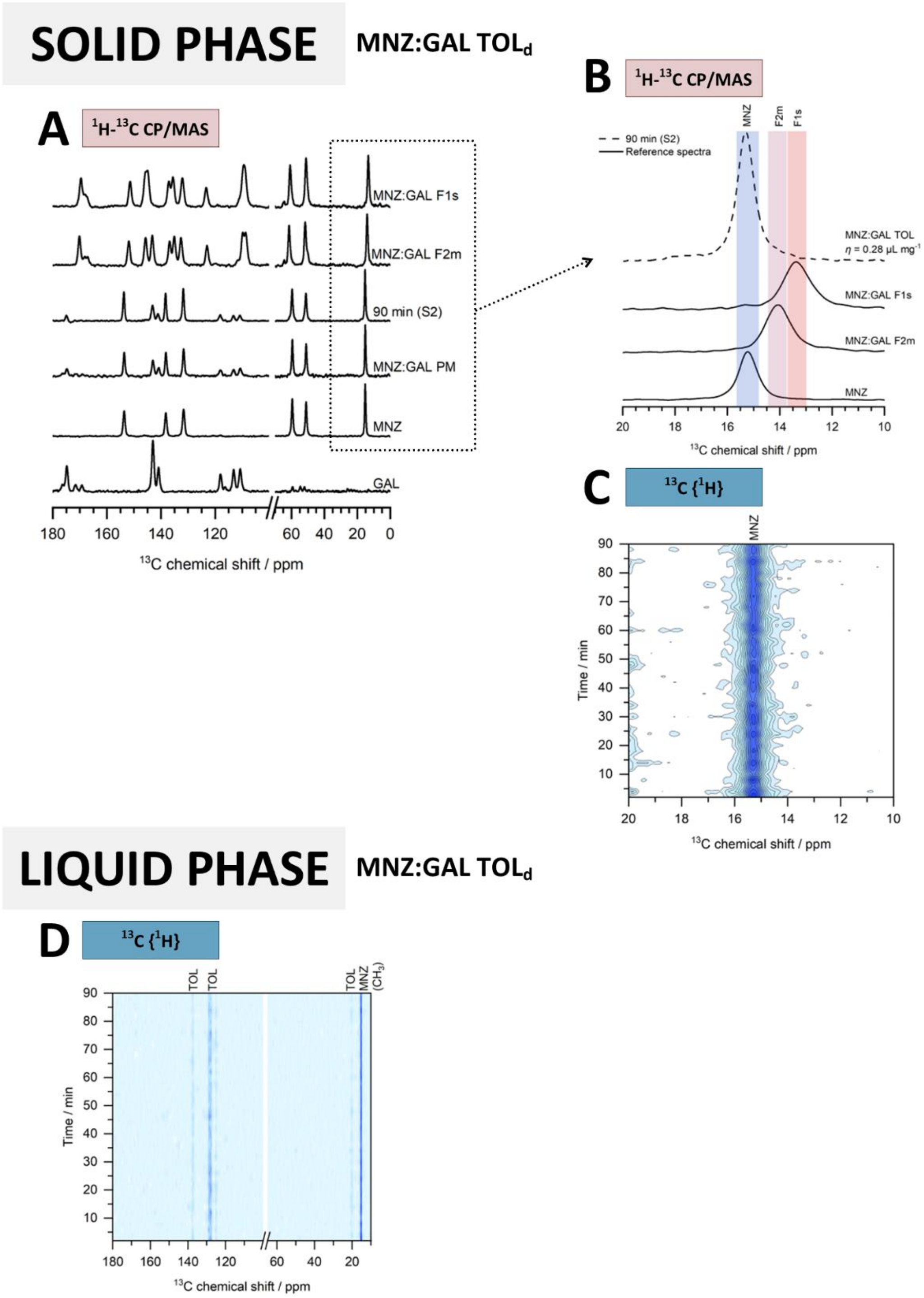

Figure S19. The ^{1}H-^{13}C CP/MAS NMR spectrum (20 – 80 minutes into the CLASSIC NMR experiment) of MNZ:GAL TOL: A) compared with the reference spectra (GAL, MNZ, MNZ:GAL PM, MNZ:GAL F2m, MNZ:GAL F1s) and B) zoomed into the region assigned to the methyl group (C4) of MNZ (10 – 20 ppm) compared with the reference spectra of MNZ (violet), MNZ:GAL F2m (cool pink) and MNZ:GAL F1s (warm pink); The intensity contour plot comprising all MNZ:GAL TOL ^{13}C{^{1}H} NMR spectra (d_1 = 3 s) recorded as a function of time: C) the methyl group region (20 – 10 ppm) and D) 180 – 10 ppm. The data were acquired with 0.28 µL mg^{-1} TOL-to-powders ratio.

Based on preliminary TP:BZ D_2O results and the 0.14 µL mg^{-1} ratio used for MNZ:GAL D_2O and MNZ:GAL MeOD systems, only the 0.28 µL mg^{-1} ratio experiment was performed for MNZ:GAL TOL_d with a single ^1H-^{13}C CP/MAS NMR spectrum acquired at 90 min (CLASSIC-S2, 90 – 250 min). Both the whole-range spectrum (**Figure S19A**) and the MNZ methyl region (**Figure S19B, Figure S19C**) confirm that no cocrystallisation of the MNZ:GAL system occurred in the presence of TOL_d. In the $^{13}C\{^1H\}$ NMR liquid-like spectra, no dissolution was detected as the only visible peaks were assigned to the MNZ methyl group and the solvent. The obtained data do not contradict the possible formation of the MNZ:GAL cocrystal upon the addition of TOL_d, as in the TP:BZ system the indication of cocrystal formation appeared after 250 min (**Figure S14D**).

5.6 Representative ^1H NMR spectra (H1 position)

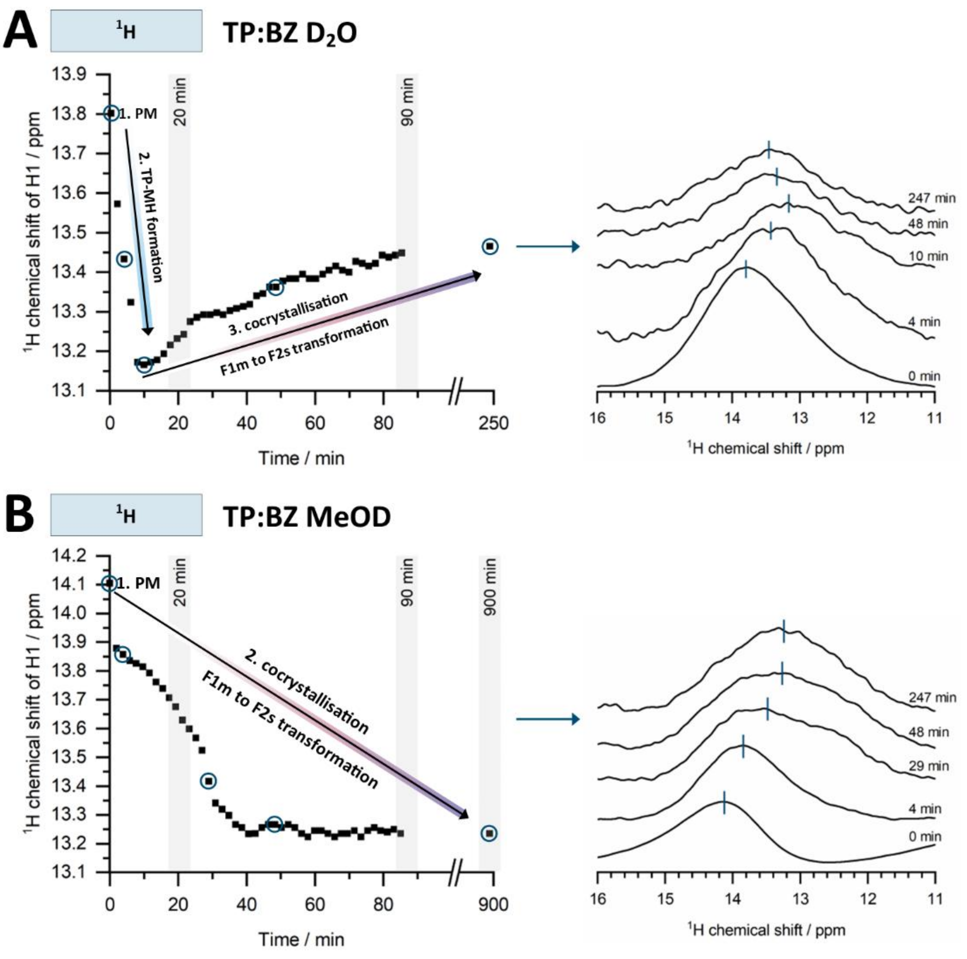

Figure S20. Changes in the ^1H NMR chemical shift (ppm) of the H1 proton of the TP:BZ system monitored during the CLASSIC NMR experiment conducted with the addition of A) D_2O and B) MeOD with the corresponding ^1H NMR spectra at representative time points.

6 References

(1) Ebisuzaki, Y.; Boyle, P. D.; Smith, J. A. Methylxanthines. I. Anhydrous Theophylline. *Acta Crystallogr., Sect. C:Cryst. Struct. Commun.* **1997**, *53* (6), 777–779. https://doi.org/10.1107/S0108270197001960.

(2) Liu, H.; Stephen Chan, H. C.; Zhang, L.; Lu, Y.; Li, J.; Li, J.; Li, L.; Zhou, Z. The Molecular Mechanisms of Plasticity in Crystal Forms of Theophylline. *Chin. Chem. Lett.* **2023**, *34* (8), 108057. https://doi.org/10.1016/j.cclet.2022.108057.

(3) Blake, C. C. F.; Small, R. W. H. The Crystal Structure of Benzamide. *Acta Crystallogr., Sect. B* **1972**, *28* (7), 2201–2206. https://doi.org/10.1107/S0567740872005801.

(4) Fischer, F.; Schmidt, M. U.; Greiser, S.; Emmerling, F. The Challenging Case of the Theophylline–Benzamide Cocrystal. *Acta Crystallogr., Sect. C:Struct. Chem.* **2016**, *72* (3), 217–224. https://doi.org/10.1107/S2053229616002643.

(5) Fischer, F.; Heidrich, A.; Greiser, S.; Benemann, S.; Rademann, K.; Emmerling, F. Polymorphism of Mechanochemically Synthesized Cocrystals: A Case Study. *Cryst. Growth Des.* **2016**, *16* (3), 1701–1707. https://doi.org/10.1021/acs.cgd.5b01776.

(6) Blaton, N. M.; Peeters, O. M.; De Ranter, C. J. 2-(2-Methyl-5-Nitro-1-Imidazolyl)Ethanol (Metronidazole). *Acta Crystallogr., Sect. B* **1979**, *35* (10), 2465–2467. https://doi.org/10.1107/S0567740879009663.

(7) Braun, D. E.; Bhardwaj, R. M.; Florence, A. J.; Tocher, D. A.; Price, S. L. Complex Polymorphic System of Gallic Acid—Five Monohydrates, Three Anhydrates, and over 20 Solvates. *Cryst. Growth Des.* **2013**, *13* (1), 19–23. https://doi.org/10.1021/cg301506x.

(8) Okabe, N.; Kyoyama, H.; Suzuki, M. Gallic Acid Monohydrate. *Acta Crystallogr., Sect. E:Struct. Rep. Online* **2001**, *57* (8), 764–766. https://doi.org/10.1107/S1600536801012041.

(9) Zheng, K.; Li, A.; Wu, W.; Qian, S.; Liu, B.; Pang, Q. Preparation, Characterization, in Vitro and in Vivo Evaluation of Metronidazole–Gallic Acid Cocrystal: A Combined Experimental and Theoretical Investigation. *J. Mol. Struct.* **2019**, *1197*, 727–735. https://doi.org/10.1016/j.molstruc.2019.07.102.

(10) Seera, R.; Guru Row, T. N. Evaluation of Cocrystallization Outcomes of Multicomponent Adducts: Rapid Fabrication to Achieve Uniform Particle Size Distribution Using Thermal Inkjet Printing. *Cryst. Growth Des.* **2020**, *20* (7), 4667–4677. https://doi.org/10.1021/acs.cgd.0c00469.

(11) Dyba, A. J.; Wiącek, E.; Nowak, M.; Janczak, J.; Nartowski, K. P.; Braun, D. E. Metronidazole Cocrystal Polymorphs with Gallic and Gentisic Acid Accessed through Slurry, Atomization Techniques, and Thermal Methods. *Cryst. Growth Des.* **2023**, *23* (11), 8241–8260. https://doi.org/10.1021/acs.cgd.3c00951.

(12) Blagden, N.; Davey, R.; Dent, G.; Song, M.; David, W. I. F.; Pulham, C. R.; Shankland, K. Woehler and Liebig Revisited: A Small Molecule Reveals Its Secrets - The Crystal Structure of the Unstable Polymorph of Benzamide Solved after 173 Years. *Cryst. Growth Des.* **2005**, *5* (6), 2218–2224. https://doi.org/10.1021/cg050187b.

(13) Thun, J.; Seyfarth, L.; Senker, J.; Dinnebier, R. E.; Breu, J. Polymorphism in Benzamide: Solving a 175-Year-Old Riddle. *Angew. Chem., Int. Ed.* **2007**, *46* (35), 6729–6731. https://doi.org/10.1002/anie.200701383.

(14) Fucke, K.; McIntyre, G. J.; Wilkinson, C.; Henry, M.; Howard, J. A. K.; Steed, J. W. New Insights into an Old Molecule: Interaction Energies of Theophylline Crystal Forms. *Cryst. Growth Des.* **2012**, *12* (3), 1395–1401. https://doi.org/10.1021/cg201499s.

(15) Pinon, A. C.; Rossini, A. J.; Widdifield, C. M.; Gajan, D.; Emsley, L. Polymorphs of Theophylline Characterized by DNP Enhanced Solid-State NMR. *Mol. Pharmaceutics* **2015**, *12* (11), 4146–4153. https://doi.org/10.1021/acs.molpharmaceut.5b00610.

(16) Reichardt, C. Solvatochromic Dyes as Solvent Polarity Indicators. *Chem. Rev.* **1994**, *94* (8), 2319–2358. https://doi.org/10.1021/cr00032a005.

(17) Fernandes, J. A.; Sardo, M.; Mafra, L.; Choquesillo-Lazarte, D.; Masciocchi, N. X-Ray and NMR Crystallography Studies of Novel Theophylline Cocrystals Prepared by Liquid Assisted Grinding. *Cryst. Growth Des.* **2015**, *15* (8), 3674–3683. https://doi.org/https://doi.org/10.1021/acs.cgd.5b00279.

(18) Bruni, G.; Monteforte, F.; Maggi, L.; Friuli, V.; Ferrara, C.; Mustarelli, P.; Girella, A.; Berbenni, V.; Capsoni, D.; Milanese, C.; Marini, A. Probenecid and Benzamide: Cocrystal Prepared by a Green Method and Its Physico-Chemical and Pharmaceutical Characterization. *J. Therm. Anal. Calorim.* **2020**, *140* (4), 1859–1869. https://doi.org/10.1007/s10973-019-09197-2.

(19) González-González, J. S.; Martínez-Santos, A.; Emparán-Legaspi, M. J.; Pineda-Contreras, A.; Martínez-Martínez, F. J.; Flores-Alamo, M.; García-Ortega, H. Molecular Structure and Selective Theophylline Complexation by Conformational Change of Diethyl *N*,*N*′-(1,3-

Phenylene)Dicarbamate. *Acta Crystallogr., Sect. C:Struct. Chem.* **2024**, *80* (6), 190–199. https://doi.org/10.1107/S2053229624003358.

(20) Wang, L.; Luo, M.; Li, J.; Wang, J.; Zhang, H.; Deng, Z. Sweet Theophylline Cocrystal with Two Tautomers of Acesulfame. *Cryst. Growth Des.* **2015**, *15* (6), 2574–2578. https://doi.org/10.1021/acs.cgd.5b00207.

(21) Pindelska, E.; Sokal, A.; Szeleszczuk, L.; Pisklak, D. M.; Kolodziejski, W. Solid-State NMR Studies of Theophylline Co-Crystals with Dicarboxylic Acids. *J. Pharm. Biomed. Anal.* **2014**, *100*, 322–328. https://doi.org/10.1016/j.jpba.2014.07.011.

(22) Li, P.; Chu, Y.; Wang, L.; Wenslow, R. M.; Yu, K.; Zhang, H.; Deng, Z. Structure Determination of the Theophylline–Nicotinamide Cocrystal: A Combined Powder XRD, 1D Solid-State NMR, and Theoretical Calculation Study. *CrystEngComm* **2014**, *16* (15), 3141–3147. https://doi.org/10.1039/C4CE00012A.

(23) Rossi, F.; Cerreia Vioglio, P.; Bordignon, S.; Giorgio, V.; Nervi, C.; Priola, E.; Gobetto, R.; Yazawa, K.; Chierotti, M. R. Unraveling the Hydrogen Bond Network in a Theophylline–Pyridoxine Salt Cocrystal by a Combined X-Ray Diffraction, Solid-State NMR, and Computational Approach. *Cryst. Growth Des.* **2018**, *18* (4), 2225–2233. https://doi.org/10.1021/acs.cgd.7b01662.

(24) Wang, X.; Kong, M.; Li, D.; Fang, J.; Deng, Z.; Zhang, H. Stanozolol–Aromatic Carboxylic Acid Crystalline Complexes: Flexible Tautomeric/Ionization States and Supramolecular Synthons. *CrystEngComm* **2019**, *21* (13), 2144–2153. https://doi.org/10.1039/C8CE01439F.

(25) Wawer, I.; Zielinska, A. 13C-CP-MAS-NMR Studies of Flavonoids. I. Solid-State Conformation of Quercetin, Quercetin 5′-Sulphonic Acid and Some Simple Polyphenols. *Solid State Nucl. Magn. Reson.* **1997**, *10* (1–2), 33–38. https://doi.org/10.1016/S0926-2040(97)00018-0.

(26) Thun, J.; Seyfarth, L.; Butterhof, C.; Senker, J.; Dinnebier, R. E.; Breu, J. Wöhler and Liebig Revisited: 176 Years of Polymorphism in Benzamide - and the Story Still Continues! *Cryst. Growth Des.* **2009**, *9* (5), 2435–2441. https://doi.org/10.1021/cg801347d.

(27) Lampronti, G. I.; Michalchuk, A. A. L.; Mazzeo, P. P.; Belenguer, A. M.; Sanders, J. K. M.; Bacchi, A.; Emmerling, F. Changing the Game of Time Resolved X-Ray Diffraction on the Mechanochemistry Playground by Downsizing. *Nat. Commun.* **2021**, *12* (1), 6134. https://doi.org/10.1038/s41467-021-26264-1.

(28) Trask, A. V.; Shan, N.; Motherwell, W. D. S.; Jones, W.; Feng, S.; Tan, R. B. H.; Carpenter, K. J. Selective Polymorph Transformation via Solvent-Drop Grinding. *Chem. Commun.* **2005**, No. 7, 880. https://doi.org/10.1039/b416980h.
